# Validation of models to diagnose ovarian cancer in patients managed surgically or conservatively: multicentre cohort study

**DOI:** 10.1136/bmj.m2614

**Published:** 2020-07-30

**Authors:** Ben Van Calster, Lil Valentin, Wouter Froyman, Chiara Landolfo, Jolien Ceusters, Antonia C Testa, Laure Wynants, Povilas Sladkevicius, Caroline Van Holsbeke, Ekaterini Domali, Robert Fruscio, Elisabeth Epstein, Dorella Franchi, Marek J Kudla, Valentina Chiappa, Juan L Alcazar, Francesco P G Leone, Francesca Buonomo, Maria Elisabetta Coccia, Stefano Guerriero, Nandita Deo, Ligita Jokubkiene, Luca Savelli, Daniela Fischerová, Artur Czekierdowski, Jeroen Kaijser, An Coosemans, Giovanni Scambia, Ignace Vergote, Tom Bourne, Dirk Timmerman

**Affiliations:** 1 Department of Development and Regeneration, KU Leuven, Herestraat 49 Box 805, 3000 Leuven, Belgium; 2 Department of Biomedical Data Sciences, Leiden University Medical Centre, Leiden, Netherlands; 3 EPI-Centre, KU Leuven, Leuven, Belgium; 4 Department of Obstetrics and Gynaecology, Skåne University Hospital, Malmö, Sweden; 5 Department of Clinical Sciences Malmö, Lund University, Lund, Sweden; 6 Department of Obstetrics and Gynaecology, University Hospitals Leuven, Leuven, Belgium; 7 Queen Charlotte’s and Chelsea Hospital, Imperial College, London, UK; 8 Laboratory of Tumour Immunology and Immunotherapy, Department of Oncology, KU Leuven, Leuven, Belgium; 9 Department of Woman, Child and Public Health, Fondazione Policlinico Universitario Agostino Gemelli, Istituto di Ricovero e Cura a Carattere Scientifico, Rome, Italy; 10 Department of Life Science and Public Health, Universita’ Cattolica del Sacro Cuore, Rome, Italy; 11 Department of Epidemiology, CAPHRI Care and Public Health Research Institute, Maastricht University, Maastricht, Netherlands; 12 Department of Obstetrics and Gynaecology, Ziekenhuis Oost-Limburg, Genk, Belgium; 13 First Department of Obstetrics and Gynaecology, Alexandra Hospital, Medical School, National and Kapodistrian University of Athens, Athens, Greece; 14 Clinic of Obstetrics and Gynaecology, University of Milan-Bicocca, San Gerardo Hospital, Monza, Italy; 15 Department of Clinical Science and Education, Karolinska Institutet, Stockholm, Sweden; 16 Department of Obstetrics and Gynaecology, Södersjukhuset, Stockholm, Sweden; 17 Preventive Gynaecology Unit, Division of Gynaecology, European Institute of Oncology IRCCS, Milan, Italy; 18 Department of Perinatology and Oncological Gynaecology, School of Health Sciences in Katowice, Medical University of Silesia, Katowice, Poland; 19 Department of Gynaecologic Oncology, National Cancer Institute of Milan, Milan, Italy; 20 Department of Obstetrics and Gynaecology, Clinica Universidad de Navarra, School of Medicine, Pamplona, Spain; 21 Department of Obstetrics and Gynaecology, Biomedical and Clinical Sciences Institute L. Sacco, University of Milan, Milan, Italy; 22 Institute for Maternal and Child Health, IRCCS Burlo Garofolo, Trieste, Italy; 23 Department of Experimental and Clinical Biomedical Sciences, University of Florence, Florence, Italy; 24 Department of Obstetrics and Gynaecology, University of Cagliari, Policlinico Universitario Duilio Casula, Monserrato, Cagliari, Italy; 25 Department of Obstetrics and Gynaecology, Whipps Cross Hospital, London, UK; 26 Department of Obstetrics and Gynaecology, University of Bologna, Bologna, Italy; 27 Gynaecological Oncology Centre, Department of Obstetrics and Gynaecology, First Faculty of Medicine, Charles University and General University Hospital, Prague, Czech Republic; 28 First Department of Gynaecological Oncology and Gynaecology, Medical University of Lublin, Lublin, Poland; 29 Department of Obstetrics and Gynaecology, Ikazia Hospital, Rotterdam, Netherlands; 30 Leuven Cancer Institute, University Hospitals Leuven, Leuven, Belgium

## Abstract

**Objective:**

To evaluate the performance of diagnostic prediction models for ovarian malignancy in all patients with an ovarian mass managed surgically or conservatively.

**Design:**

Multicentre cohort study.

**Setting:**

36 oncology referral centres (tertiary centres with a specific gynaecological oncology unit) or other types of centre.

**Participants:**

Consecutive adult patients presenting with an adnexal mass between January 2012 and March 2015 and managed by surgery or follow-up.

**Main outcome measures:**

Overall and centre specific discrimination, calibration, and clinical utility of six prediction models for ovarian malignancy (risk of malignancy index (RMI), logistic regression model 2 (LR2), simple rules, simple rules risk model (SRRisk), assessment of different neoplasias in the adnexa (ADNEX) with or without CA125). ADNEX allows the risk of malignancy to be subdivided into risks of a borderline, stage I primary, stage II-IV primary, or secondary metastatic malignancy. The outcome was based on histology if patients underwent surgery, or on results of clinical and ultrasound follow-up at 12 (±2) months. Multiple imputation was used when outcome based on follow-up was uncertain.

**Results:**

The primary analysis included 17 centres that met strict quality criteria for surgical and follow-up data (5717 of all 8519 patients). 812 patients (14%) had a mass that was already in follow-up at study recruitment, therefore 4905 patients were included in the statistical analysis. The outcome was benign in 3441 (70%) patients and malignant in 978 (20%). Uncertain outcomes (486, 10%) were most often explained by limited follow-up information. The overall area under the receiver operating characteristic curve was highest for ADNEX with CA125 (0.94, 95% confidence interval 0.92 to 0.96), ADNEX without CA125 (0.94, 0.91 to 0.95) and SRRisk (0.94, 0.91 to 0.95), and lowest for RMI (0.89, 0.85 to 0.92). Calibration varied among centres for all models, however the ADNEX models and SRRisk were the best calibrated. Calibration of the estimated risks for the tumour subtypes was good for ADNEX irrespective of whether or not CA125 was included as a predictor. Overall clinical utility (net benefit) was highest for the ADNEX models and SRRisk, and lowest for RMI. For patients who received at least one follow-up scan (n=1958), overall area under the receiver operating characteristic curve ranged from 0.76 (95% confidence interval 0.66 to 0.84) for RMI to 0.89 (0.81 to 0.94) for ADNEX with CA125.

**Conclusions:**

Our study found the ADNEX models and SRRisk are the best models to distinguish between benign and malignant masses in all patients presenting with an adnexal mass, including those managed conservatively.

**Trial registration:**

ClinicalTrials.gov NCT01698632.

## Introduction

Ovarian cancer is a gynaecological malignancy with a high mortality rate. In 2018, an estimated 295 400 women developed ovarian cancer worldwide, and 184 800 deaths were reported from the disease.^1^ The prognosis for women with ovarian cancer treated in oncology centres is better than for those managed in other settings.^2^ ^3^ ^4^ ^5^ Methods such as risk prediction models are needed to reliably estimate the likelihood that a mass is malignant so that patients can receive the optimal treatment. Risk prediction models can be used to individualise patient management, such as setting priorities on waiting lists for further investigations and specialist consultations, and deciding whether patients need surgery performed by surgeons who specialise in oncological surgery or whether surgery is not required. Adnexal masses judged to be benign can be safely managed with follow-up.^6^ If a benign mass causes symptoms it can be surgically removed in a local centre, however if malignancy is suspected the mass should be managed in an oncological referral centre.

Ultrasound based diagnostic models can be used to predict malignancy in adnexal masses. A commonly used model is the risk of malignancy index (RMI), which was developed in 1990.^7^ Newer models are the International Ovarian Tumour Analysis (IOTA) models: logistic regression model 1 (LR1), logistic regression model 2 (LR2), simple rules, simple rules risk model (SRRisk), and assessment of different neoplasias in the adnexa (ADNEX).^8^ ^9^ ^10^ ^11^ The performance of the IOTA models has been externally validated and compared on thousands of patients.^12^ ^13^ ^14^ However, only three validation studies reported on model calibration, that is, the agreement between predicted malignancy risk and observed proportion of malignancy; only one compared the clinical utility of different prediction models for referring patients with an adnexal mass to an oncology centre.^15^ ^16^ ^17^ ^18^ More importantly, all model development and validation studies, except for two small single centre validation studies,^19^ ^20^ recruited patients who underwent surgery with histology as the reference standard. Therefore, current evidence is limited to patients for whom the decision to operate had already been made. To select the optimal treatment, models should perform well on patients who undergo surgery and on those who are managed conservatively.

The primary aim of this study was to evaluate the performance of the IOTA models and RMI when applied on all adnexal masses irrespective of management, that is, conservative or surgical. The secondary aim was to evaluate model performance in clinically relevant subgroups.

## Methods

### Study design and participants

This study was conducted by using interim data from the IOTA phase 5 study (IOTA5), an international multicentre prospective cohort study.^6^ IOTA5 recruited consecutive patients with an adnexal mass examined by using transvaginal ultrasound, irrespective of whether patients subsequently underwent surgery or were managed conservatively with follow-up visits. Appendix 1 presents the IOTA5 protocol. IOTA5 recruitment took place from January 2012 to October 2016, but follow-up will continue until all patients receiving conservative management have been followed up for at least five years. The current interim analysis includes patients recruited until 1 March 2015 and follow-up data until 30 June 2017. Thirty six centres in 14 countries recruited patients to the study. The contributing centres were either oncology referral centres (tertiary centres with a specific gynaecological oncology unit) or other types of centre. We obtained approval from the ethics committee of the University Hospitals Leuven as the coordinating centre (B32220095331/S51375) and the local ethics committee of each contributing centre. We report the study according to the TRIPOD (transparent reporting of a multivariable prediction model for individual prognosis or diagnosis) guidelines.^21^

Patients were eligible if they were aged 18 or older at recruitment and presented with at least one adnexal mass (ovarian, para-ovarian, or tubal) on ultrasound examination. Informed consent was obtained and then local clinicians examined patients following a standardised research protocol. Exclusion criteria were lesions presumed to be physiological if the largest diameter was less than 3 cm, refusal to provide informed consent, or withdrawal of informed consent. Pregnancy was not an exclusion criterion. We excluded patients if they had an adnexal mass that was already being followed up in the recruitment centre before the start of the study.

### Procedures

The ultrasound examiners who recruited participants followed the standardised research protocol. They collected clinical information and performed a transvaginal ultrasound examination, and an abdominal scan if necessary. The examination consisted of scanning the uterus, both adnexa, and the whole pelvis outside these organs. Grey scale and colour or power Doppler ultrasound was used to characterise the morphology and vascularisation of the adnexal mass. Examiners collected information on several predefined ultrasound variables, including those used in the prediction models, and ultrasound results were described by using IOTA terminology.^22^

We had no requirements about the level of experience of the ultrasound examiners, but all examiners were IOTA trainers or had passed the IOTA certification test (https://www.iotagroup.org/certified-members). Ultrasound examiners used subjective assessment of the ultrasound images to classify lesions as benign, borderline, or malignant, and specified the degree of certainty with which the classification was made (certain, probable, or uncertain). The presumed histology was registered according to a list of 18 predefined diagnoses. These diagnoses were based on knowledge of the typical ultrasound appearance of benign, borderline, and malignant lesions, and of different types of specific adnexal pathology.^23^ When examiners detected multiple masses, the dominant mass was defined as the mass with the most complex ultrasound morphology. If multiple masses had similar morphology, the largest mass or the mass that was most accessible with ultrasound was denoted dominant. We used the dominant mass in our statistical analyses. The ultrasound examiner suggested surgery or conservative management based on the ultrasound diagnosis and the patient’s symptoms. Ultimately, however, the treating clinician decided upon the management strategy together with the patient. Therefore, the suggested management and actual management might be different. We encouraged centres to measure the level of serum CA125 in all patients, but this was not a requirement for inclusion in the study. Measurement of CA125 was left to clinical judgment and local protocols.

Conservative management included ultrasound and clinical follow-up at intervals of three months, six months, and then every 12 months thereafter. At follow-up visits clinical information including symptoms was collected and an ultrasound examination was performed in the same manner as at the inclusion scan. Examiners collected data on several predefined ultrasound variables and suggested a diagnosis by subjectively assessing the ultrasound images. After one or more follow-up visits, some patients underwent surgery for a variety of reasons (eg, suspicion of malignancy or patient anxiety).^6^ For some patients, the mass resolved spontaneously during follow-up.

Each centre performed surgery by following local protocols and histological examination of surgically removed masses. We did not carry out a central pathology review because in a previous study we did not observe important differences in reported outcomes between local and central pathology reports.^8^ We classified malignant tumours according to the criteria recommended by the International Federation of Gynaecology and Obstetrics.^24^

### Data cleaning

We collected patient level data by using a secure electronic platform developed for the study (IOTA5 Study Screen; astraia software, Munich, Germany). Patients automatically received a unique identifier upon enrolment. We encrypted all data communication to ensure data security. A team of biostatisticians and ultrasound examiners performed data cleaning. Data cleaning included sending queries to participating centres to retrieve missing information or to correct inconsistencies. Local centres used a standardised questionnaire (appendix 2) to accrue missing information by telephoning patients and managing clinicians.

For the primary analysis, we excluded centres that recruited fewer than 50 patients, those that recruited non-consecutively (focused only on patients who underwent surgery without follow-up, or only on patients managed conservatively), and those that provided poor follow-up information for more than 30% of patients (supplementary table 1, appendix 3).^6^ Poor follow-up information was defined as the absence of a study outcome (spontaneous resolution or histology based on surgery at any point during follow-up) and last follow-up visit less than ten months after inclusion.

### Prediction models

We evaluated several ultrasound based prediction models: RMI, LR2, simple rules, SRRisk, ADNEX without CA125, and ADNEX with CA125 (table 1). Model predictions are based on information obtained at the inclusion scan and so are blinded to the outcome. RMI does not give an estimated risk, but a non-negative integer (0 or higher), with higher scores suggesting a higher likelihood of malignancy. LR2 and SRRisk calculate the risk that the tumour is malignant. ADNEX calculates the probability of five outcome categories: benign, borderline, stage I primary invasive ovarian malignancy, stage II-IV primary invasive ovarian malignancy, and metastasis in the adnexa from another primary tumour (eg, breast cancer). For ADNEX, one minus the probability of a benign tumour equals the estimated risk of malignancy. The simple rules classify tumours as benign, inconclusive, or malignant based on the presence of five typical ultrasound features of benign tumours and five typical ultrasound features of malignant tumours. The prediction is inconclusive when none of the 10 features is present, or when a mixture of benign and malignant features is present. Here, we add inconclusive tumours to those predicted to be malignant, resulting in a binary classifier. Appendix 4 gives details of predictors and model formulas.

**Table 1:** Summary of diagnostic prediction models for ovarian malignancy

Model	Type	Predictor variables	Comments
RMI	Score	CA125, menopausal status, ultrasound score based on five binary ultrasound variables (multilocular cyst, solid areas, bilateral lesions, ascites, evidence of metastases on abdominal ultrasound)	No risk estimates; based on clinical, ultrasound and CA125 information; possible to calculate result without computer; online calculators available
Simple rules	Classification as benign, inconclusive, malignant	Classification is based on 10 binary features: five benign features (unilocular cyst, smooth multilocular cyst with largest diameter <100 mm, acoustic shadows, presence of solid areas with largest diameter <7 mm, no vascularisation on colour Doppler) and five malignant features (irregular solid tumour, irregular multilocular solid tumour with largest diameter ≥100 mm, at least four papillary projections, very strong vascularisation on colour Doppler)	No risk estimates; only classification into three groups; based on dichotomised ultrasound features; easy to use without computer; available as smartphone app
LR2	Risk model based on logistic regression	Age (years), presence of acoustic shadows, presence of ascites, presence of papillary projections with blood flow, maximum diameter of largest solid component (mm), irregular internal cyst walls	Risk estimates; based on clinical and ultrasound information; requires computer; available as smartphone app
SRRisk	Risk model based on logistic regression	The 10 binary features used in the simple rules, type of centre (oncology centre *v* other)	Risk estimates; based on dichotomised ultrasound features; developed to add risk estimates to simple rules; risk estimate can be derived by using a simple table for 97% of patients
ADNEX without CA125	Risk model based on multinomial logistic regression	Age (years), maximum diameter of lesion (mm), maximum diameter of largest solid component (mm), number of papillary projections (ordinal), presence of acoustic shadows, presence of ascites, presence of more than 10 cyst locules, and type of centre (oncology centre *v* other)	Risk estimates; the risk of malignancy is subdivided into the risk of four subtypes of malignancy; based on clinical and ultrasound information; subjective predictors were avoided a priori (eg, colour score or irregular cyst walls); requires computer; available as app and as online calculator; available in ultrasound machines from some manufacturers
ADNEX with CA125	Risk model based on multinomial logistic regression	The same variables as in ADNEX without CA125 but with serum CA125 (IU/L) added	Based on clinical, ultrasound, and CA125 information; same comments as for ADNEX without CA125

ADNEX=assessment of different neoplasias in the adnexa; LR2=logistic regression model 2; RMI=risk of malignancy index; SRRisk=simple rules risk model.

### Outcomes

The reference standard describes the nature of the adnexal mass. The primary outcome was classification of tumours as benign or malignant. This classification was based on histology when patients had surgery or subjective assessment at inclusion and during follow-up until 12 (±2) months when surgery was not performed. We considered the outcome as uncertain when not enough information was available to make a reasonable classification of the mass as benign or malignant at inclusion. Table 2 shows detailed classification criteria. Pathologists were blinded to ultrasound predictor variables and model predictions, but might have received information on the subjective assessment by the ultrasound examiner when clinically relevant. Borderline tumours were classified as malignant.

**Table 2:** Definition of tumour outcome based on histology or clinical information

Outcome and scenario	No of tumours
**Benign**	
B1: Surgery, benign histology	2065
B2: No surgery, no spontaneous resolution, last visit ≥10 months, SA at every visit up to 10-14 months was probably benign or certainly benign	911
B3: Spontaneous resolution	465
**Malignant**	
M1: Surgery within 120 days, malignant histology	956*
M2: Surgery after 120 days, malignant histology, SA at every visit up to surgery was probably borderline/malignant or certainly borderline/malignant	18*
M3: No surgery, no spontaneous resolution, last visit ≥10 months, SA at every visit up to 10-14 months was probably borderline/malignant or certainly borderline/malignant	4†
**Uncertain**	
U1: Surgery after 120 days, malignant histology, SA not probably borderline/malignant or certainly borderline/malignant at every visit up to surgery	19*
U2: No surgery, no spontaneous resolution, last visit ≥10 months, SA was uncertain or was inconsistent across visits up to 10-14 months	35
U3: No surgery, no spontaneous resolution, last follow-up visit was before 10 months (owing to death, withdrawal from study, or lost to follow-up)	123
U4: No information after the inclusion visit	309

SA=subjective assessment of ultrasound images.

* In line with previous publications,^10^ 120 days was used as the maximum interval between inclusion and surgery. When surgery was done more than 120 days after inclusion and histology was malignant, the possibility was recognised that the tumour was benign at inclusion but underwent malignant transformation. For these tumours, subjective assessment at inclusion and follow-up scans were relied on to decide whether to categorise the outcome as malignant or as uncertain.

† For these tumours, type of malignancy could not be determined. Type of malignancy was treated as a missing value and imputed (appendix 5).

For a full evaluation of ADNEX, we used a multinomial reference standard describing the adnexal mass at inclusion as benign, borderline malignant, stage I primary ovarian malignancy, stage II-IV primary ovarian malignancy, or secondary metastatic malignancy (secondary outcome).

### Statistical analysis

We followed a prespecified statistical analysis plan for this study. Appendix 5 presents the sample size determination for the IOTA5 study. We had missing values for CA125 and some outcomes were labelled uncertain and therefore missing. We used multiple imputation to address these missing values (appendix 5). The imputations were based on variables used as predictors in the models, and variables that are associated with CA125 or with outcome, or with their missingness. Our primary analysis included patients after multiple imputation of missing values.

We evaluated discrimination between benign and malignant tumours with the area under the receiver operating characteristic curve (AUC) for the risk prediction models and RMI. To account for variability in performance between centres (heterogeneity), we used meta-analysis of centre specific AUCs to obtain the overall AUC for each model. Heterogeneity was quantified using 95% prediction intervals, which indicate which AUC values can be expected when evaluating the model in a new centre.^25^ We used the DeLong method to calculate 95% confidence intervals for the difference in AUC between two models.^26^ For ADNEX, we calculated the AUC for each pair of tumour types.^27^

We calculated sensitivity and specificity for prespecified thresholds for RMI, LR2, SRRisk, and ADNEX. For any threshold, patients with a result at or above the threshold were classified at high risk of malignancy. We compared RMI with other models by calculating sensitivity when fixing specificity at 90%, and specificity when fixing sensitivity at 90%. Overall sensitivity and specificity values were obtained by using a meta-analysis of centre specific results.

We assessed calibration of LR2, SRRisk, and ADNEX by calculating calibration intercept and slope, and used these values to generate centre specific and overall calibration curves. The calibration intercept assesses whether risks are generally overestimated (intercept <0) or underestimated (intercept >0). The calibration slope assesses whether risks are too extreme (slope <1) or too moderate (slope >1).^28^ When too extreme, low estimated risks are underestimated and high risks are overestimated. When too moderate, low risks are overestimated and high risks are underestimated. For RMI, we performed an analogous analysis to estimate the prevalence of malignancy conditional on the RMI value, and constructed centre specific and overall curves. For ADNEX, we assessed calibration for all five predicted outcomes.^29^

We assessed clinical utility by using decision curve analysis for risk thresholds between 5% and 50% to decide which patients should be referred to specialised oncological care. We report overall decision curves based on a meta-analysis of centre specific curves.^30^

We obtained overall AUCs and calibration curves for several prespecified subgroups: actual management (surgery within 120 days without any follow-up scan *v* at least one follow-up scan), management suggested by ultrasound examiner (surgery *v* conservative management with follow-up visits), menopausal status, and type of centre. Overall AUCs and calibration curves were computed for several prespecified sensitivity analyses: an analysis that excludes masses with uncertain outcome (U1-U4 in table 2); an analysis in which the definition of an uncertain outcome is expanded to include groups B2 and M2-M3 in table 2 (all groups in which subjective assessment of ultrasound images was used to classify outcomes as benign or malignant); and an analysis from all 36 centres of patients who underwent surgery within 120 days without any follow-up scan (not restricted to centres with high quality follow-up data). Appendix 5 presents details of the statistical analysis. The analysis was performed by using R version 3.5.1.

### Patient and public involvement

Patients were not involved in the study design, definition of outcome measures, recruiting plans of the study, or interpretation of study results. We discussed the study with KanActief, a cancer rehabilitation patient group at the University Hospitals Leuven (https://www.uzleuven.be/nl/kanactief). 

## Results

**Fig 1:**
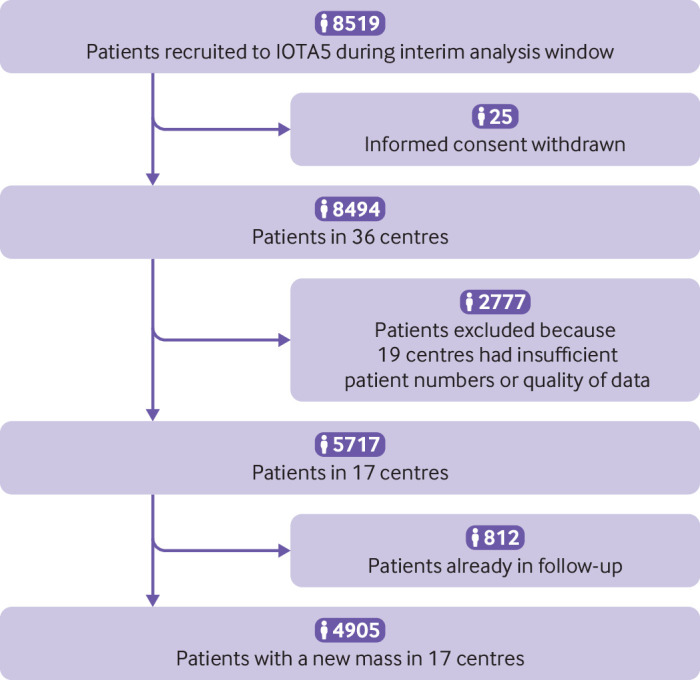
Study flowchart. Criteria for excluding centres were fewer than 50 patients recruited, non-consecutive recruitment, or insufficient quality of follow-up data (appendix 3). Eleven of 20 oncology centres and 8 of 16 non-oncology centres were excluded. Supplementary table 1 gives details of excluded centres. IOTA5=International Ovarian Tumour Analysis phase 5 study

In total, 98 ultrasound examiners at 36 centres recruited 8519 patients into the interim dataset of IOTA5 (supplementary table 1). After we applied the exclusion criteria (appendix 3) and data cleaning, our primary analysis consisted of 4905 patients recruited by 58 ultrasound examiners at 17 centres (fig 1, table 3).

**Table 3** Overview of 17 centres included in primary analysis. Data are number or number (row percentage)

**Table:** 

Centre	No	Outcome*				Actual management†		
		Benign	Malignant	Uncertain		Surgery	Conservative	Unknown
Malmö, Sweden	794	657	78	59		306	464	24
Rome, Italy‡	681	414	173	94		385	225	71
Athens, Greece‡	567	427	68	72		378	120	69
Leuven, Belgium‡	501	356	94	51		212	267	22
Genk, Belgium	406	312	44	50		224	152	30
Milan, Italy‡	367	193	161	13		288	70	9
Stockholm, Sweden‡	363	192	140	31		257	97	9
Monza, Italy‡	267	163	82	22		152	104	11
Cagliari, Italy	166	135	25	6		123	40	3
Katowice, Poland‡	139	110	17	12		45	83	11
Pamplona, Spain‡	111	65	27	19		54	40	17
Trieste, Italy	111	93	16	2		48	63	0
Milan 2, Italy‡	98	53	42	3		58	38	2
London, UK	97	79	5	13		15	78	4
Milan 3, Italy	91	80	1	10		28	55	8
Florence, Italy	85	68	2	15		31	46	8
Nottingham, UK	61	44	3	14		34	16	11
Oncology centres	3094	1973 (64)	804 (26)	317 (10)		1829 (59)	1044 (34)	221 (7)
Other centres	1811	1468 (81)	174 (10)	169 (9)		809 (45)	914 (50)	88 (5)
Total	4905	3441 (70)	978 (20)	486 (10)		2638 (54)	1958 (40)	309 (6)

* Table 2 presents criteria for uncertain outcome. When outcome was uncertain, multiple imputation was used to classify the mass as benign or malignant at inclusion. In one sensitivity analysis a broader definition of uncertain outcome was used.

† Conservative management means that surgery could be performed at any time during follow-up. Unknown management means that no information was available after inclusion scan.

‡ Oncology centre.

The median age of the 4905 patients was 48 years (interquartile range 36-62, range 18-98), and 2151 patients (44%) were postmenopausal (table 4). Information on CA125 was missing in 2620 of the 4905 (53%) patients: 835 of 2579 (32%) missing values when surgery was suggested and 1785 of 2326 (77%) missing values when conservative management was suggested. The outcome was benign for 3441 (70%) patients, malignant for 978 (20%), and uncertain for 486 (10%) patients. The tumours in the current cohort manifested more benign ultrasound features compared with the development datasets of the different models, which were limited to patients who underwent surgery (supplementary table 2).

**Table 4:** Descriptive statistics for patients in primary analysis (n=4905)

Variable	Median (IQR) range, or No (%)
Patient age at recruitment (years)	48 (36-62), 18-98
Postmenopausal	2151 (44)
Gynaecological symptoms during the year preceding inclusion	2565 (52)
Bilateral masses	829 (17)
Tumour type using IOTA terminology	
Unilocular	2140 (44)
Unilocular solid	396 (8)
Multilocular	1011 (21)
Multilocular solid	649 (13)
Solid	689 (14)
Not possible to classify	20 (0.4)
Presence of solid components	1734 (35)
Maximum diameter of lesion (mm)	55 (38-83), 7-751
Colour score of intratumoural flow	
1: no blood flow	2031 (41)
2: minimal blood flow	1336 (27)
3: moderate blood flow	1099 (22)
4: very strong flow	439 (9)
Ultrasound examiner’s presumed diagnosis: any benign diagnosis	3673 (75)
Serous cystadenoma/serous cystadenofibroma	791 (16)
Endometrioma	742 (15)
Simple cyst/para-ovarian or salpingeal cyst	628 (13)
Teratoma	532 (11)
Mucinous cystadenoma/mucinous cystadenofibroma	281 (6)
Fibroma/fibrothecoma	216 (4)
Functional cyst	184 (4)
Hydrosalpinx	156 (3)
Abcess/salpingitis/pelvic inflammatory disease	88 (2)
Inclusion cyst/peritoneal cyst	36 (1)
Benign rare tumour	19 (0.4)
Ultrasound examiner’s presumed diagnosis: any borderline diagnosis	263 (5)
Borderline malignant tumour	218 (4)
Mucinous borderline tumour of intestinal type	39 (1)
Mucinous borderline tumour of endocervical type	6 (0.1)
Ultrasound examiner’s presumed diagnosis: any diagnosis of invasive malignancy	805 (16)
Primary ovarian cancer	598 (12)
Metastasis to the ovary	110 (2)
Malignant rare tumour	97 (2)
Ultrasound examiner’s presumed diagnosis: not possible	164 (3)

IOTA=International Ovarian Tumour Analysis; IQR=interquartile range.

**Fig 2:**
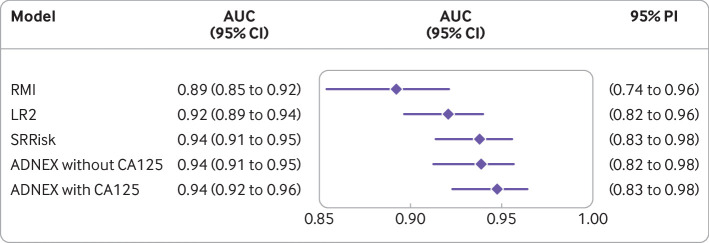
Summary forest plot with overall area under the receiver operating characteristic curve (AUC) for each model. ADNEX=assessment of different neoplasias in the adnexa; LR2=logistic regression model 2; PI=prediction interval; RMI=risk of malignancy index; SRRisk=simple rules risk model

The overall AUC was highest for ADNEX with CA125 (0.94, 95% confidence interval 0.92 to 0.96), ADNEX without CA125 (0.94, 0.91 to 0.95) and SRRisk (0.94, 0.91 to 0.95), and lowest for RMI (0.89, 0.85 to 0.92; fig 2). Differences in AUC between centres (heterogeneity) were largest for RMI, with a 95% prediction interval from 0.74 to 0.96 (supplementary figs 1-5). Supplementary table 3 provides 95% confidence intervals for the difference in AUC between models. At a risk threshold of 10%, ADNEX with CA125 had an overall sensitivity of 91% (95% confidence interval 85% to 95%) and specificity of 85% (81% to 89%). At a threshold of 200, RMI had an overall sensitivity of 60% (54% to 67%) and specificity of 95% (93% to 97%; supplementary tables 4-5). When overall specificity was fixed at 90%, SRRisk had the highest sensitivity (89%) and RMI the lowest (70%), while the sensitivity for ADNEX with CA125 was 87% (table 5). When overall sensitivity was fixed at 90%, ADNEX with CA125 had the highest specificity (87%) and RMI the lowest (69%; table 5). The simple rules model had an overall sensitivity of 90% (86% to 94%) and a specificity of 87% (83% to 91%).

**Table 5:** Sensitivity (at 90% specificity) and specificity (at 90% sensitivity) for all prediction models

Model	Sensitivity at 90% specificity (95% CI)	Specificity at 90% sensitivity (95% CI)
RMI	70.1% (63.5 to 76.0)	69.3% (60.1 to 77.3)
LR2	82.4% (76.3 to 87.1)	81.7% (73.2 to 87.9)
SRRisk	88.5% (83.4 to 92.2)	83.8% (74.2 to 90.3)
ADNEX without CA125	85.2% (78.9 to 89.9)	85.7% (78.5 to 90.8)
ADNEX with CA125	86.5% (80.9 to 90.7)	86.6% (80.8 to 90.9)

ADNEX=assessment of different neoplasias in the adnexa; LR2=logistic regression model 2; RMI=risk of malignancy index; SRRisk=simple rules risk model.

**Fig 3:**
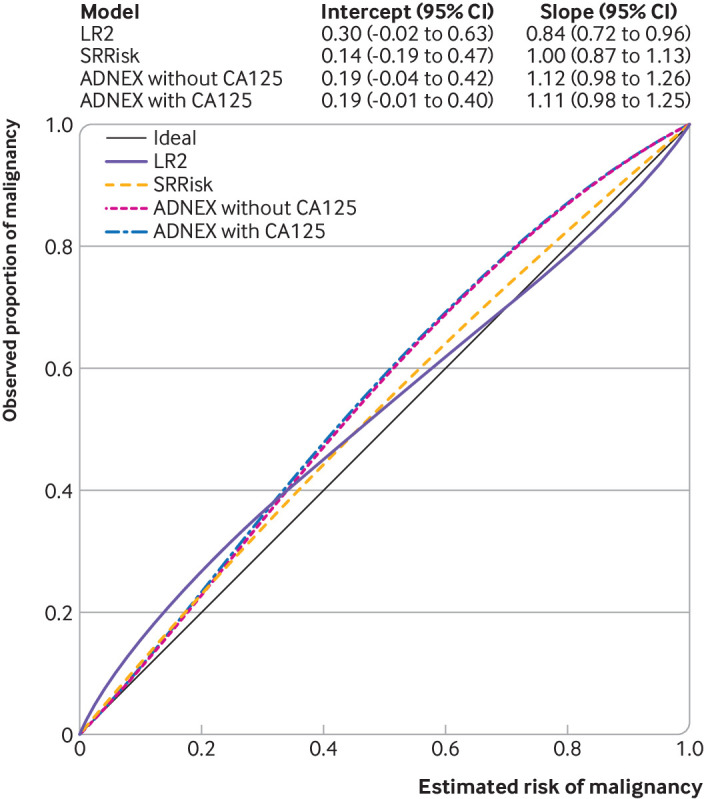
Summary figure with overall calibration curves for risk prediction models. ADNEX=assessment of different neoplasias in the adnexa; intercept=calibration intercept; LR2=logistic regression model 2; RMI=risk of malignancy index; slope=calibration slope; SRRisk=simple rules risk model

ADNEX with CA125 had an overall calibration intercept of 0.19 (95% confidence interval −0.01 to 0.40) and a slope of 1.11 (0.98 to 1.25). Risk estimates were slightly underestimated (fig 3). Calibration of SRRisk was marginally better and that of LR2 was poorer. We observed heterogeneity between centres for calibration for all models, with least heterogeneity for ADNEX with CA125 (supplementary figs 6-10). The overall calibration curve for RMI (supplementary fig 11) indicated that the commonly used threshold of 200 corresponded to a risk of malignancy of 45-50% on average. Supplementary figs 12-16 present histograms of the predictions of RMI and the risk prediction models.

**Fig 4:**
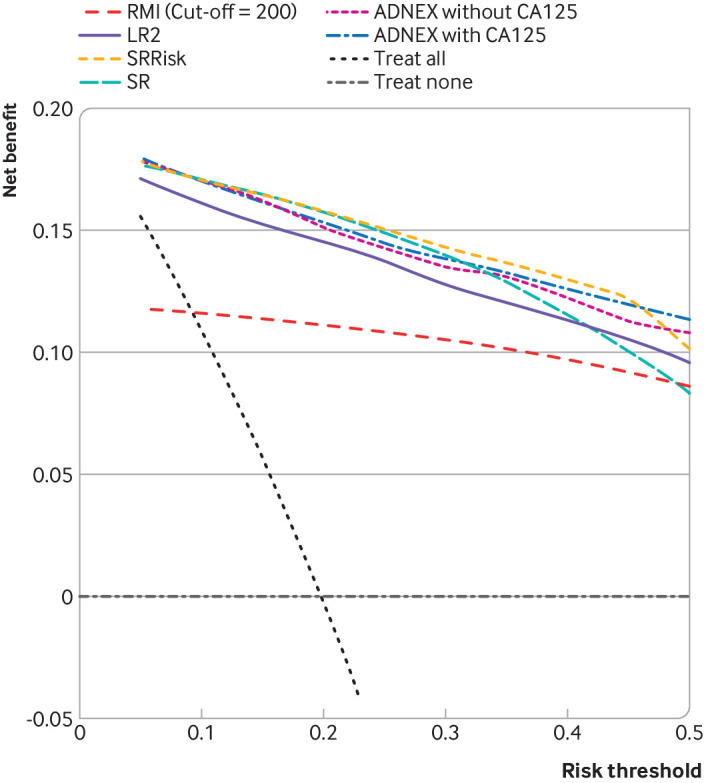
Overall decision curves for risk prediction models and RMI. Higher net benefit implies higher clinical utility (the higher the curve, the better the clinical utility at the chosen risk threshold).^18^ ^30^ ADNEX=assessment of different neoplasias in the adnexa; LR2=logistic regression model 2; RMI=risk of malignancy index; SRRisk=simple rules risk model

SRRisk and ADNEX with CA125 had the best overall utility to select patients for referral to a gynaecological oncology centre (fig 4). RMI at a threshold of 200 had the lowest clinical utility of all the models.

For the ADNEX model with CA125, distinguishing between borderline and stage I primary ovarian malignancy (AUC 0.77), between stage I primary ovarian malignancy and secondary metastatic cancer (AUC 0.75), and between stage II-IV primary ovarian malignancy and secondary metastatic cancer (AUC 0.78) was the most difficult (supplementary table 6). AUCs ranged from 0.90 to 0.98 when distinguishing between benign tumours and malignant subtypes. ADNEX without CA125 mainly affected discrimination between stage II-IV and stage I primary ovarian malignancy (AUC was 0.81 when CA125 was included *v* 0.72 when CA125 was not included), and between stage II-IV primary ovarian malignancy and secondary metastatic malignancy (AUC 0.78 *v* 0.66). Calibration of the estimated risks for the five tumour subtypes was good for ADNEX irrespective of whether or not CA125 was included as a predictor (supplementary figs 17-18).

In every subgroup, RMI had the lowest overall AUC and ADNEX with CA125 the highest overall AUC (fig 5, supplementary table 7). Among the 1958 patients with at least one follow-up scan, the overall AUC was 0.76 for RMI and ranged from 0.87 to 0.89 for the IOTA models. Because of the low malignancy rate (2%) in this subgroup, the confidence interval around the AUC was wide. Calibration analysis indicated that the risk of malignancy was overestimated in this subgroup (supplementary figs 19-34). The results obtained in the sensitivity analyses were similar to those in the primary analysis (supplementary figs 35-43).

**Fig 5:**
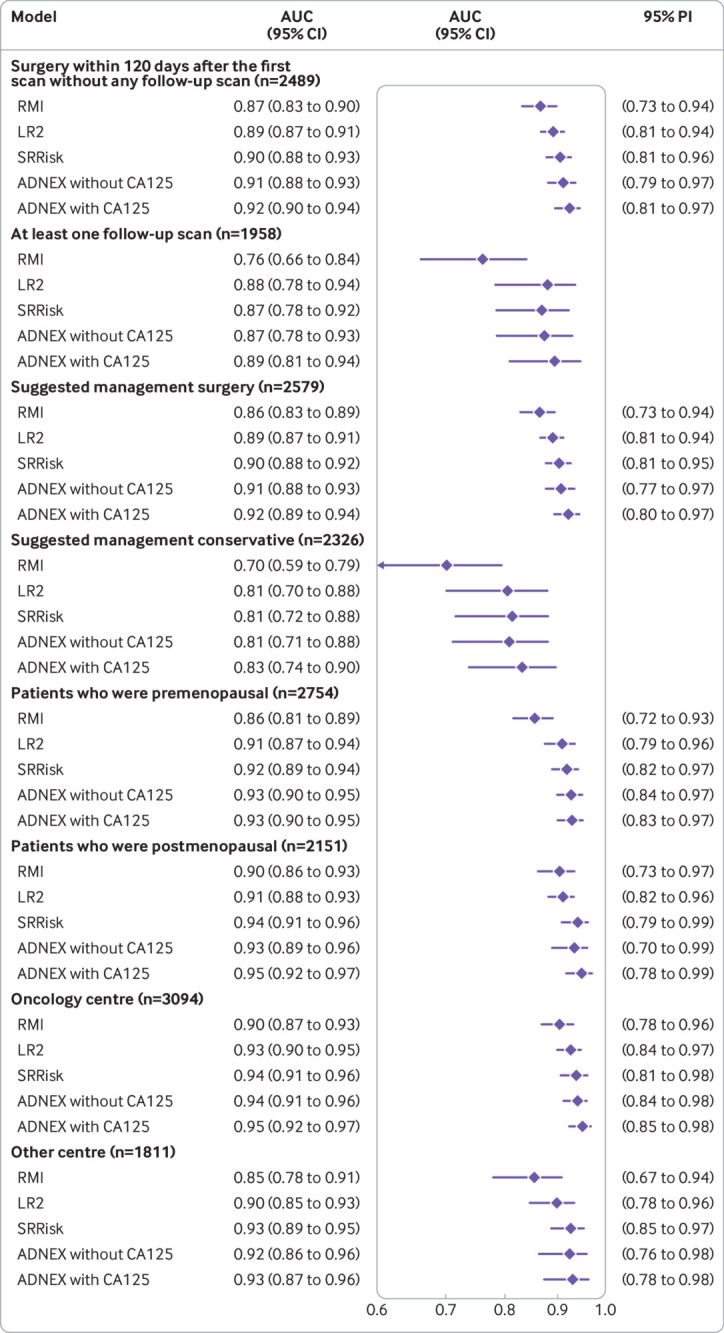
Summary forest plots of overall area under the receiver operating characteristic curve (AUC) for prespecified subgroups. Prediction intervals could not be calculated for two subgroups because the number of malignant outcomes for each centre was too small for meta-analysis to be possible. ADNEX=assessment of different neoplasias in the adnexa; LR2=logistic regression model 2; PI=prediction interval; RMI=risk of malignancy index; SRRisk=simple rules risk model

## Discussion

### Principal findings

This study is a comprehensive evaluation of RMI and IOTA models when applied to all patients presenting with an adnexal mass, irrespective of whether they received surgical or conservative management. ADNEX with CA125, ADNEX without CA125, and SRRisk were the best models to distinguish between benign and malignant adnexal masses. These models were reasonably well calibrated overall, and had the highest clinical utility. RMI had the lowest AUC and clinical utility. Performance varied between centres for all models, but it varied most for RMI. In every prespecified subgroup, ADNEX with CA125 had the highest AUC and RMI the lowest AUC.

All models, in particular RMI, had poorer discriminative ability in patients who were managed conservatively than in those who underwent surgery. This difference could be because masses managed conservatively are more homogenous than those removed surgically. Most masses managed conservatively probably manifested clearly benign ultrasound signs, few were malignant, and most malignancies managed conservatively were borderline tumours, with ultrasound features that could be confused with those of benign tumours.^31^ ^32^ All models overestimated the risk of malignancy in patients who were managed conservatively, which was expected because none of the models was developed for this population.

### Strengths and limitations of the study

Our study has several strengths. We included patients irrespective of whether they were managed surgically or conservatively, and the large sample size allowed us to evaluate models in clinically relevant subgroups. Additionally we recruited from many centres in different countries, used a large number of ultrasound examiners, and implemented a rigorous prospective protocol with agreed ultrasound terms, definitions, and measurement technique.^22^ Finally, we evaluated the calibration and clinical utility of all the models.

The first limitation is that several centres had to be excluded because of non-consecutive recruitment or insufficient quality of follow-up data. However, a similar proportion of oncology centres (11/20) and non-oncology centres (8/16) were excluded. The second, inevitable limitation is that our reference standard is based on two different methods: histology or results of clinical and ultrasound follow-up.^33^ Follow-up information is probably less accurate than histology when assigning the outcome; for some patients the outcome was partly based on subjective assessment at inclusion. We limited the risk of bias in our primary analysis by using multiple imputation to assign an outcome when clinical and ultrasound information was insufficient or inconsistent (n=486, 10% of all cases). Excluding uncertain outcomes might have induced bias because the assumption could be made that patients who do not undergo surgery without delay are more likely to have a benign tumour. Our two sensitivity analyses, one excluding all uncertain cases (U1-U4 in table 2) and one using imputation to assign an outcome when a broader definition of uncertain outcome was applied (B2, M2-M3, and U1-U4 in table 2; n=1419, 31% of all cases), showed similar results to those in our primary analysis. The third limitation is that CA125 values were missing in a substantial number of patients. We addressed the missing values using multiple imputation.^34^ Imputing missing values multiple times acknowledges that we are uncertain about the true value. The observation that the ranking of models in terms of AUC was the same in women who underwent surgery (low proportion of missing CA125 values) and in women who received conservative management (high proportion of missing CA125 values) suggests that our results are robust.

### Comparison with other studies

Two small single centre studies have evaluated IOTA models on all patients irrespective of management. Nunes and colleagues (n=489) evaluated sensitivity and specificity of LR2 using a one risk cut-off point.^19^ Pereira and colleagues (n=170) evaluated the clinical utility of simple rules and SRRisk.^20^ Other published validation studies were limited to patients who underwent surgery. These studies showed that IOTA models distinguished better between benign and malignant adnexal masses than RMI, and that ADNEX might be the best performing model.^12^ ^13^ ^14^ ^16^ ^35^ ^36^ ^37^ ^38^ The studies also showed that SRRisk and ADNEX had good overall calibration (the authors did not report centre specific results) and better clinical utility than other models, including RMI, to refer patients to an oncology centre.^10^ ^11^ ^17^ ^18^ To distinguish between benign and malignant masses in patients who underwent surgery, the following AUCs have been reported for ADNEX with CA125: 0.94 in the original study, between 0.91 and 0.97 in external validation studies, and 0.92 in the subgroup that underwent surgery in the current study.^10^ ^17^ ^37^ ^38^ ^39^ ^40^ ^41^ ^42^

### Implications for practice

In our study, the ADNEX models and SRRisk were the best performing models and were similar in terms of discrimination, calibration, and clinical utility. However, we would recommend ADNEX rather than SRRisk for several reasons. Firstly, ADNEX uses only simple and robust ultrasound variables, and so less experience should be needed for correct use of this model compared with SRRisk. Secondly, ADNEX estimates the likelihood of five different tumour types. This information could help when deciding which investigations to perform (investigations would differ if a metastasis is suspected rather than primary ovarian cancer), which surgical strategy to choose (fertility sparing surgery could be considered if a borderline tumour is suspected), and how long the waiting time should be for the operation. The likelihood of different tumour types can also help when deciding on the appropriate skills of the surgeon and estimating the duration of surgery. Other models do not provide this information.

Because CA125 results are often not available when the patient is examined with ultrasound, ADNEX without CA125 can be used as a first step to distinguish between benign and malignant tumours during scanning. If ADNEX without CA125 yields a high risk of malignancy, blood sampling with analysis of CA125 can be arranged so that the most likely type of malignancy can be estimated by using ADNEX with CA125. Including CA125 in ADNEX improves discrimination between the malignant tumour types, but has little effect on the discrimination between benign and malignant masses. For application in clinical practice, ADNEX is available as an app for iOS or Android and as a web calculator (https://iotagroup.org/iota-models-software/adnex-risk-model). Some ultrasound machines have built-in functionalities that allow ADNEX to be used while the patient is being scanned.

A prerequisite for safe use of the IOTA models is basic ultrasound skills. Additionally, ultrasound examiners must have obtained the IOTA certificate (https://www.iotagroup.org/certified-members) to show that they are able to correctly use the IOTA terminology and measurement technique (http://www.iota.education).^22^ ^43^ For safe use of ADNEX, examiners need to use the IOTA definitions for solid component, papillary projection, acoustic shadowing, and ascites.

Because of the consecutive recruitment of patients into our study, the substantial sample size, and the large number of ultrasound examiners and participating centres in different countries, our results could be generalisable to other patient populations. However, for all models heterogeneity existed between centres in terms of performance, in particular calibration. The heterogeneity can probably be explained by differences in tumour characteristics that are not captured by the predictors, and in tumour mix (that is, the relative proportion of benign, borderline, primary invasive malignancies, and metastases). Further research should focus on explaining and reducing this heterogeneity. The performance of the ADNEX model could be improved by updating the model by using data from patients who are conservatively managed and those who have had surgery.

### Conclusions and policy implications

Our study has shown that ADNEX with or without CA125 and SRRisk are the best models for distinguishing between benign and malignant tumours in patients presenting with an adnexal mass. Because ADNEX is preferable to SRRisk for practical reasons, the model should be recommended for characterising ovarian tumours. The next step is to achieve consensus on the risk thresholds to be used when deciding whether patients with an adnexal tumour should receive conservative management, surgery in a local centre, or be referred to a gynaecological oncology centre for further evaluation.

**What is already known on this topic**

Methods are needed to predict malignancy in ovarian tumours so that optimal management can be selected, such as watch and wait, surgery in a local hospital, or treatment in a gynaecological oncology centre
Existing prediction models are based only on data from patients who have undergone surgery
Model validation has mostly used data from patients who have had surgery and has rarely included assessment of calibration or clinical utility

**What this study adds**

Assessment of different neoplasias in the adnexa (ADNEX) with or without CA125 and simple rules risk model (SRRisk) are the best models for distinguishing between benign and malignant adnexal tumours
ADNEX with CA125 performed best when distinguishing between benign and malignant tumours in all subgroups (including patients managed conservatively)
ADNEX has practical advantages over SRRisk, including the ability to estimate risk of malignant subtypes

## Web extra

Extra material supplied by authors

Web appendix: Supplementary material

## Notes

Contributors: BVC, LV, ACT, TB, and DT conceived and designed the study. LV, WF, CL, ACT, PS, CVH, ED, RF, EE, DF, MJK, VC, JLA, FPGL, FB, MEC, SG, ND, LJ, LS, DF, ACz, JK, ACo, GS, IV, TB, and DT enrolled patients and acquired data. BVC, WF, CL, JC, and DT were involved in data cleaning. BVC, LV, WF, CL, JC, LW, and DT wrote the Statistical Analysis Plan. BVC and JC analysed the data, with support from LW. BVC, LV, WF, CL, JC, LW, TB, and DT were involved in data interpretation. BVC, LV, WF, CL, JC, and DT wrote the first draft of the manuscript. All authors critically reviewed and revised the draft. All authors approved the final version of the manuscript for submission. BVC and LV contributed equally. BVC, LV, and DT are the guarantors. The corresponding author attests that all listed authors meet authorship criteria and that no others meeting the criteria have been omitted.

Ethical approval: Approval was obtained from the ethics committee of the University Hospitals Leuven (Ethische Commissie Onderzoek UZ/KU Leuven, https://www.uzleuven.be/nl/ethischecommissie/onderzoek) as the coordinating centre (B32220095331/S51375) and the local ethics committee of each contributing centre.

Data sharing: Follow-up data collection for the IOTA5 study is still ongoing. When the study has closed, and results have been published, the corresponding author may share data upon reasonable request (dirk.timmerman@uzleuven.be). R code used for the statistical analysis is available on GitHub (https://github.com/benvancalster/IOTA5modelvalidation2020).

The lead authors affirm that the manuscript is an honest, accurate, and transparent account of the study being reported; that no important aspects of the study have been omitted; and that any discrepancies from the study as planned have been explained.

Dissemination to participants and related patient and public communities: We plan to disseminate and discuss the results with patient groups and societies for gynaecology and gynaecological oncology, and through both media organisations and social media. This will be highly valuable when it comes to implementation of models into clinical practice.
