# Supplementary material for: Validation of models to diagnose ovarian cancer in patients managed surgically or conservatively: multicentre cohort study
Source: BMJ. 2020 Jul 30;370:m2614. doi: 10.1136/bmj.m2614 (PMC7391073; doi:10.1136/bmj.m2614)
Supplement: Supplementary file 1 — Web appendix: Supplementary material [file calb054803.ww.pdf]

Validation of models to diagnose cancer in patients managed surgically or conservatively for an ovarian tumour: a multicentre cohort study.

Ben Van Calster, Lil Valentin, Wouter Froyman, Chiara Landolfo, Jolien Ceusters, Antonia C Testa, Laure Wynants, Povilas Sladkevicius, Caroline Van Holsbeke, Ekaterini Domali, Robert Fruscio, Elisabeth Epstein, Dorella Franchi, Marek J Kudla, Valentina Chiappa, Juan L Alcazar, Francesco P G Leone, Francesca Buonomo, Maria Elisabetta Coccia, Stefano Guerriero, Nandita Deo, Ligita Jokubkiene, Luca Savelli, Daniela Fischerova, Artur Czekierdowski, Jeroen Kaijser, An Coosemans, Giovanni Scambia, Ignace Vergote, Tom Bourne, Dirk Timmerman

Supplementary material

Table of Contents

Supplementary Table 1	4
Supplementary Table 2	5
Supplementary Figure 1	6
Supplementary Figure 2	7
Supplementary Figure 3	8
Supplementary Figure 4	9
Supplementary Figure 5	10
Supplementary Table 3	11
Supplementary Table 4	12
Supplementary Table 5	13
Supplementary Figure 6	14
Supplementary Figure 7	15
Supplementary Figure 8	16
Supplementary Figure 9	17
Supplementary Figure 10	18
Supplementary Figure 11	19
Supplementary Figure 12	20
Supplementary Figure 13	21
Supplementary Figure 14	22
Supplementary Figure 15	23
Supplementary Figure 16	24
Supplementary Table 6	25
Supplementary Figure 17	26
Supplementary Figure 18	27
Supplementary Table 7	28
Supplementary Figure 19	29
Supplementary Figure 20	30
Supplementary Figure 21	31
Supplementary Figure 22	32
Supplementary Figure 23	33
Supplementary Figure 24	34
Supplementary Figure 25	35
Supplementary Figure 26	36
Supplementary Figure 27	37
Supplementary Figure 28	38

Supplementary Figure 29 39
Supplementary Figure 30 40
Supplementary Figure 31 41
Supplementary Figure 32 42
Supplementary Figure 33 43
Supplementary Figure 34 44
Supplementary Figure 35 45
Supplementary Figure 36 46
Supplementary Figure 37 47
Supplementary Figure 38 48
Supplementary Figure 39 49
Supplementary Figure 40 50
Supplementary Figure 41 51
Supplementary Figure 42 52
Supplementary Figure 43 53
Appendix 1 – IOTA5 protocol 54
Appendix 2 – Telephone survey 70
Appendix 3 – Details on the exclusion of centres 77
Appendix 4 – Prediction models 78
Appendix 5 – Details on imputation and statistical analysis 83
References for the supplementary material 89

Supplementary Table 1. Overview of patients and centres that participated in the interim analysis of IOTA5.

Centre	Principal investigator	n all	n new	Missing CA125, %	Outcome[a]			Actual management[b]			Decision[c]
					Benign	Malignant	Uncertain	Surgery	Conservative	Unknown	
Malmö, Sweden	Lil Valentin, MD	973	794	74%	657	78	59	306	464	24	INCLUDED
Bologna, Italy[d]	Luca Savelli, MD	917	790	73%	419	104	267	278	346	166	Excluded
Rome, Italy[d]	Antonia Testa, MD	721	681	62%	414	173	94	385	225	71	INCLUDED
Athens, Greece[d]	Ekaterini Domali, MD	572	567	49%	427	68	72	378	120	69	INCLUDED
Leuven, Belgium[d]	Dirk Timmerman, MD	602	501	67%	356	94	51	212	267	22	INCLUDED
Genk, Belgium	Caroline Van Holsbeke, MD	446	406	75%	312	44	50	224	152	30	INCLUDED
Milan, Italy[d]	Dorella Franchi, MD	418	367	11%	193	161	13	288	70	9	INCLUDED
Stockholm, Sweden[d]	Elisabeth Epstein, MD	454	363	21%	192	140	31	257	97	9	INCLUDED
Lublin, Poland[d]	Artur Czekierdowski, MD	363	361	27%	216	35	110	121	151	89	Excluded
Prague, Czech Republic[d]	Daniela Fischerova, MD	347	316	38%	72	71	173	89	76	151	Excluded
Monza, Italy[d]	Robert Fruscio, MD	430	267	31%	163	82	22	152	104	11	INCLUDED
Cagliari, Italy	Stefano Guerriero, MD	182	166	16%	135	25	6	123	40	3	INCLUDED
Lisbon, Portugal[d]	Maria José dos Santos Bernardo, MD	146	146	25%	142	0	4	14	132	0	Excluded
Bari, Italy[d]	Doriana Scardigno, MD	143	143	10%	88	25	30	109	9	25	Excluded
Katowice, Poland[d]	Marek Kudla, MD	139	139	73%	110	17	12	45	83	11	INCLUDED
Udine, Italy[d]	Alberto Rossi, MD	117	117	0%	87	29	1	116	0	1	Excluded
Pamplona, Spain[d]	Juan Luis Alcázar, MD	154	111	70%	65	27	19	54	40	17	INCLUDED
Trieste, Italy	Francesca Buonomo, MD	112	111	23%	93	16	2	48	63	0	INCLUDED
Milan 2, Italy[d]	Valentina Chiappa, MD	143	98	18%	53	42	3	58	38	2	INCLUDED
London, UK	Tom Bourne, MD	100	97	55%	79	5	13	15	78	4	INCLUDED
Krakow, Poland[d]	Anna Knafel, MD	175	96	65%	46	41	9	83	4	9	Excluded
Milan 3, Italy	Francesco Paolo Leone, MD	114	91	92%	80	1	10	28	55	8	INCLUDED
Florence, Italy	Maria Elisabetta Coccia, MD	95	85	94%	68	2	15	31	46	8	INCLUDED
Milan 4, Italy	Chiara Lanzani, MD	64	64	0%	36	1	27	16	29	19	Excluded
Nottingham, UK	Nandita Deo, MD	62	61	49%	44	3	14	34	16	11	INCLUDED
Beijing, China[d]	Jing Zhang, MD	60	60	2%	26	16	18	39	4	17	Excluded
Cairo, Egypt	Mona Aboulghar, MD	64	59	75%	38	7	14	29	17	13	Excluded
Lisbon 2, Portugal[d]	Fatima Alves, MD	49	48	15%	32	4	12	22	18	8	Excluded
Tienen, Belgium	Thierry Van den Bosch, MD	60	46	85%	4	0	42	1	10	35	Excluded
Cremona, Italy	Paola Pollastri, MD	47	40	60%	31	2	7	24	16	0	Excluded
Catania, Italy[d]	Maria Concetta Blanco, MD	28	28	61%	14	3	11	12	8	8	Excluded
Paris, France	Perrine Capmas, MD	28	28	57%	14	6	8	20	0	8	Excluded
Aarschot, Belgium	Thierry Van den Bosch, MD	45	26	100%	5	0	21	1	8	17	Excluded
Tampa, United States	Lauri Hochberg, MD	60	23	74%	17	0	6	0	19	4	Excluded
Maurepas, France	Ulrike Metzger, MD	48	17	88%	11	0	6	3	9	5	Excluded
Vienna, Austria[d]	Samir Helmy, MD	16	16	69%	12	0	4	3	12	1	Excluded
Total		8494[e]	7329	51%	4751	1322	1256	3618	2826	885	

[a] Criteria for uncertain outcome are shown in Table 1.

[b] Surgery, surgery without any follow-up scan before surgery; Conservative, at least one follow-up scan. Unknown management means that we have no information after the inclusion scan.

[c] Decision: whether the centre was included or excluded from the primary analysis, see Appendix 3 for details and Froyman et al (2019).[1]

[d] These centres are oncology centres

[e] Out of 8519 patients recruited into the study 25 withdrew consent.

Supplementary Table 2. Descriptive statistics of predictor variables in all development datasets and in the current dataset.

Variable	Model[a]	RMI development (n=143, 1 centre)[2]	LR2/SR development (n=1066, 9 centres)[3,4]	SRRisk development (n=4848, 22 centres)[5,b]	ADNEX development (n=5914, 24 centres),[6]	Current data (n=4905, 17 centres)
Malignant outcome, %		29	25	34	33	21
Age (years), mean	LR2, ADNEX	52	47	48	48	49
Postmenopausal, %	RMI	58	41	41	41	44
CA125 (U/ml), mean	RMI, ADNEX	48	305	363	352	318
CA125 (U/ml), median	RMI, ADNEX	-	23	33	30	25
CA125, % missing	RMI, ADNEX	0	24	32	31	53
Max. diameter of lesion (mm), median	ADNEX	-	68	69	69	55
Max. diameter of solid area (mm), median	LR2	-	5	8	7	0
Proportion solid tissue, median	ADNEX	-	0.06	0.13	0.11	0
Presence of solid areas, %	RMI	-	52	53	53	35
Irregular internal cyst walls, %	LR2, SR, SRRisk	-	45	39	40	31
Acoustic shadows, %	LR2, SR, SRRisk, ADNEX	-	10	13	13	15
Ascites, %	RMI, LR2, SR, SRRisk, ADNEX	-	13	12	12	6
Number of papillations, mean	ADNEX	-	0.60	0.41	0.45	0.25
Papillations with blood flow, %	LR2	-	14	9	10	6
Bilateral	RMI	-	20	19	19	17
Multilocular cyst, %	RMI	-	45	37	39	34
>10 locules, %	ADNEX	-	9	8	8	8
Abdominal metastases, %	RMI	-	-	14	14	7
Unilocular cyst, %	SR, SRRisk	-	29	30	30	44
Solid areas, but smaller than 7mm, %	SR, SRRisk	-	4	2	2	2
Smooth multilocular cyst <100mm, %	SR, SRRisk	-	11	10	10	13
No blood flow (color score 1), %	SR, SRRisk	-	22	29	28	41
Irregular solid tumor, %	SR, SRRisk	-	6	6	6	4
At least 4 papillations, %	SR, SRRisk	-	9	5	6	3
Irregular multilocular-solid cyst ≥100mm, %	SR, SRRisk	-	9	8	8	4
Very strong blood flow (color score 4), %	SR, SRRisk	-	14	12	12	9

Max, maximum; RMI, risk of malignancy index; LR2, logistic regression model 2; SR, Simple Rules; SRRisk, Simple Rules risk model; ADNEX, Assessment of Different NEoplasias in the adneXa.
The superscript numbers in the column names refer to reference numbers for these studies (cf p 89).
[a] In this column, we do not distinguish between ADNEX with and without CA125. ADNEX with CA125 uses the same predictors as ADNEX without CA125, but with the addition of CA125.
[b] The development dataset of SRRisk forms part of that of ADNEX, therefore results are similar for these two development sets. The differences between the development studies and the current study are mainly explained by patients managed conservatively being included in the current study, while only patients who underwent surgery were included in the development studies.

Supplementary Figure 1. Forest plot with centre-specific areas under the receiver operating characteristic curve (AUC) of the Risk of Malignancy Index (RMI). CI, confidence interval. ”Other” includes the following small non-oncology centres with low prevalence of malignancy: London and Nottingham from the UK, and Milan 3 and Florence from Italy.

Centre	AUC (95% CI)	N
Malmö (Sweden)	0.82 (0.76 to 0.86)	794
Rome (Italy)	0.89 (0.86 to 0.92)	681
Athens (Greece)	0.84 (0.78 to 0.88)	567
Leuven (Belgium)	0.94 (0.90 to 0.96)	501
Genk (Belgium)	0.86 (0.79 to 0.91)	406
Milan 1 (Italy)	0.92 (0.89 to 0.95)	367
Stockholm (Sweden)	0.83 (0.78 to 0.87)	363
Monza (Italy)	0.90 (0.85 to 0.94)	267
Cagliari (Italy)	0.93 (0.85 to 0.97)	166
Katowice (Poland)	0.93 (0.82 to 0.98)	139
Pamplona (Spain)	0.97 (0.88 to 0.99)	111
Trieste (Italy)	0.89 (0.76 to 0.95)	111
Milan 2 (Italy)	0.93 (0.85 to 0.97)	98
Other	0.74 (0.57 to 0.86)	334
**Meta-analysis**		
**AUC (95% CI)**	**0.89 (0.85 to 0.92)**	**4905**
**95% Prediction interval**	**(0.74 to 0.96)**	

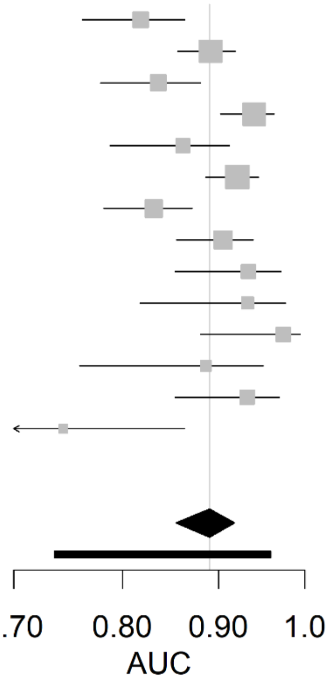

Supplementary Figure 2. Forest plot with centre-specific areas under the receiver operating characteristic curve (AUC) of logistic regression model 2 (LR2). CI, confidence interval. ”Other” includes the following small non-oncology centres with low prevalence of malignancy: London and Nottingham from the UK, and Milan 3 and Florence from Italy.

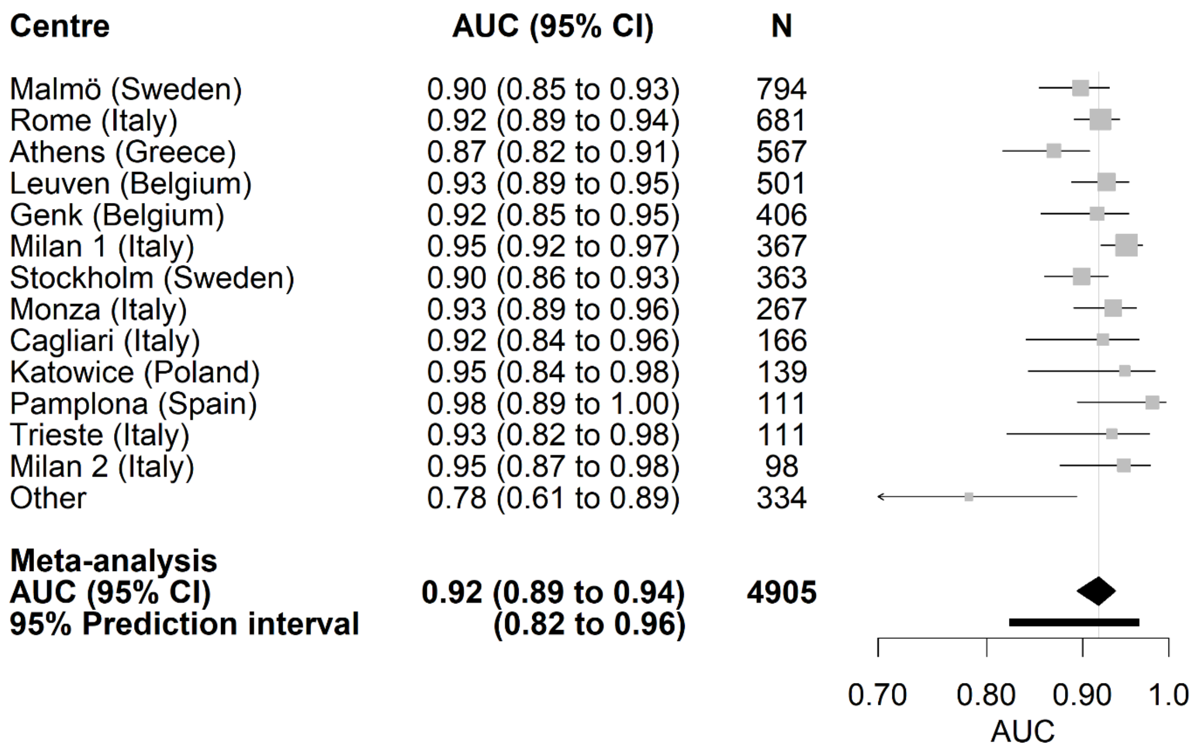

Supplementary Figure 3. Forest plot with centre-specific areas under the receiver operating characteristic curve (AUC) of Simple Rules risk model (SRRisk). CI, confidence interval. ”Other” includes the following small non-oncology centres with low prevalence of malignancy: London and Nottingham from the UK, and Milan 3 and Florence from Italy.

Centre	AUC (95% CI)	N
Malmö (Sweden)	0.90 (0.85 to 0.93)	794
Rome (Italy)	0.94 (0.91 to 0.96)	681
Athens (Greece)	0.84 (0.78 to 0.88)	567
Leuven (Belgium)	0.96 (0.93 to 0.98)	501
Genk (Belgium)	0.96 (0.90 to 0.98)	406
Milan 1 (Italy)	0.97 (0.94 to 0.98)	367
Stockholm (Sweden)	0.92 (0.89 to 0.95)	363
Monza (Italy)	0.91 (0.86 to 0.94)	267
Cagliari (Italy)	0.93 (0.85 to 0.97)	166
Katowice (Poland)	0.97 (0.86 to 0.99)	139
Pamplona (Spain)	0.99 (0.88 to 1.00)	111
Trieste (Italy)	0.96 (0.86 to 0.99)	111
Milan 2 (Italy)	0.96 (0.89 to 0.99)	98
Other	0.90 (0.76 to 0.96)	334
**Meta-analysis**		
**AUC (95% CI)**	**0.94 (0.91 to 0.95)**	**4905**
**95% Prediction interval**	**(0.83 to 0.98)**	

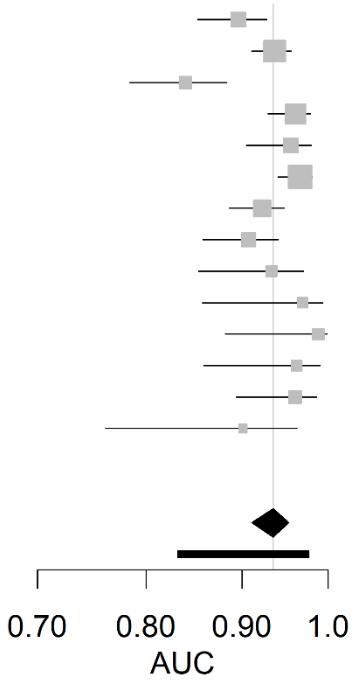

Supplementary Figure 4. Forest plot with centre-specific areas under the receiver operating characteristic curve (AUC) of Assessment of Different NEoplasias in the adneXa (ADNEX) without CA125. CI, confidence interval. "Other" includes the following small non-oncology centres with low prevalence of malignancy: London and Nottingham from the UK, and Milan 3 and Florence from Italy.

Centre	AUC (95% CI)	N
Malmö (Sweden)	0.92 (0.88 to 0.95)	794
Rome (Italy)	0.95 (0.92 to 0.96)	681
Athens (Greece)	0.87 (0.82 to 0.91)	567
Leuven (Belgium)	0.96 (0.93 to 0.98)	501
Genk (Belgium)	0.95 (0.90 to 0.98)	406
Milan 1 (Italy)	0.97 (0.94 to 0.98)	367
Stockholm (Sweden)	0.92 (0.89 to 0.95)	363
Monza (Italy)	0.93 (0.89 to 0.96)	267
Cagliari (Italy)	0.95 (0.88 to 0.98)	166
Katowice (Poland)	0.93 (0.80 to 0.98)	139
Pamplona (Spain)	0.99 (0.87 to 1.00)	111
Trieste (Italy)	0.95 (0.84 to 0.99)	111
Milan 2 (Italy)	0.96 (0.89 to 0.99)	98
Other	0.79 (0.62 to 0.89)	334
**Meta-analysis**		
**AUC (95% CI)**	**0.94 (0.91 to 0.95)**	**4905**
**95% Prediction interval**	**(0.82 to 0.98)**	

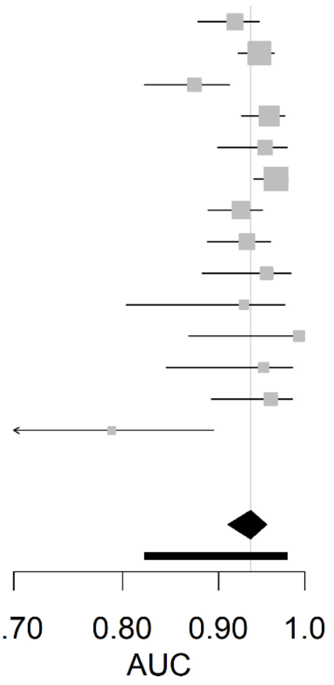

Supplementary Figure 5. Forest plot with centre-specific areas under the receiver operating characteristic curve (AUC) of Assessment of Different NEoplasias in the adneXa (ADNEX) with CA125. CI, confidence interval. ”Other” includes the following small non-oncology centres with low prevalence of malignancy: London and Nottingham from the UK, and Milan 3 and Florence from Italy.

Centre	AUC (95% CI)	N
Malmö (Sweden)	0.93 (0.89 to 0.95)	794
Rome (Italy)	0.96 (0.93 to 0.97)	681
Athens (Greece)	0.89 (0.84 to 0.92)	567
Leuven (Belgium)	0.96 (0.93 to 0.98)	501
Genk (Belgium)	0.95 (0.89 to 0.98)	406
Milan 1 (Italy)	0.97 (0.95 to 0.99)	367
Stockholm (Sweden)	0.93 (0.89 to 0.95)	363
Monza (Italy)	0.95 (0.90 to 0.97)	267
Cagliari (Italy)	0.96 (0.89 to 0.99)	166
Katowice (Poland)	0.94 (0.82 to 0.98)	139
Pamplona (Spain)	1.00 (0.83 to 1.00)	111
Trieste (Italy)	0.96 (0.85 to 0.99)	111
Milan 2 (Italy)	0.97 (0.91 to 0.99)	98
Other	0.81 (0.63 to 0.91)	334
**Meta-analysis**		
**AUC (95% CI)**	**0.94 (0.92 to 0.96)**	**4905**
**95% Prediction interval**	**(0.83 to 0.98)**	

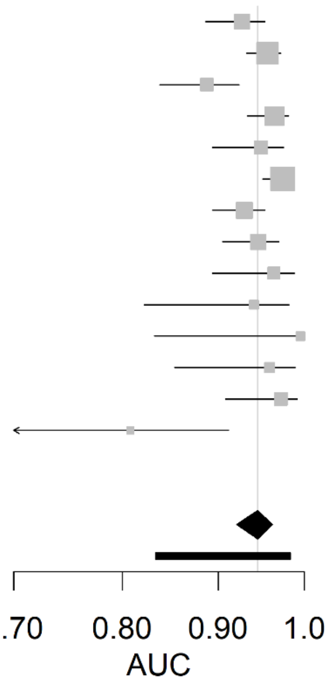

Supplementary Table 3. 95% confidence intervals of the overall difference in AUC between models for the primary analysis and for all subgroups. Positive values favor the first model.

Model comparison	Primary analysis
ADNEX with CA125 vs ADNEX without CA125	0.004 to 0.011
ADNEX with CA125 vs SRRisk	0.006 to 0.019
ADNEX with CA125 vs LR2	0.020 to 0.034
ADNEX with CA125 vs RMI	0.037 to 0.067
ADNEX without CA125 vs SRRisk	-0.001 to 0.011
ADNEX without CA125 vs LR2	0.013 to 0.025
ADNEX without CA125 vs RMI	0.028 to 0.060
SRRisk vs LR2	0.001 to 0.022
SRRisk vs RMI	0.022 to 0.059
LR2 vs RMI	0.011 to 0.042

Model comparison	Surgery within 120 days, no follow-up scan	At least 1 follow-up scan
ADNEX with CA125 vs ADNEX without CA125	0.005 to 0.013	0.015 to 0.057
ADNEX with CA125 vs SRRisk	0.012 to 0.029	-0.039 to 0.091
ADNEX with CA125 vs LR2	0.023 to 0.040	-0.056 to 0.086
ADNEX with CA125 vs RMI	0.037 to 0.072	0.053 to 0.214
ADNEX without CA125 vs SRRisk	0.003 to 0.019	-0.056 to 0.065
ADNEX without CA125 vs LR2	0.011 to 0.028	-0.079 to 0.066
ADNEX without CA125 vs RMI	0.024 to 0.065	0.022 to 0.203
SRRisk vs LR2	-0.003 to 0.021	-0.089 to 0.067
SRRisk vs RMI	0.013 to 0.057	0.020 to 0.195
LR2 vs RMI	0.003 to 0.046	0.029 to 0.208

Model comparison	Suggested management surgery	Suggested management conservative
ADNEX with CA125 vs ADNEX without CA125	0.005 to 0.014	-0.016 to 0.062
ADNEX with CA125 vs SRRisk	0.011 to 0.028	-0.059 to 0.098
ADNEX with CA125 vs LR2	0.024 to 0.041	-0.051 to 0.104
ADNEX with CA125 vs RMI	0.037 to 0.072	0.040 to 0.216
ADNEX without CA125 vs SRRisk	0.001 to 0.018	-0.078 to 0.071
ADNEX without CA125 vs LR2	0.012 to 0.027	-0.073 to 0.080
ADNEX without CA125 vs RMI	0.023 to 0.064	0.008 to 0.202
SRRisk vs LR2	-0.002 to 0.022	-0.088 to 0.101
SRRisk vs RMI	0.012 to 0.059	0.009 to 0.208
LR2 vs RMI	0.003 to 0.045	0.003 to 0.200

Model comparison	Premenopausal patients	Postmenopausal patients
ADNEX with CA125 vs ADNEX without CA125	-0.003 to 0.008	0.004 to 0.013
ADNEX with CA125 vs SRRisk	0.007 to 0.026	0.002 to 0.017
ADNEX with CA125 vs LR2	0.009 to 0.031	0.020 to 0.044
ADNEX with CA125 vs RMI	0.048 to 0.094	0.024 to 0.046
ADNEX without CA125 vs SRRisk	0.003 to 0.022	-0.008 to 0.007
ADNEX without CA125 vs LR2	0.002 to 0.028	0.006 to 0.026
ADNEX without CA125 vs RMI	0.043 to 0.094	0.012 to 0.037
SRRisk vs LR2	-0.011 to 0.016	0.010 to 0.032
SRRisk vs RMI	0.037 to 0.081	0.007 to 0.042
LR2 vs RMI	0.028 to 0.083	-0.017 to 0.015

Model comparison	Oncology centres	Other centres
ADNEX with CA125 vs ADNEX without CA125	0.005 to 0.012	0.000 to 0.012
ADNEX with CA125 vs SRRisk	0.005 to 0.019	-0.010 to 0.033
ADNEX with CA125 vs LR2	0.018 to 0.034	0.017 to 0.046
ADNEX with CA125 vs RMI	0.030 to 0.062	0.035 to 0.108
ADNEX without CA125 vs SRRisk	-0.002 to 0.011	-0.009 to 0.025
ADNEX without CA125 vs LR2	0.010 to 0.024	0.010 to 0.040
ADNEX without CA125 vs RMI	0.021 to 0.053	0.028 to 0.105
SRRisk vs LR2	-0.003 to 0.024	-0.009 to 0.037
SRRisk vs RMI	0.014 to 0.048	0.024 to 0.112
LR2 vs RMI	0.007 to 0.037	-0.004 to 0.085

Supplementary Table 4. Meta-analysis of sensitivity and specificity of Risk of Malignancy Index (RMI), at pre-specified thresholds.

RMI threshold	Sensitivity (95% CI)	Specificity (95% CI)
25	87.2% (82.3–90.9)	71.7% (64.0–78.3)
100	69.5% (62.4–75.7)	90.2% (86.5–93.0)
200	60.4% (53.7–66.8)	95.3% (92.8–96.9)
250	57.0% (50.4–63.3)	95.9% (93.9–97.3)

Supplementary Table 5. Meta-analysis of sensitivity and specificity of International Ovarian Tumour Analysis (IOTA) models at pre-specified risk thresholds.

Risk threshold	Model	Sensitivity (95% CI)	Specificity (95% CI)
1%	LR2	98.6% (97.4–99.2)	13.3% (10.2–17.2)
	SRRisk	97.7% (95.9–98.7)	50.3% (39.6–60.9)
	ADNEX without CA125	98.8% (97.4–99.4)	11.4% (6.9–18.3)
	ADNEX with CA125	99.1% (97.8–99.6)	12.0% (7.2–19.2)
3%	LR2	94.7% (92.9–96.1)	58.4% (53.5–63.1)
	SRRisk	95.8% (91.8–97.8)	63.1% (50.7–73.9)
	ADNEX without CA125	96.3% (93.2–98.0)	55.8% (45.5–65.7)
	ADNEX with CA125	94.7% (90.1–97.2)	59.7% (50.2–68.5)
5%	LR2	90.8% (87.8–93.2)	73.3% (68.4–77.6)
	SRRisk	93.9% (87.8–97.1)	74.1% (66.4–80.6)
	ADNEX without CA125	93.7% (88.5–96.7)	74.6% (68.5–80.0)
	ADNEX with CA125	93.3% (88.2–96.3)	76.4% (70.7–81.3)
10%	LR2	85.6% (80.1–89.7)	87.2% (84.0–89.8)
	SRRisk	91.7% (86.1–95.2)	83.0% (75.5–88.5)
	ADNEX without CA125	91.1% (84.5–95.1)	84.5% (80.1–88.0)
	ADNEX with CA125	91.2% (84.8–95.1)	85.3% (80.9–88.8)
15%	LR2	82.5% (76.5–87.2)	90.2% (87.7–92.3)
	SRRisk	90.8% (85.2–94.4)	85.7% (79.4–90.3)
	ADNEX without CA125	87.7% (80.2–92.7)	88.4% (84.3–91.5)
	ADNEX with CA125	87.3% (79.9–92.3)	89.0% (85.1–92.0)
20%	LR2	80.1% (74.2–84.9)	92.1% (89.9–93.9)
	SRRisk	90.0% (85.3–93.3)	88.2% (83.8–91.5)
	ADNEX without CA125	83.4% (75.0–89.3)	90.3% (86.9–92.9)
	ADNEX with CA125	83.5% (74.4–89.8)	91.5% (88.1–93.9)
25%	LR2	77.5% (71.7–82.4)	93.0% (91.0–94.6)
	SRRisk	89.8% (85.3–93.1)	88.4% (84.0–91.7)
	ADNEX without CA125	79.8% (70.2–86.9)	92.0% (88.9–94.4)
	ADNEX with CA125	80.2% (71.6–86.7)	93.1% (90.1–95.3)
30%	LR2	73.6% (67.7–78.8)	93.8% (92.1–95.2)
	SRRisk	80.9% (67.9–89.4)	93.3% (88.1–96.4)
	ADNEX without CA125	77.9% (68.4–85.2)	93.2% (90.2–95.3)
	ADNEX with CA125	77.3% (67.9–84.5)	94.4% (91.6–96.4)
40%	LR2	68.1% (61.9–73.8)	95.4% (93.8–96.5)
	SRRisk	80.5% (67.6–89.1)	93.4% (88.3–96.4)
	ADNEX without CA125	72.8% (63.0–80.8)	95.0% (92.4–96.8)
	ADNEX with CA125	73.0% (63.4–80.8)	95.8% (93.4–97.3)
50%	LR2	62.7% (57.2–67.9)	96.2% (94.8–97.2)
	SRRisk	57.7% (48.5–66.4)	97.5% (95.9–98.4)
	ADNEX without CA125	66.8% (55.8–76.2)	96.3% (94.1–97.6)
	ADNEX with CA125	66.7% (57.0–75.1)	97.1% (95.5–98.1)

CI, confidence interval; LR2, logistic regression model 2; SRRisk, Simple Rules risk model; ADNEX, Assessment of Different NEoplasias in the adneXa.

Supplementary Figure 6. Centre-specific calibration curves of Risk of Malignancy Index (RMI) for predicting malignancy. ”Other” includes the following small non-oncology centres with low prevalence of malignancy: London and Nottingham from the UK, and Milan 3 and Florence from Italy. The common cut-off of 200 corresponded to an observed proportion ranging from below 20% to about 70%.

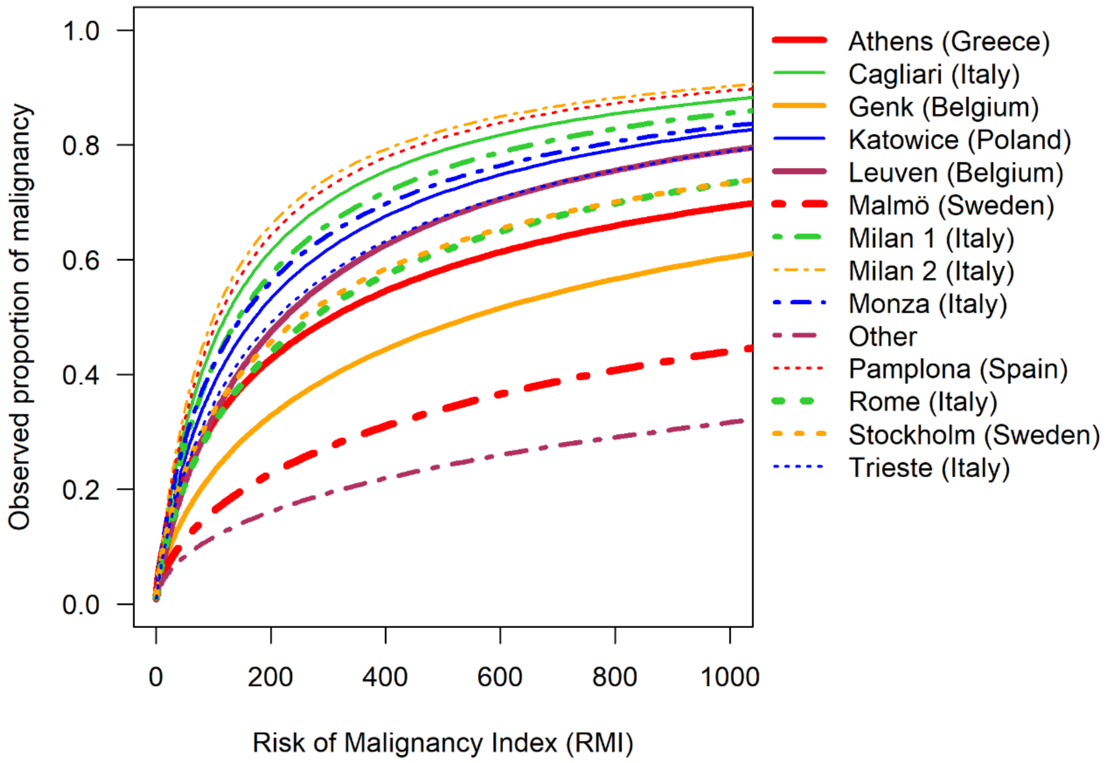

Supplementary Figure 7. Centre-specific calibration curves of logistic regression model 2 (LR2). ”Other” includes the following small non-oncology centres with low prevalence of malignancy: London and Nottingham from the UK, and Milan 3 and Florence from Italy.

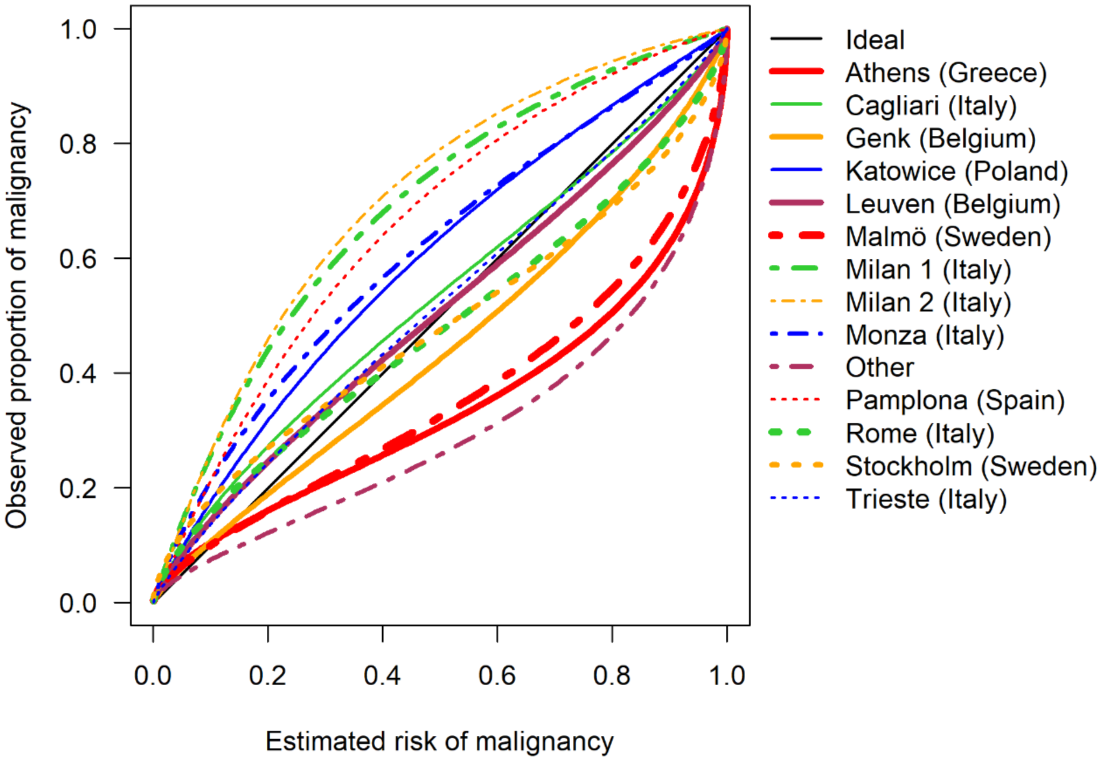

Supplementary Figure 8. Centre-specific calibration curves of Simple Rules risk model (SRRisk). ”Other” includes the following small non-oncology centres with low prevalence of malignancy: London and Nottingham from the UK, and Milan 3 and Florence from Italy.

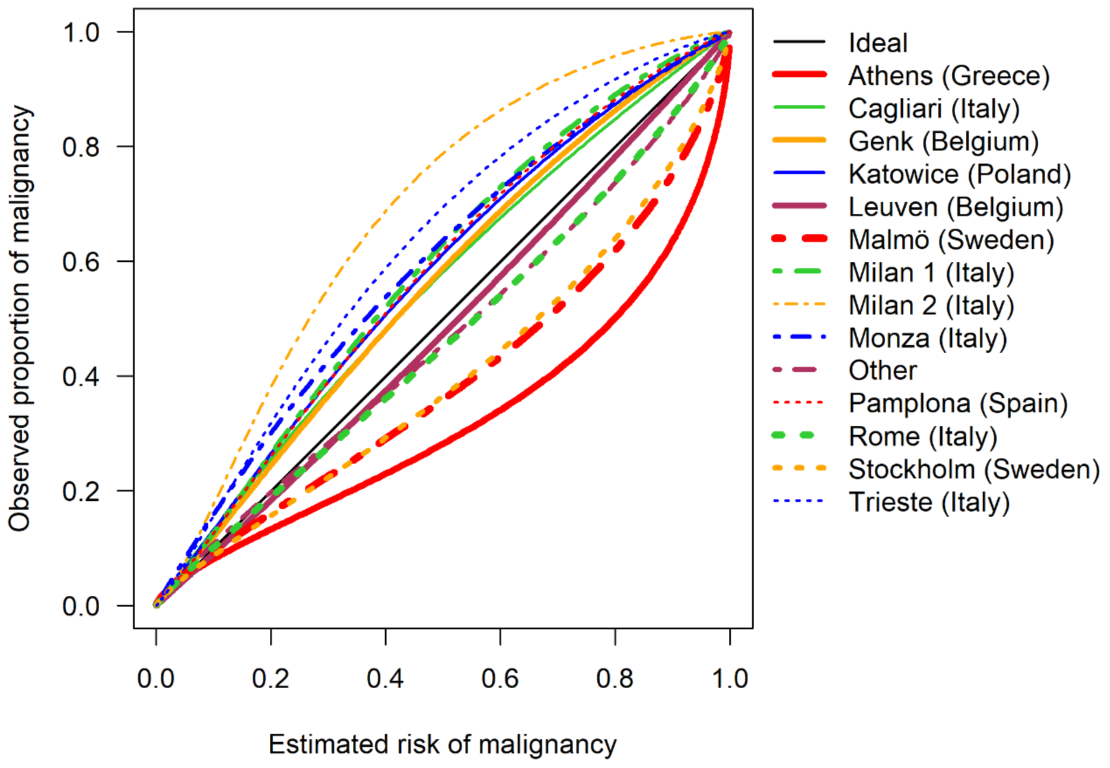

Supplementary Figure 9. Centre-specific calibration curves of Assessment of Different NEoplasias in the adneXa **(**ADNEX) without CA125. ”Other” includes the following small non-oncology centres with low prevalence of malignancy: London and Nottingham from the UK, and Milan 3 and Florence from Italy.

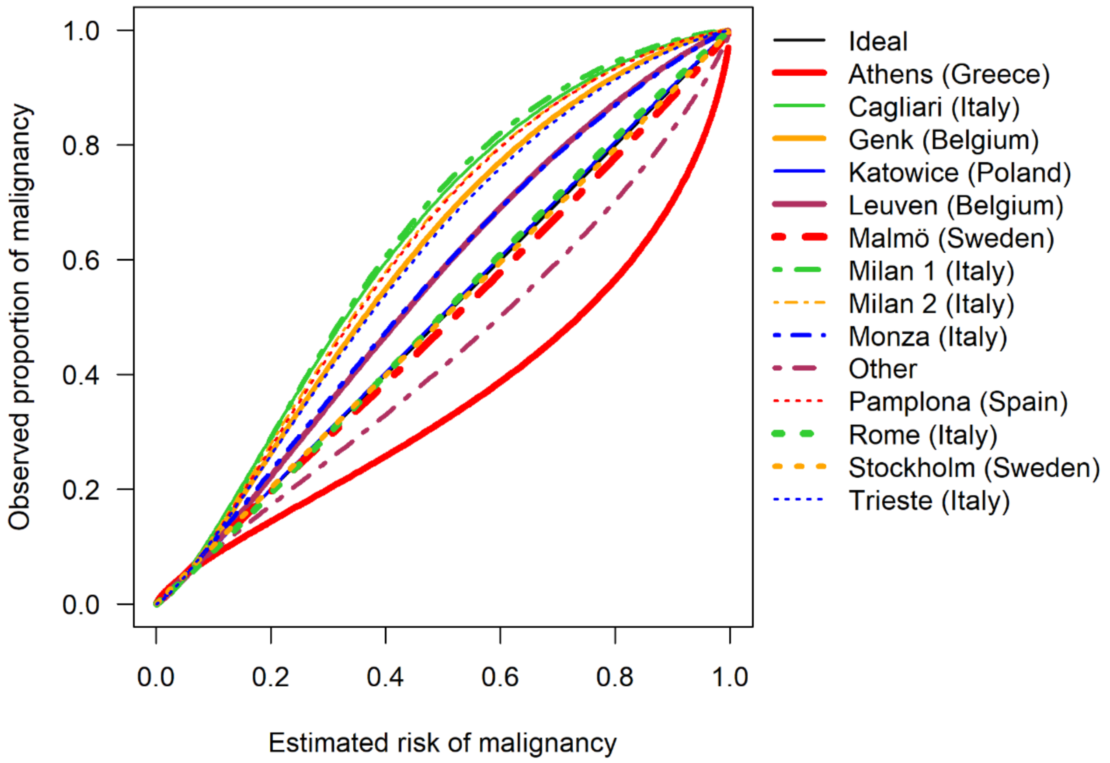

Supplementary Figure 10. Centre-specific calibration curves of Assessment of Different NEoplasias in the adneXa **(**ADNEX) with CA125. ”Other” includes the following smaller non-oncology centres with low prevalence of malignancy: London and Nottingham from the UK, and Milan 3 and Florence from Italy.

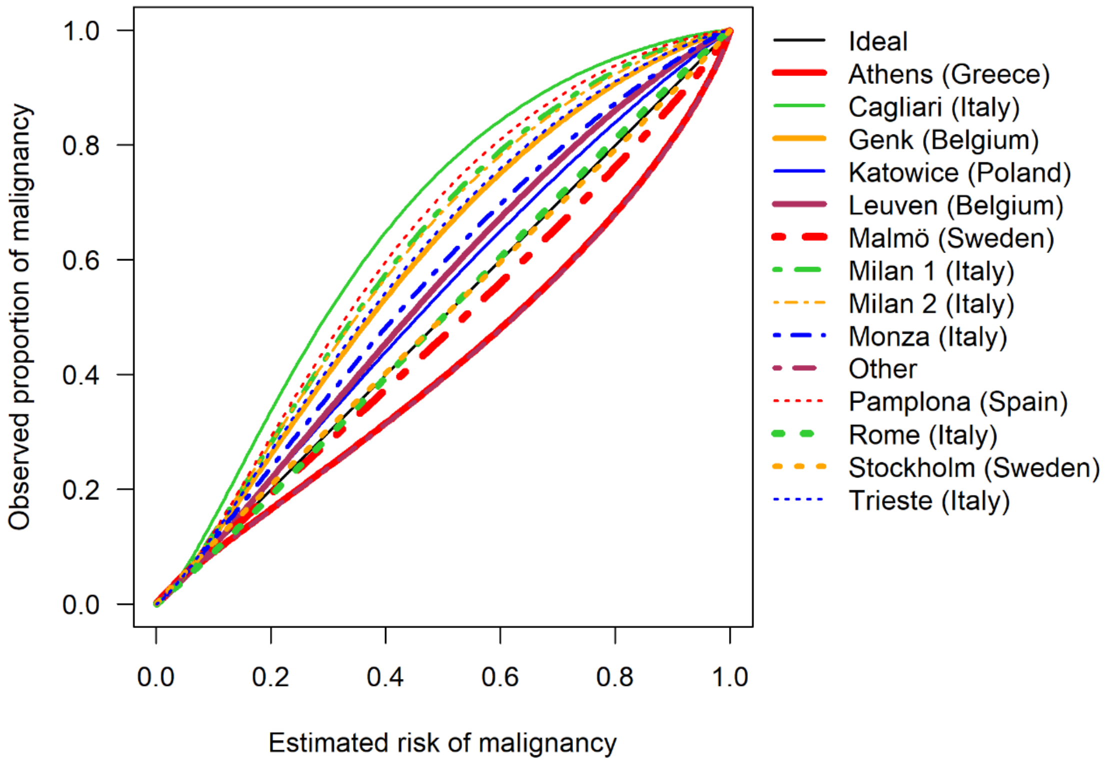

Supplementary Figure 11. Meta-analysis of centre-specific calibration curves of the Risk of Malignancy Index (RMI).

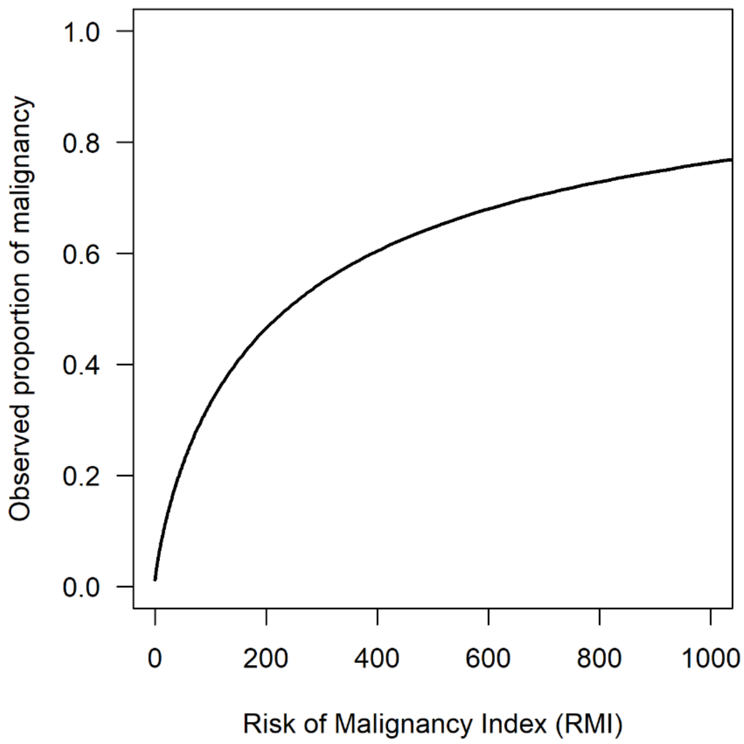

Supplementary Figure 12. Histogram of scores given by the Risk of Malignancy Index (RMI). Results are based on a stacked dataset of the 100 completed datasets following multiple imputation.

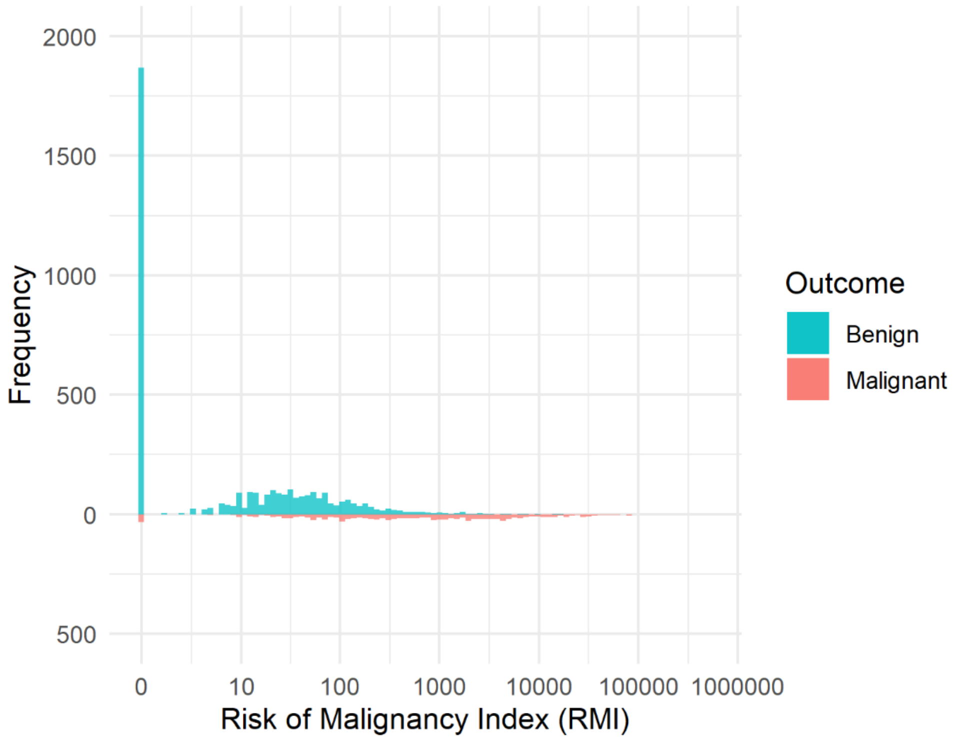

Supplementary Figure 13. Histogram of estimated risks of malignancy given by logistic regression model 2 (LR2). Results are based on a stacked dataset of the 100 completed datasets following multiple imputation.

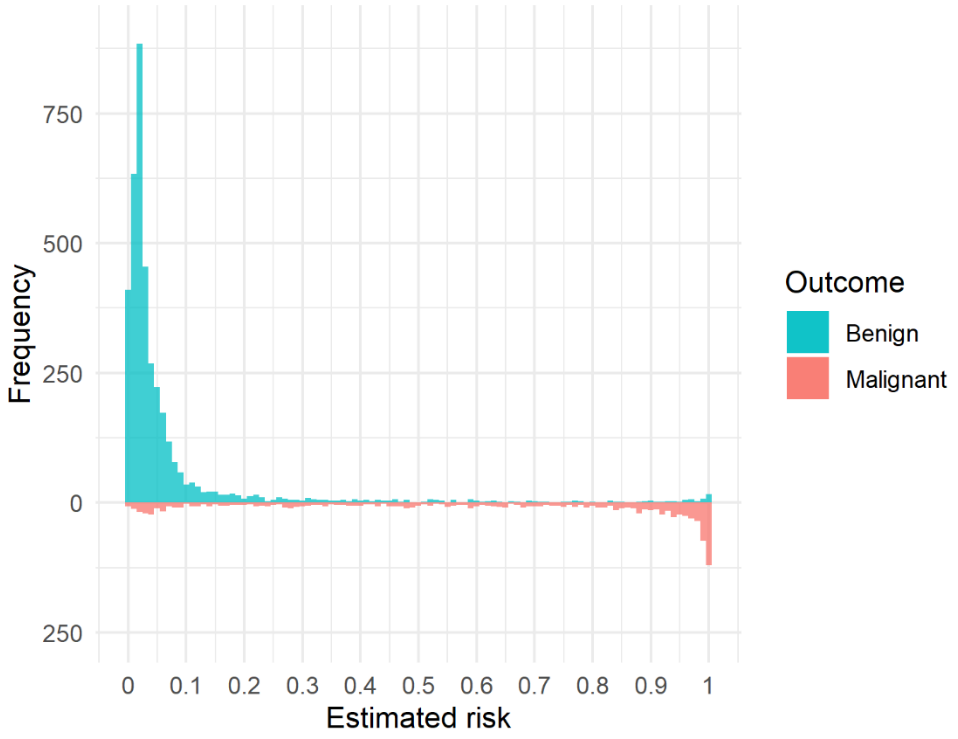

Supplementary Figure 14. Histogram of estimated risks of malignancy given by Simple Rules risk model (SRRisk). Results are based on a stacked dataset of the 100 completed datasets following multiple imputation.

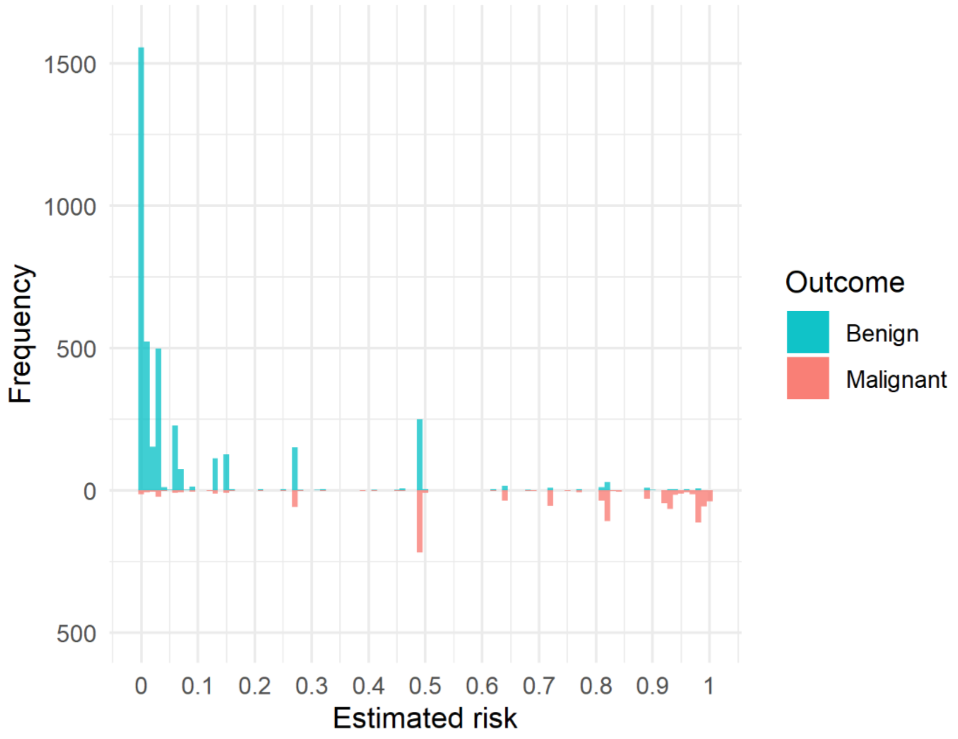

Supplementary Figure 15. Histogram of estimated risks of malignancy given by Assessment of Different NEoplasias in the adneXa (ADNEX) without CA125. Results are based on a stacked dataset of the 100 completed datasets following multiple imputation.

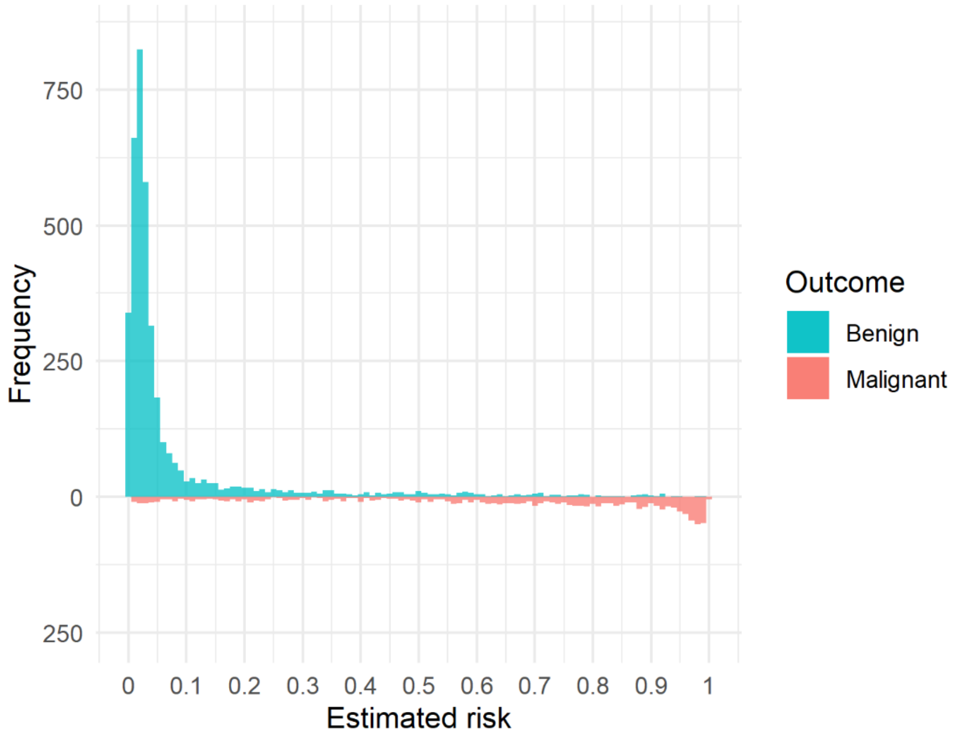

Supplementary Figure 16. Histogram of estimated risks of malignancy given by Assessment of Different NEoplasias in the adneXa (ADNEX) with CA125. Results are based on a stacked dataset of the 100 completed datasets following multiple imputation.

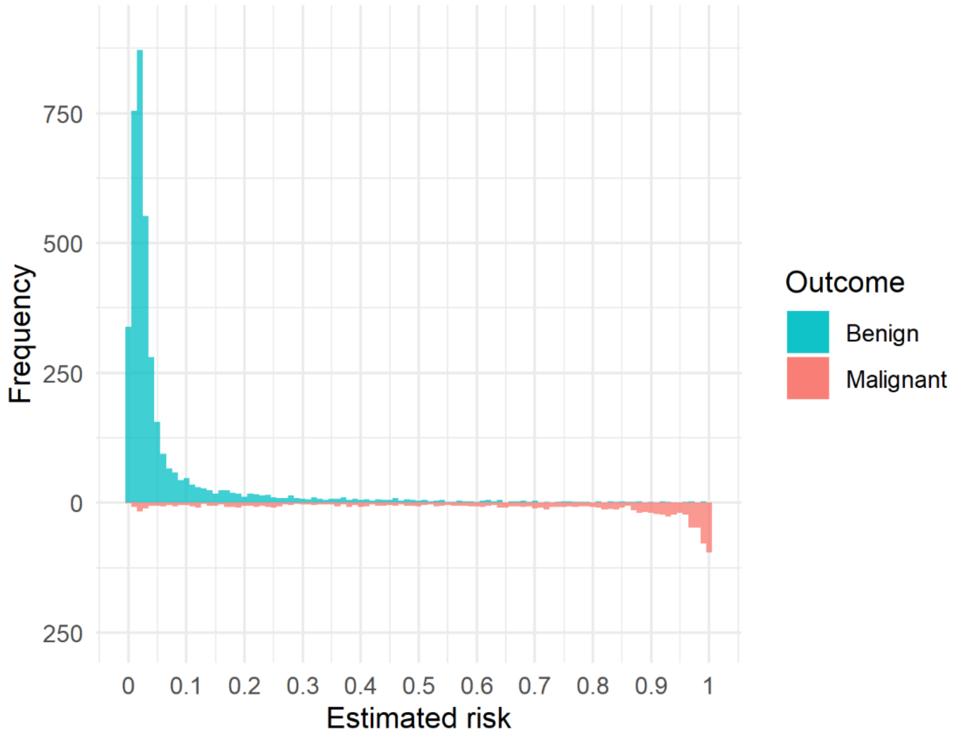

Supplementary Table 6. Area under the receiver operating characteristic curves (AUC) of Assessment of Different NEoplasias in the adnexa (ADNEX) for each pair of outcome categories. The results are based on a pooled analysis, not on meta-analysis, due to low numbers for some outcome categories.

**Pair of outcome categories**	**AUC (95% CI)**	
	**ADNEX without CA125**	**ADNEX with CA125**
Benign vs borderline	0.89 (0.87–0.92)	0.90 (0.87–0.92)
Benign vs stage I primary ovarian cancer	0.95 (0.93–0.97)	0.95 (0.94–0.97)
Benign vs stage II–IV primary ovarian cancer	0.97 (0.96–0.98)	0.98 (0.98–0.99)
Benign vs secondary metastasis	0.94 (0.92–0.96)	0.95 (0.93–0.97)
Borderline vs stage I primary ovarian cancer	0.78 (0.73–0.82)	0.77 (0.72–0.81)
Borderline vs stage II–IV primary ovarian cancer	0.90 (0.88–0.93)	0.92 (0.90–0.94)
Borderline vs secondary metastasis	0.88 (0.84–0.91)	0.88 (0.84–0.91)
Stage I vs stage II–IV primary ovarian cancer	0.72 (0.68–0.76)	0.81 (0.77–0.85)
Stage I primary ovarian cancer vs secondary metastasis	0.75 (0.69–0.80)	0.75 (0.70–0.80)
Stage II–IV primary ovarian cancer vs secondary metastasis	0.66 (0.61–0.71)	0.78 (0.73–0.82)

CI, confidence interval.

Supplementary Figure 17. Multinomial calibration curves of Assessment of Different NEoplasias in the adneXa (ADNEX) without CA125. The results are based on a pooled analysis, not on meta-analysis, due to low numbers for some outcome categories.

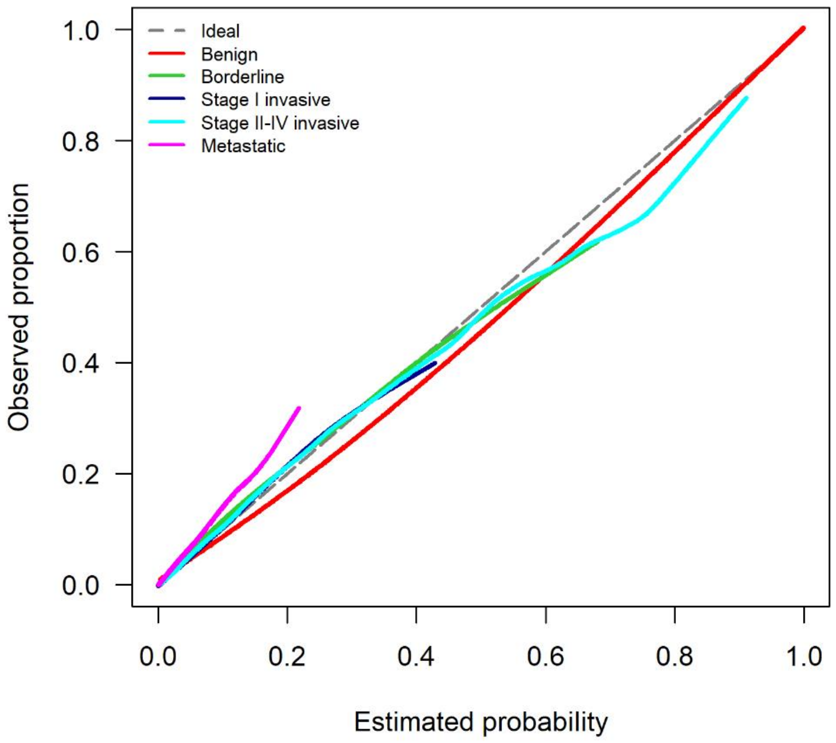

Supplementary Figure 18. Multinomial calibration curves of Assessment of Different NEoplasias in the adneXa (ADNEX) with CA125. The results are based on a pooled analysis, not on meta-analysis, due to low numbers for some outcome categories.

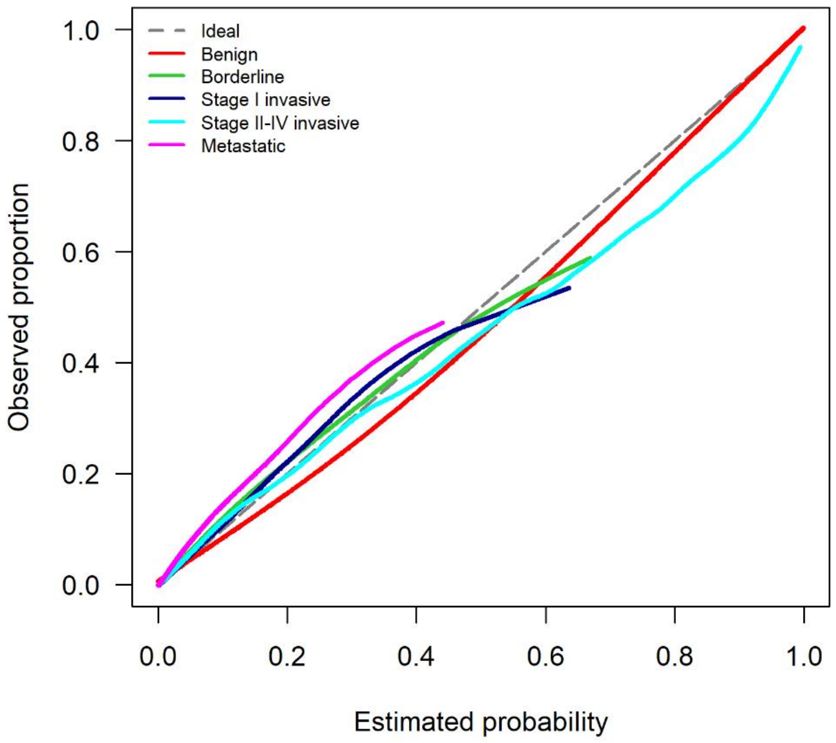

Supplementary Table 7. Overview of outcomes and actual management for all pre-specified subgroups.

		Outcome[a]			Actual management[b]			Missing
**Subgroup**	**N**	**Ben**	**Mal**	**Unc**	**Surg**	**Cons**	**Unk**	**CA125, n (%)**
Based on actual management								
Surgery within 120 days, no follow-up scan	2489	1544	945	0	2489	0	0	766 (31%)
At least one follow-up scan received	1958	1762	22	174	0	1958	0	1562 (80%)
Based on suggested management at inclusion								
Suggested management surgery	2579	1487	956	136	2326	133	120	835 (32%)
Suggested management conservative	2326	1954	22	350	312	1825	189	1785 (77%)
Based on menopausal status at inclusion								
Premenopausal	2754	2121	367	266	1393	1171	190	1626 (59%)
Postmenopausal	2151	1320	611	220	1245	787	119	994 (46%)
Based on type of centre								
Examined in oncology centre	3094	1973	804	317	1829	1044	221	1433 (46%)
Examined in other (non-oncology) centre	1811	1468	174	169	809	914	88	1187 (66%)

The numbers for subgroups based on actual management do not sum to 4905: some patients were operated without follow-up scan after 120 days, and for some patients we had no information since the inclusion scan.

[a] Criteria for uncertain outcome are shown in Table 1 in the main text. In case of uncertain outcome, we used multiple imputation to classify the mass as benign or malignant at inclusion. In one sensitivity analysis we used a broader definition of uncertain outcome.

[b] Surgery, surgery without any follow-up scan before surgery; Conservative, at least one follow-up scan; Conservative management means that surgery could be performed at any time during follow-up. Unknown management means that we have no information after the inclusion scan.

Supplementary Figure 19. Meta-analysis of calibration of Risk of Malignancy Index (RMI) in patients who underwent surgery within 120 days after inclusion without a follow-up scan (n=2489).

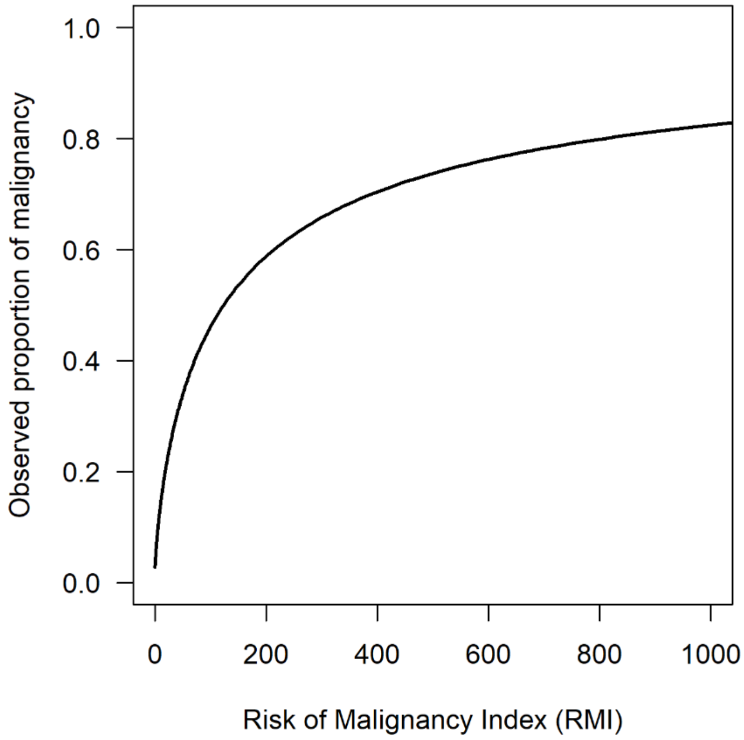

Supplementary Figure 20. Meta-analysis of calibration of International Ovarian Tumour Analysis (IOTA) models in patients who underwent surgery within 120 days after inclusion without a follow-up scan (n=2489). LR2, logistic regression model 2; SRRisk, Simple Rules risk model; ADNEX, Assessment of Different NEoplasias in the adneXa; Intercept, calibration intercept; Slope, calibration slope.

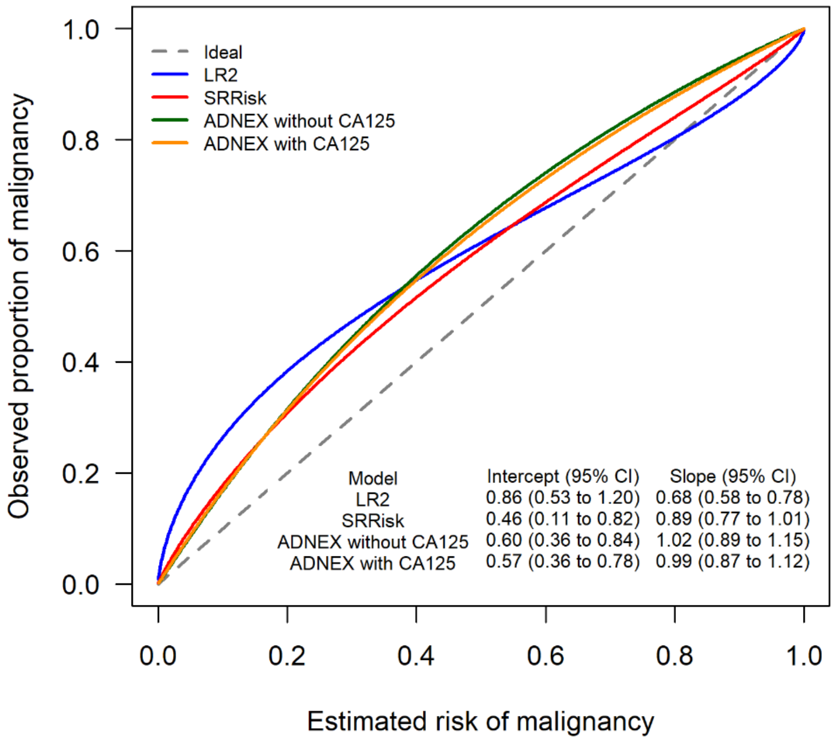

Supplementary Figure 21. Meta-analysis of calibration of the Risk of Malignancy Index (RMI) in patients with at least one follow-up scan (n=1958).

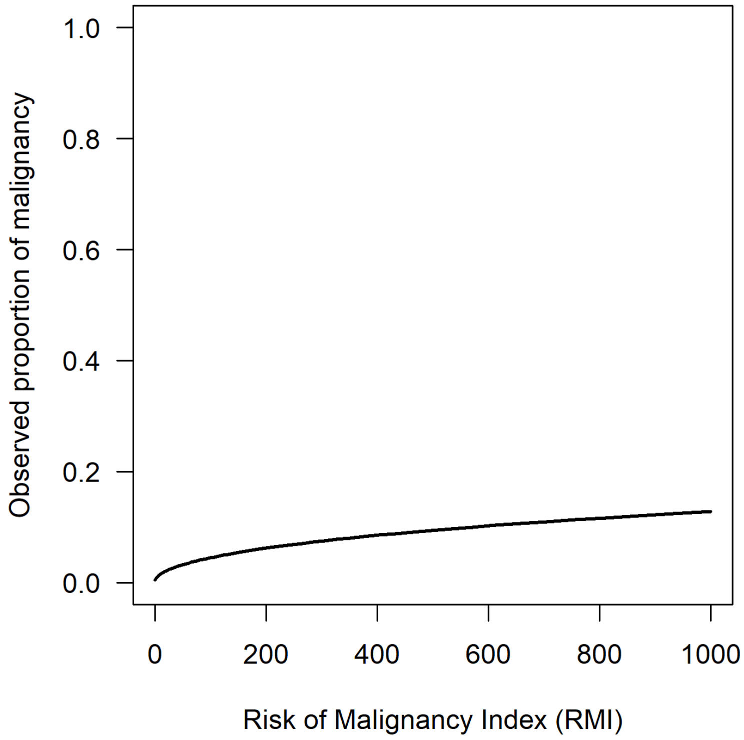

Supplementary Figure 22. Meta-analysis of calibration of International Ovarian Tumour Analysis (IOTA) models in patients with at least one follow-up scan (n=1958). LR2, logistic regression model 2; SRRisk, Simple Rules risk model; ADNEX, Assessment of Different NEoplasias in the adneXa; w/o, without; w/, with; Intercept, calibration intercept; Slope, calibration slope.

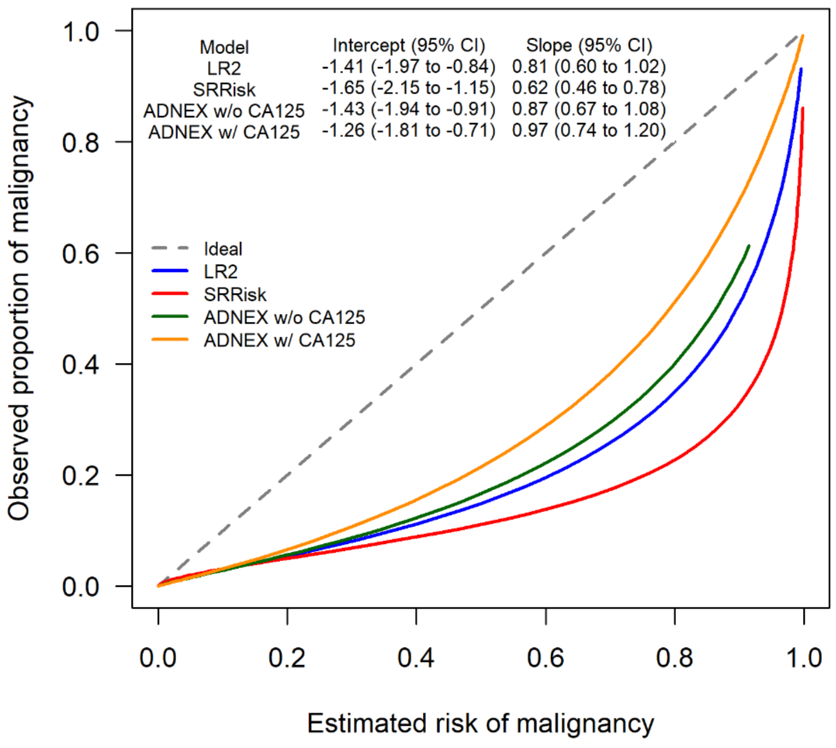

Supplementary Figure 23. Meta-analysis of calibration of the Risk of Malignancy Index (RMI) in patients for whom the ultrasound examiner suggested surgery (n=2579).

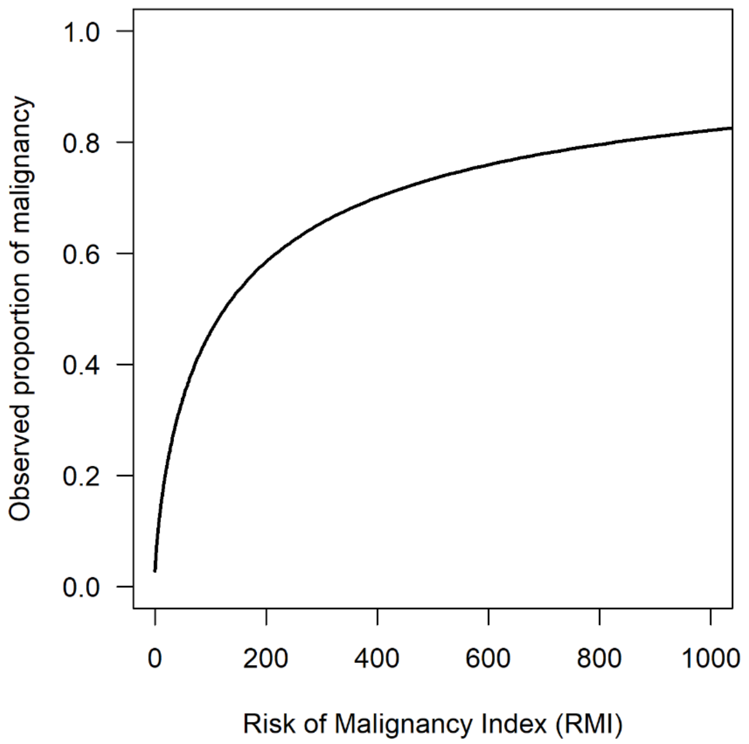

Supplementary Figure 24. Meta-analysis of calibration of International Ovarian Tumour Analysis (IOTA) models in patients for whom the ultrasound examiner suggested surgery (n=2579). LR2, logistic regression model 2; SRRisk, Simple Rules risk model; ADNEX, Assessment of Different NEoplasias in the adneXa; Intercept, calibration intercept; Slope, calibration slope.

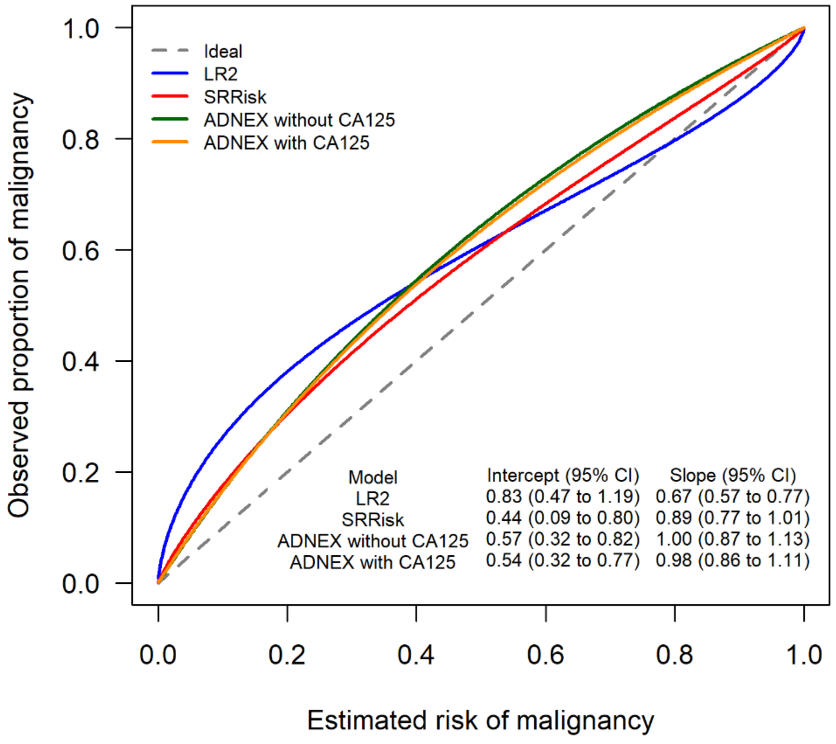

Supplementary Figure 25. Meta-analysis of calibration of Risk of Malignancy Index (RMI) in patients for whom the ultrasound examiner suggested conservative management with follow-up (n=2326).

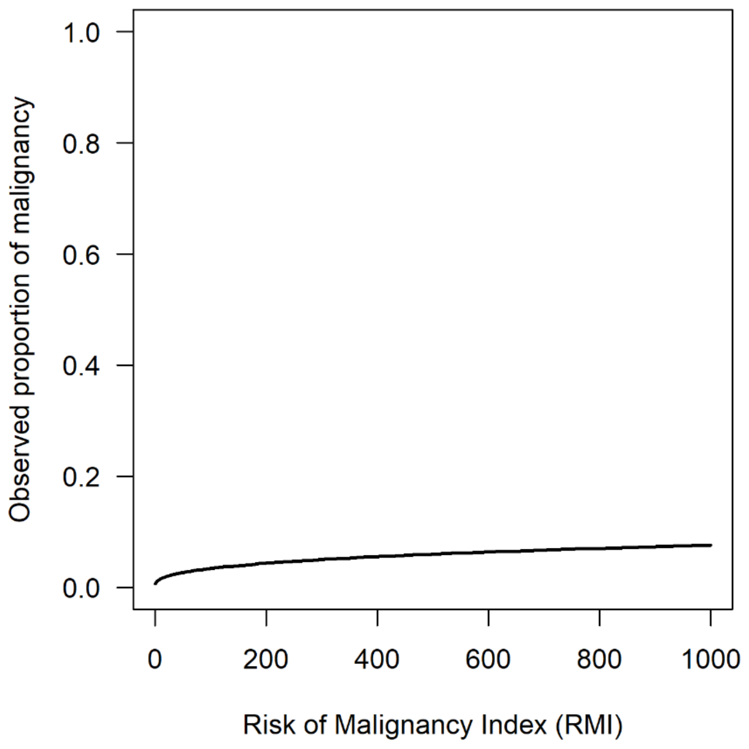

Supplementary Figure 26. Meta-analysis of calibration of International Ovarian Tumour Analysis (IOTA) models in patients for whom the ultrasound examiner suggested conservative management with follow-up (n=2326). LR2, logistic regression model 2; SRRisk, Simple Rules risk model; ADNEX, Assessment of Different NEoplasias in the adneXa; w/o, without; w/, with; Intercept, calibration intercept; Slope, calibration slope.

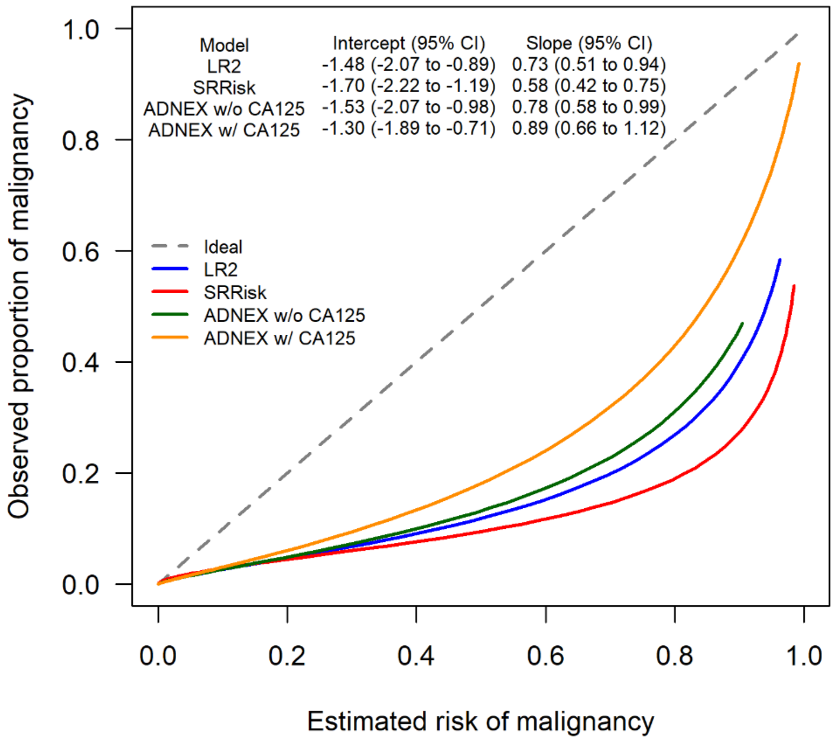

Supplementary Figure 27. Meta-analysis of calibration of the Risk of Malignancy Index (RMI) in premenopausal patients (n=2754).

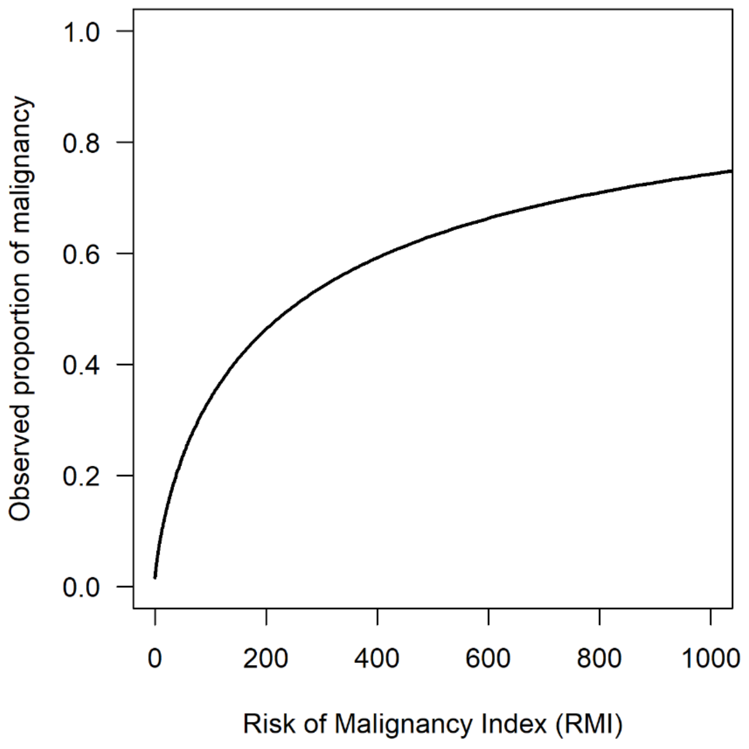

Supplementary Figure 28. Meta-analysis of calibration of International Ovarian Tumour Analysis (IOTA) models in premenopausal patients (n=2754). LR2, logistic regression model 2; SRRisk, Simple Rules risk model; ADNEX, Assessment of Different NEoplasias in the adneXa; Intercept, calibration intercept; Slope, calibration slope.

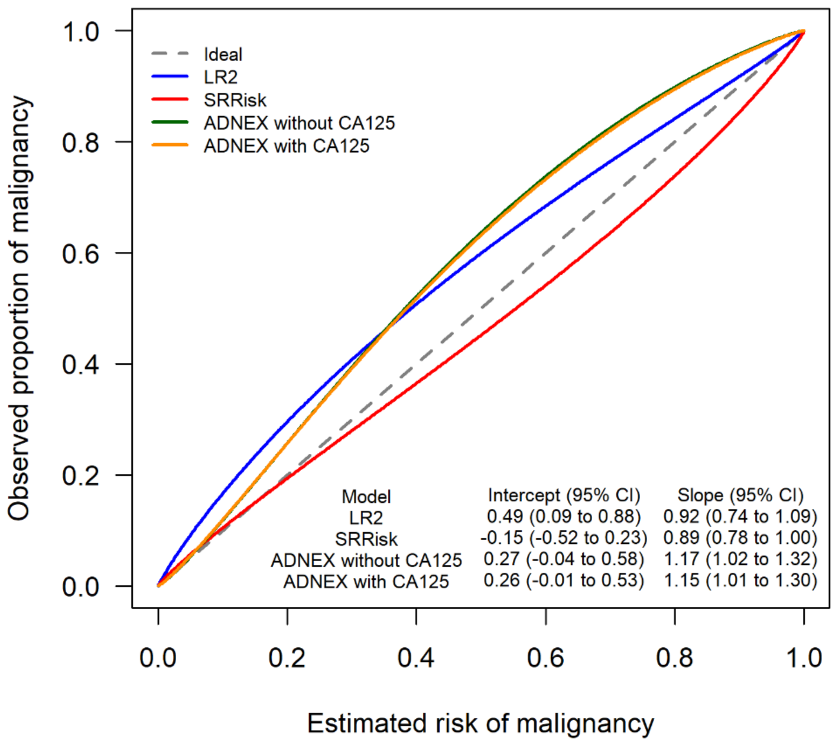

Supplementary Figure 29. Meta-analysis of calibration of the risk of malignancy index (RMI) in postmenopausal patients (n=2151).

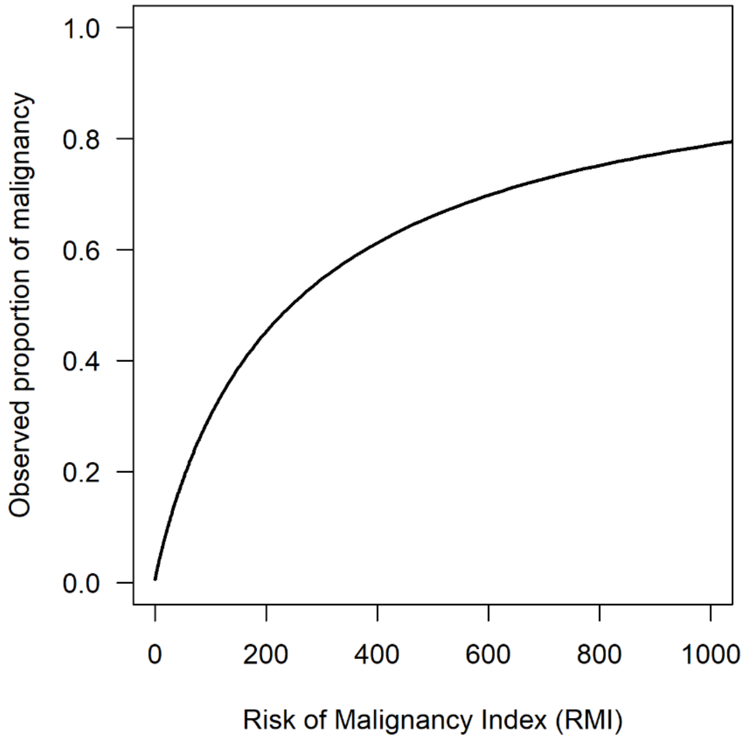

Supplementary Figure 30. Meta-analysis of calibration of International Ovarian Tumour Analysis (IOTA) models in postmenopausal patients (n=2151). LR2, logistic regression model 2; SRRisk, Simple Rules risk model; ADNEX, Assessment of Different NEoplasias in the adneXa; Intercept, calibration intercept; Slope, calibration slope.

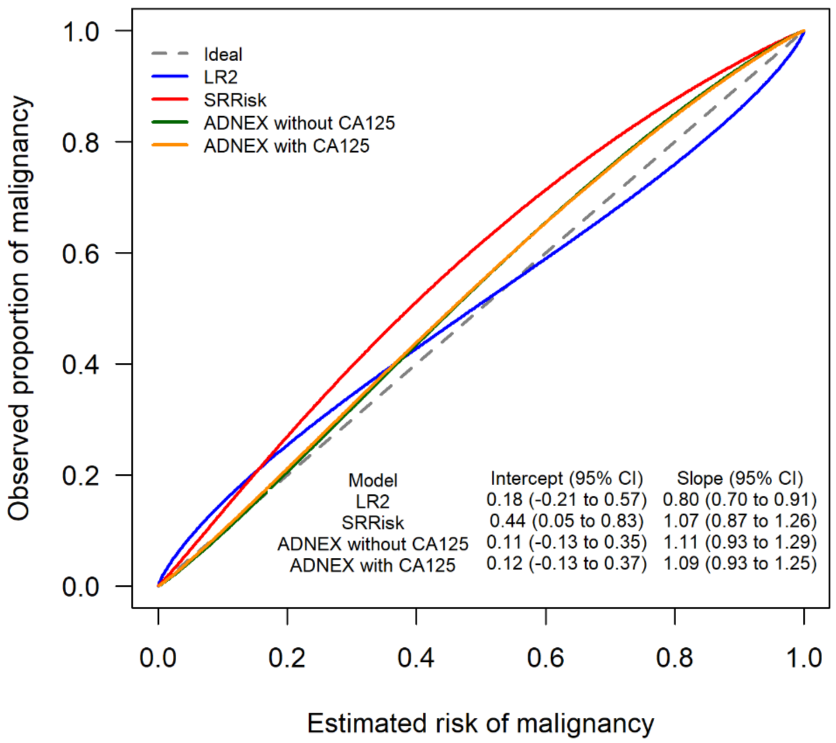

Supplementary Figure 31. Meta-analysis of calibration curves of the Risk of Malignancy Index (RMI) in patients examined in oncology centres (n=3094).

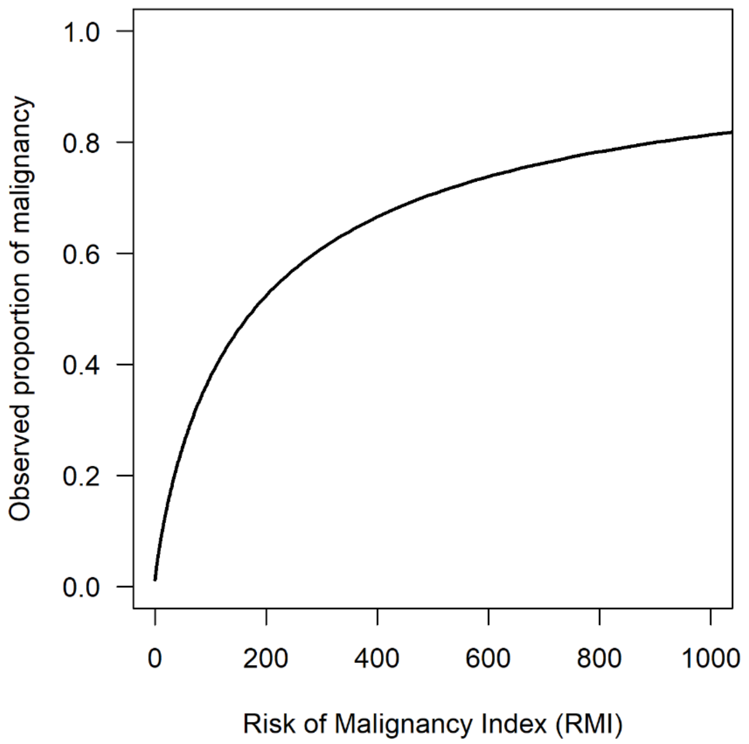

Supplementary Figure 32. Meta-analysis of calibration of International Ovarian Tumour Analysis (IOTA) models in patients examined in oncology centres (n=3094). LR2, logistic regression model 2; SRRisk, Simple Rules risk model; ADNEX, Assessment of Different NEoplasias in the adneXa; Intercept, calibration intercept; Slope, calibration slope.

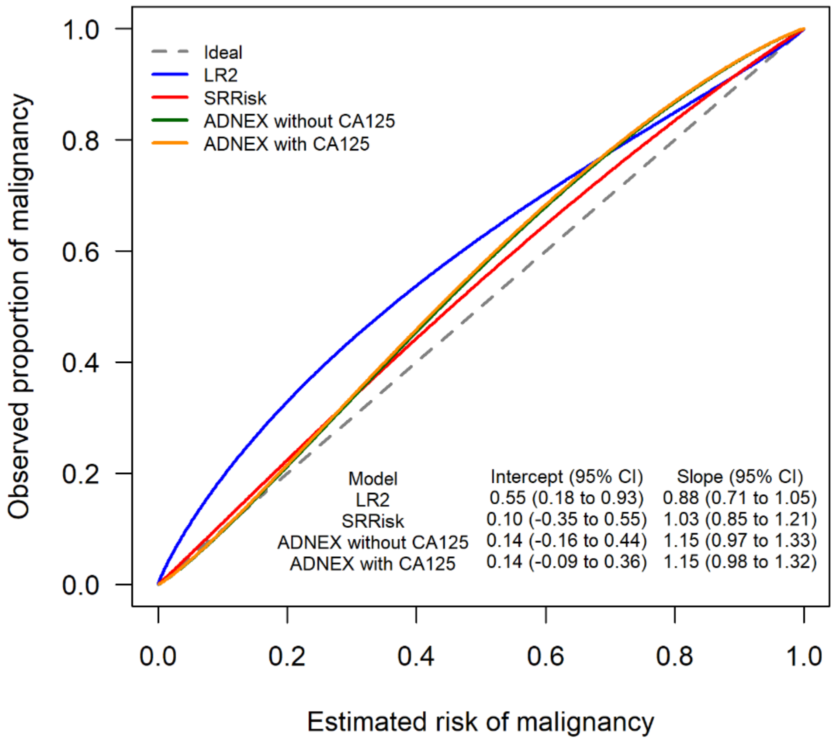

Supplementary Figure 33. Meta-analysis of calibration of the Risk of Malignancy Index (RMI) in patients examined in non-oncology centres (n=1811).

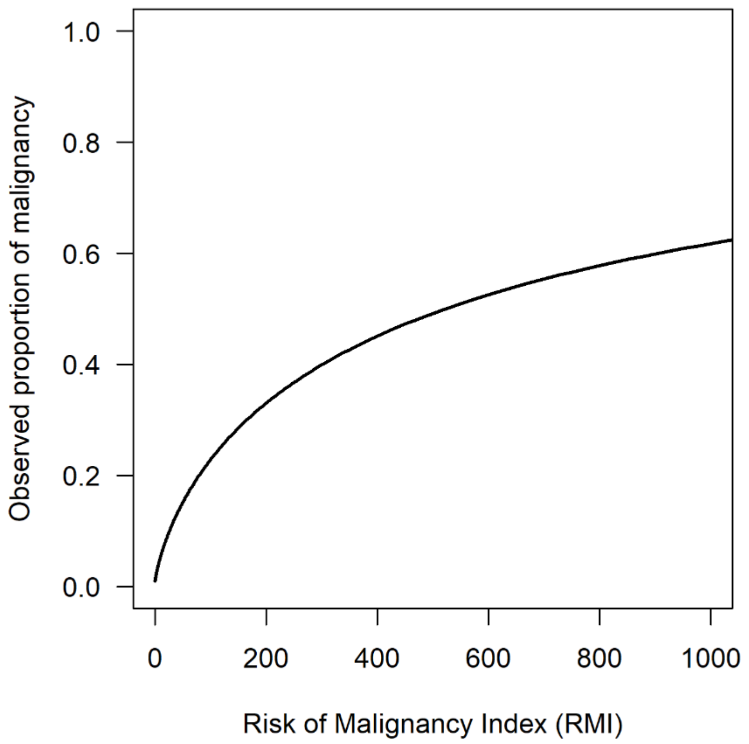

Supplementary Figure 34. Meta-analysis of calibration of International Ovarian Tumour Analysis (IOTA) models in patients examined in non-oncology centres (n=1811). LR2, logistic regression model 2; SRRisk, Simple Rules risk model; ADNEX, Assessment of Different NEoplasias in the adneXa; Intercept, calibration intercept; Slope, calibration slope.

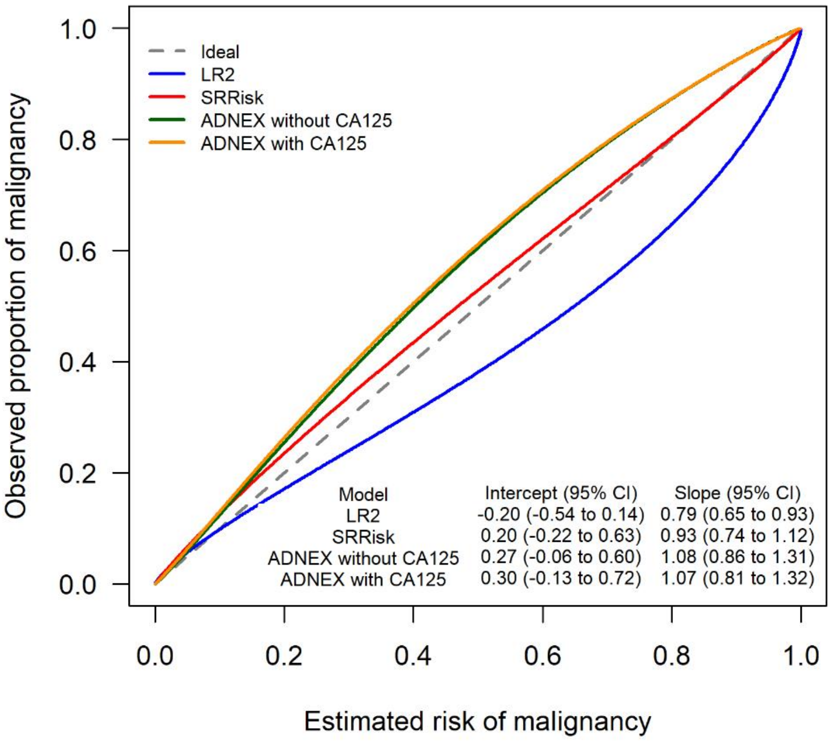

Supplementary Figure 35. Summary forest plot of the receiver operating characteristic curve (AUC) per model when patients with uncertain outcome are omitted (sensitivity analysis; n=4419). RMI, risk of malignancy index; LR2, logistic regression model 2; SRRisk, Simple Rules risk model; ADNEX, Assessment of Different NEoplasias in the adneXa; CI, confidence interval; PI, prediction interval.

Model	AUC (95% CI)	95% PI
RMI	0.89 (0.86 to 0.92)	(0.73 to 0.96)
LR2	0.92 (0.90 to 0.94)	(0.84 to 0.96)
SRRisk	0.94 (0.91 to 0.95)	(0.83 to 0.98)
ADNEX without CA125	0.94 (0.91 to 0.96)	(0.83 to 0.98)
ADNEX with CA125	0.95 (0.92 to 0.96)	(0.84 to 0.98)

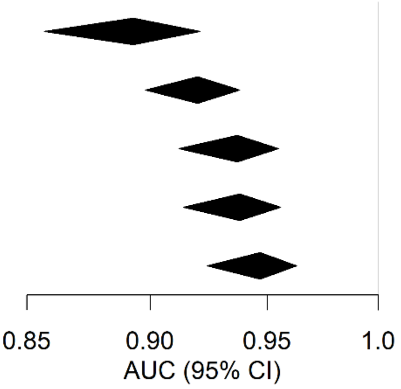

Supplementary Figure 36. Meta-analysis of calibration of the Risk of Malignancy Index (RMI) when patients with uncertain outcome are omitted (sensitivity analysis; n=4419).

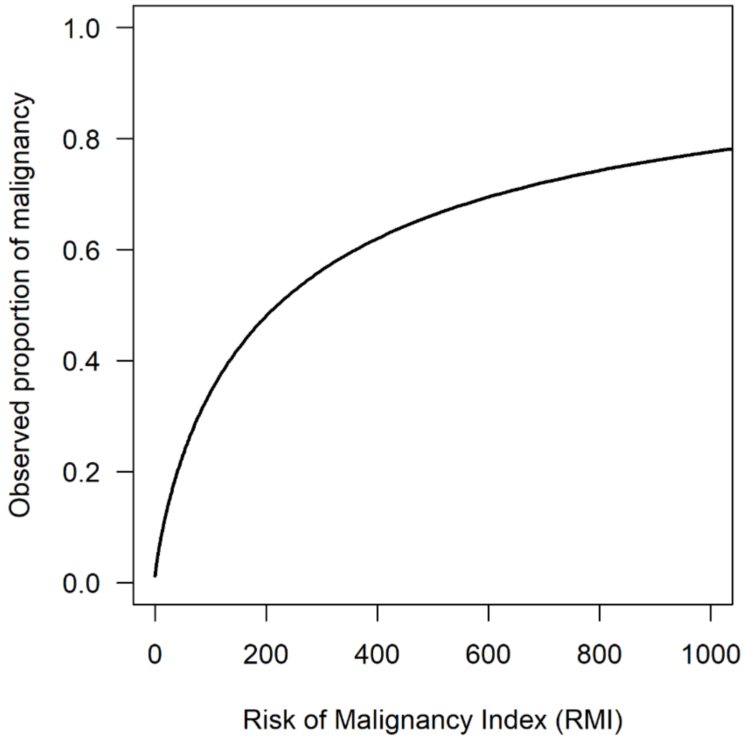

Supplementary Figure 37. Meta-analysis of calibration of International Ovarian Tumour Analysis (IOTA) models when patients with uncertain outcome are omitted (sensitivity analysis; n=4419). LR2, logistic regression model 2; SRRisk, Simple Rules risk model; ADNEX, Assessment of Different NEoplasias in the adneXa; Intercept, calibration intercept; Slope, calibration slope.

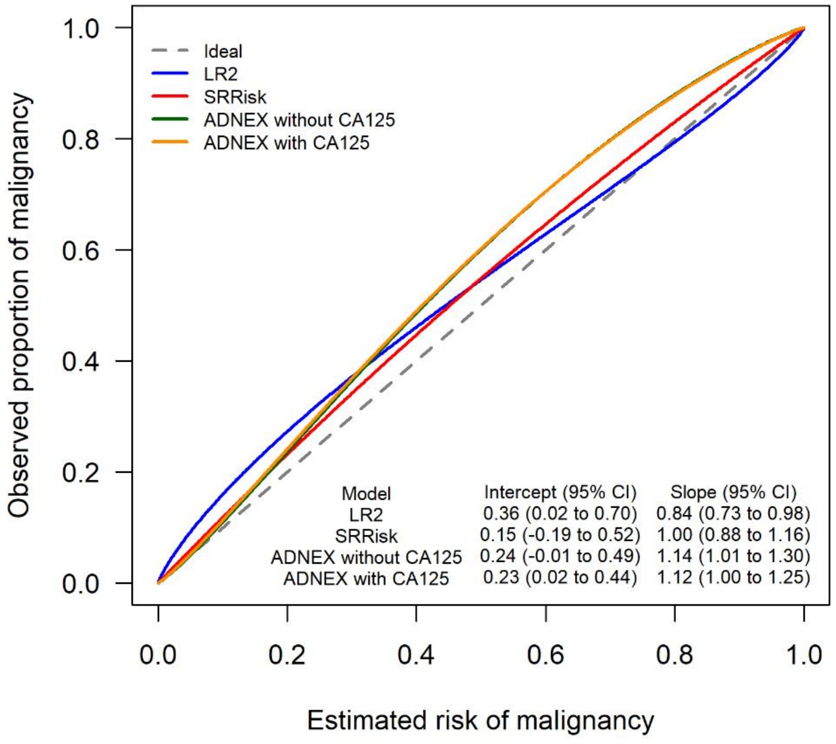

Supplementary Figure 38. Summary forest plot of areas under the receiver operating characteristic curve (AUC) per model when a broader definition of uncertain outcome is used (sensitivity analysis; n=4905). Multiple imputation of the uncertain outcomes was used. RMI, risk of malignancy index; LR2, logistic regression model 2; SRRisk, Simple Rules risk model; ADNEX, Assessment of Different NEoplasias in the adneXa; CI, confidence interval; PI, prediction interval.

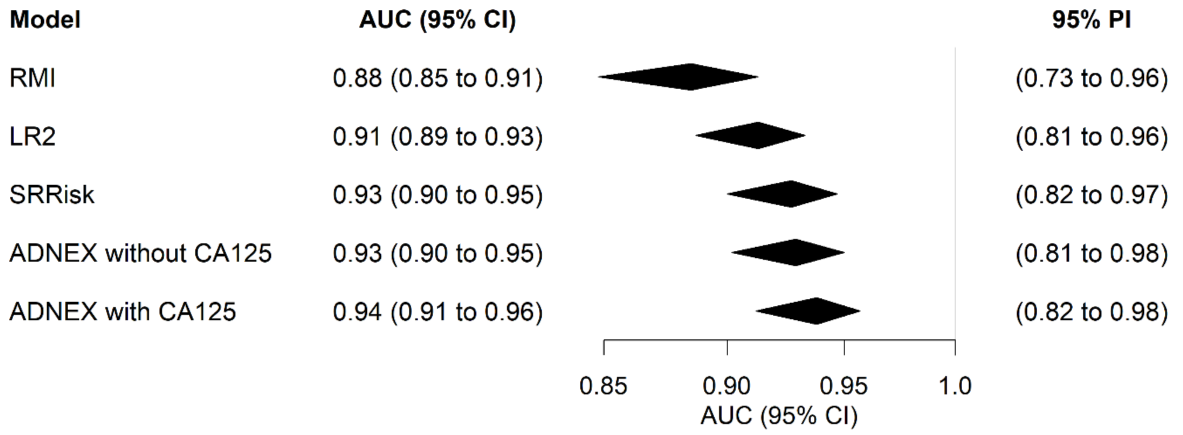

Supplementary Figure 39. Meta-analysis of calibration of the Risk of Malignancy Index (RMI) when a broader definition of uncertain outcome is used (sensitivity analysis; n=4905). Multiple imputation of the uncertain outcomes was used.

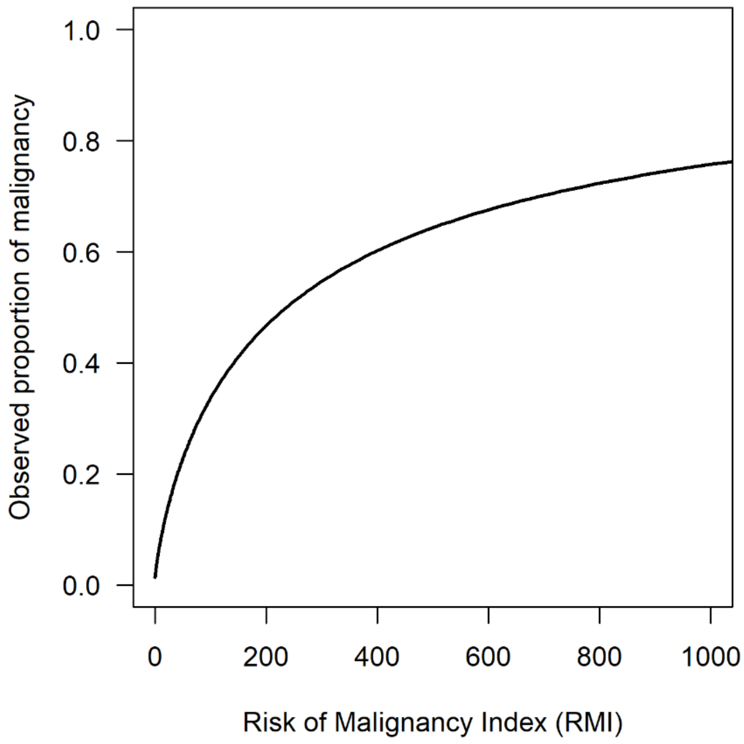

Supplementary Figure 40. Meta-analysis of calibration of International Ovarian Tumour Analysis (IOTA) models when a broader definition of uncertain outcome is used (sensitivity analysis; n=4905). Multiple imputation of the uncertain outcomes was used. LR2, logistic regression model 2; SRRisk, Simple Rules risk model; ADNEX, Assessment of Different NEoplasias in the adneXa; Intercept, calibration intercept; Slope, calibration slope.

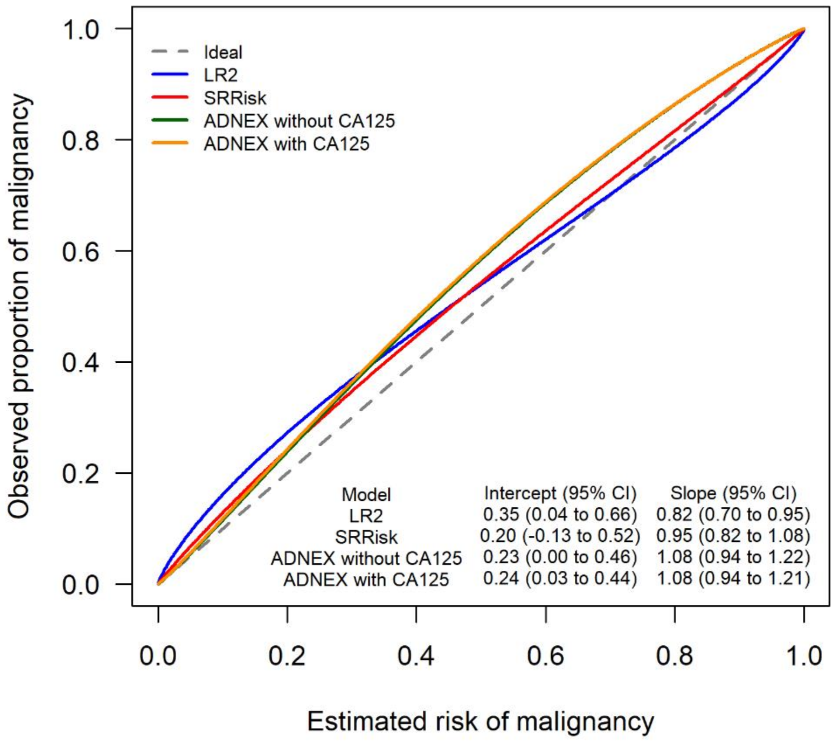

Supplementary Figure 41. Summary forest plot of area under the receiver operating characteristic curve (AUC) per model for patients who underwent surgery within 120 days after inclusion without a follow-up scan in all 36 centres (sensitivity analysis; n=3369). RMI, risk of malignancy index; LR2, logistic regression model 2; SRRisk, Simple Rules risk model; ADNEX, Assessment of Different NEoplasias in the adneXa; CI, confidence interval; PI, prediction interval.

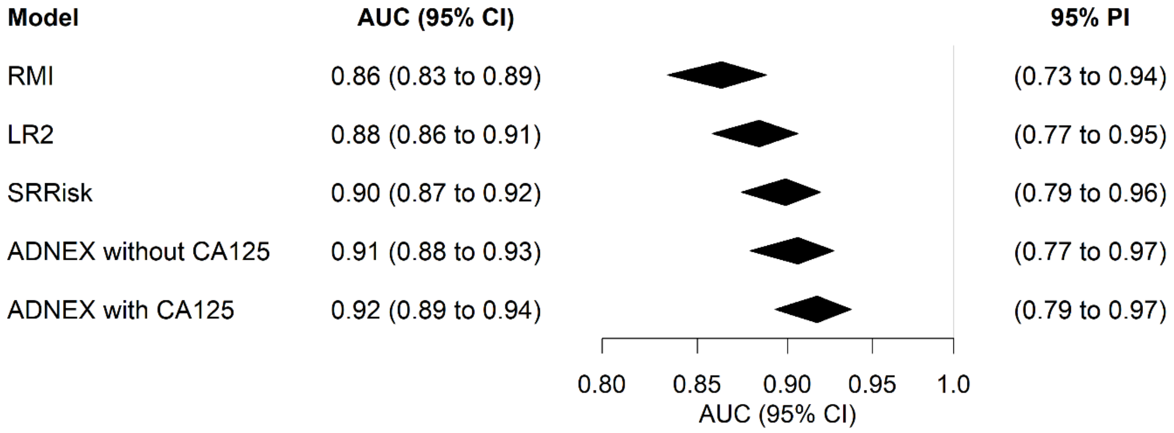

Supplementary Figure 42. Meta-analysis of calibration for the risk of malignancy Index (RMI) for patients who underwent surgery within 120 days after inclusion without a follow-up scan in all 36 centres (sensitivity analysis; n=3369).

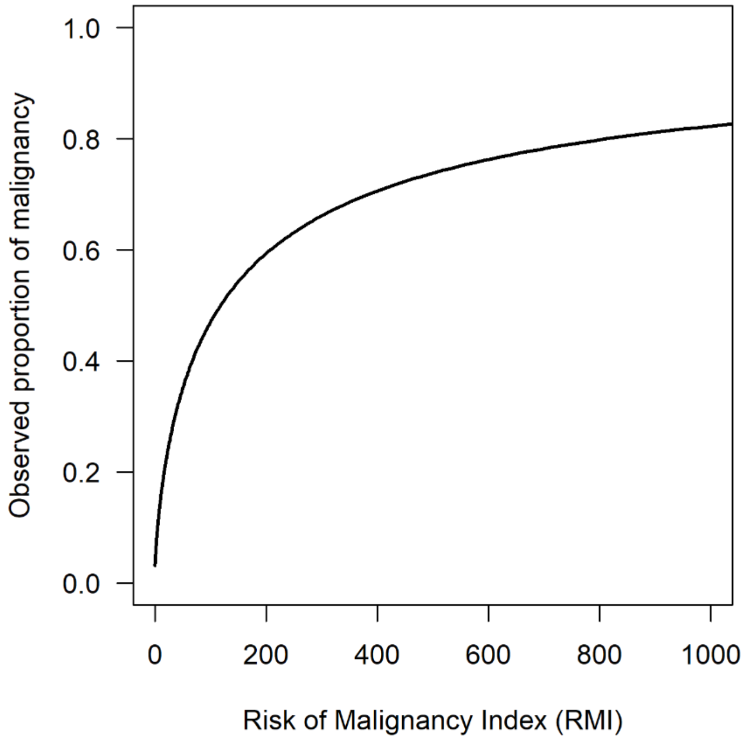

Supplementary Figure 43. Meta-analysis of calibration International Ovarian Tumour Analysis (IOTA) models for patients who underwent surgery within 120 days after inclusion without a follow-up scan in all 36 centres (sensitivity analysis; n=3369). LR2, logistic regression model 2; SRRisk, Simple Rules risk model; ADNEX, Assessment of Different NEoplasias in the adneXa; Intercept, calibration intercept; Slope, calibration slope.

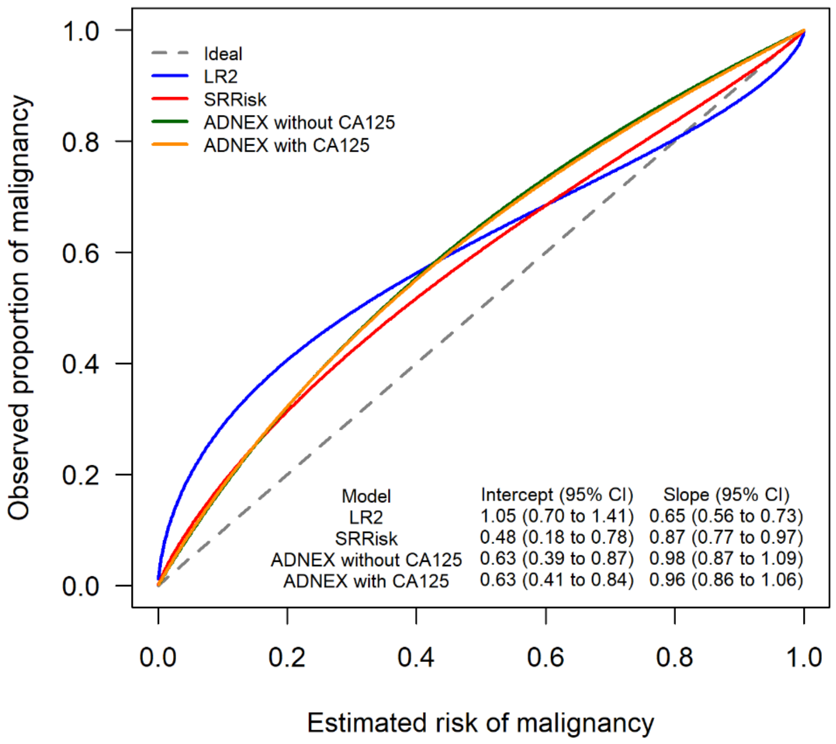

Appendix 1 – IOTA5 protocol

International Ovarian Tumour Analysis (IOTA) Phase 5

A multicentre study to examine the short and long term outcomes of the conservative management of benign-looking adnexal masses and the pre-operative characterisation of ovarian tumours

Study Co-ordinators

Tom Bourne, Lil Valentin, Dirk Timmerman

Contact details:

Dirk Timmerman, MD, PhD

Department of Obstetrics and Gynaecology, University Hospitals Leuven,

Herestraat 49, B-3000 Leuven, BELGIUM.

Telephone: + 32 16 344201 (office) Fax: + 32 16 344205

+ 32 16 344215 (secretary)

E-mail: dirk.timmerman@uzleuven.be

Steering Committee

Dirk Timmerman (University Hospitals, KU Leuven)

Tom Bourne (Imperial College, London)

Antonia C. Testa (Università Cattolica di Sacro Cuore, Roma)

Lil Valentin (University of Lund / Malmö)

Ben Van Calster (KU Leuven)

Sabine Van Huffel (ESAT-SISTA, KU Leuven)

Ignace Vergote (University Hospitals, KU Leuven)

SUMMARY

The medium to long term behaviour of benign-looking adnexal masses that do not undergo surgery is unknown. It is possible for these masses to undergo malignant transformation, rupture or torsion. Furthermore they may undergo changes in volume and/or morphology that may or may not predict any of these behaviours. To date, no research has rigorously investigated the long-term behaviour of such masses. Consequently, there are no evidence-based guidelines on the optimal management of the majority of adnexal tumours. It is therefore not surprising that clinical practice is highly variable, with some clinicians preferring to operate on virtually any mass. When a clinician decides not to operate, the time intervals selected for follow up scans is often arbitrarily chosen. On the other hand, we do have some convincing data to suggest that simple cysts are rarely malignant and so it is generally thought that operating on these common tumours is probably not necessary and simply increases costs and morbidity. Developing new insights into the natural history of benign looking conservatively managed ovarian masses would potentially change the management of thousands of women, by avoiding surgery or even further surveillance for some and detecting cancer earlier or even preventing it for others.

In this international multicentre study IOTA phase 5 we aim to develop the optimal evidence-based algorithm for the management of all adnexal tumours in order to improve the detection of ovarian cancer while at the same time reducing the number of unnecessary operations. At least three thousand patients with an adnexal mass will undergo an ultrasound examination and if no operation is needed they will be followed up for at least 5 years. At each visit the investigator will assess the tumour and decide whether surgery is necessary based on the available information and local protocols. Survival and logistic regression analysis will be used to develop decision aids to assist clinicians in making decisions regarding surgery and follow up.

Relation with other IOTA studies

The **IOTA study (International Ovarian Tumour Analysis)** is a multicentre collaborative project for the pre-operative characterisation of ovarian tumours..

IOTA phase 1: The **first phase** of IOTA was conducted between 1999 and 2002. Several new mathematical models were developed based on the prospectively collected data of 1066 patients with a persisting adnexal tumour from 9 European centres (1). Between 2002 and 2005 three centres continued the prospective collection in order to be able to perform an internal validation of mathematical models developed in IOTA phase 1. In this so-called IOTA **phase 1b** study a dataset of 507 new patients was prospectively collected in 3 out of the 9 original IOTA centres (2). All models proved to perform excellently with areas under the ROC curves of more than 0.94.

IOTA phase 2: The **second phase** of IOTA consisted of an external validation of the models and this was conducted between 2005 and 2007. The diagnostic algorithms were prospectively validated on 1938 patients with adnexal tumours in 19 centres in Belgium, Italy, UK, Sweden, Poland, Czech Republic, Canada, and China (3). A first analysis showed that overall performance of the logistic regression models was excellent (area under the ROC curve 0.94). We concluded that a subgroup of "uncertain" tumours needs a reliable second stage test in order to help even experienced ultrasound examiners.

IOTA phase 3: The **third phase** of the IOTA study started in 2010.

The aim was to validate the added value of mathematical models as new diagnostic tool in the prediction of ovarian cancer in clinical practice in centres that were involved in IOTA phase 1 or 2. It is a temporal validation of IOTA mathematical models as a first stage examination. However in cases where the prediction is unreliable, we aim to further improve the predictive performance of this diagnostic tool with second stage tests, such as new sets of tumor markers, proteomics and three-dimensional Power Doppler ultrasonography.

IOTA phase 4: Randomised controlled trial in 7 London hospitals. Clinical implementation of IOTA logistic regression models LR2 vs. the routinely used Risk of Malignancy Index. Assessment of efficacy, referral pattern and costs.

INTRODUCTION

There is very little evidence on which to base a recommendation on how apparently benign looking adnexal masses should be managed. Because the natural history of such adnexal masses is not known, and because of the fear of "missing" ovarian cancer, many adnexal masses are currently surgically removed, even if they do not manifest any signs of malignancy. This is not optimal, because every surgical procedure is associated with risks of both short-term and long-term complications, for example pulmonary embolism, deep vein thrombosis, and bowel perforation or obstruction (4). Furthermore we do not know if benign ovarian lesions impact on fertility, although we know that surgery on ovaries may cause adhesions, which in turn may cause infertility, chronic pelvic pain and bowel obstruction. We do not know the frequency of these complications, nor do we know how often benign adnexal masses are associated with complications such as torsion if they are not removed. There is some evidence, however, that expectant management of presumed ovarian dermoid cysts less than 6 cm is safe and does not seem to interfere with

pregnancy or delivery (5-7). The available data also suggest that expectant management of simple cysts less than 5 cm in post-menopausal women is a safe strategy (8,9).

On the other hand ovarian cancer is associated with a high mortality rate and significant morbidity. It is the fifth leading cause of cancer-related deaths (10). The lifetime risk of developing ovarian cancer is around 1 in 50 to 1 in 70 (11,12). Every year more than 200,000 new cases are diagnosed worldwide. The disease has a poor prognosis, with five-year relative survival strongly depending on disease stage (12,13). For example, Cancer Research UK reports survival rates of 73% for stage I versus 16% for stage IV ovarian cancer.

New diagnostic strategies to decrease mortality are needed, as treatment advances have not decreased mortality over the past 20 years (14). Effective screening programmes may help, but current candidate tests remain unsatisfactory (15). A crucial issue is that ovarian cancer is typically asymptomatic in its early stages. Screening algorithms have generally resulted in high sensitivity at the cost of a large number of false positives. As a result a large number of surgical interventions are made in order to find relatively few cancers. However although one study has suggested that there is no benefit in removing benign ovarian tumors (16), there are no conclusive data to inform us regarding the long term behaviour of presumed benign ovarian cysts left in situ. Should benign cysts have malignant potential, then a policy of removing such masses may have a significant impact on mortality from this disease.

Research directed towards the use of diagnostic tests and models to predict malignancy in ovarian tumours has focused on masses that have been subsequently surgically removed in order to provide a clear histological end point. Clinicians decide whether to operate on an ovarian mass depending on a number of factors. These may include the subjective characterisation of the mass using ultrasound, the use of simple models such as the risk of malignancy index, the age of the patient, the serum CA 125 level and the presence or absence of symptoms such as pain. The management of cysts that are not removed surgically is not evidence based and often subject to wide variation. In the absence of rigorous follow up data, we do not know how many false negative results for cancer are associated with these cysts, or if they sometimes undergo malignant transformation. We will only gain this knowledge by long term systematic follow up of a large cohort of ovarian cysts.

A number of studies have focused on the prediction of malignancy in surgically removed masses (for overviews, see references 2,17-19). There is strong scientific evidence that subjective evaluation of a mass using ultrasound by an experienced examiner is a very good method for discriminating between benign and malignant adnexal masses (2,3,20-21), and that a correct histological diagnosis can be suggested on the basis of ultrasound findings in many cases (22,23). We have previously established the International Ovarian Tumor Analysis (IOTA) group to develop and validate prediction models based on large, multi-centre datasets with standardised definitions and data collection procedures (25). The aim was to develop robust models to predict malignancy that performed well, and were widely generalisable. In doing so we aimed to overcome the shortcomings of earlier studies such as small sample sizes, single centre recruitment, and lack of standardised data collection. These models (1, 25-27) successfully passed temporal and external validation (2,3,28). Following these validation studies, we selected two logistic regression models for further study. The first (LR1) is a model with 12 predictors, the second (LR2) contains only six predictors. Even in postmenopausal women conservative management and sonographic follow up of incidental unilocular and multilocular cysts <7cm may be a valuable option (29).

OBJECTIVES

The general aim of this study is the development of the optimal algorithm for the management of all adnexal masses. This can be broken down into different specific objectives: 1) to study the occurrence of complications such as rupture, torsion, or malignancy in patients with benign looking conservatively treated masses; 2) to test the published IOTA diagnostic models for predicting that a mass is malignant at first visit or benign (either on the basis of histology following surgery or by the absence of malignant features on an ultrasound scan one year after the initial visit), and to predict complications (e.g. ocurrence of malignancy and other) during long-term follow-up; 3) to investigate factors that may be related to the need for surgery during long-term follow up; 4) to study the natural history of conservatively treated benign looking masses and to establish descriptive curves of the longitudinal changes seen in parameters from conservatively managed benign tumors (e.g. change in diameter, size of any solid component, number of papillations, or color score). We hope these curves will allow us to determine if any particular growth pattern is associated with complications or malignancy

Related to these four objectives, we aim to carry out the following analyses: 1) descriptive analysis of complications overall and by participating center (anonymised) and overall Kaplan-Meier curves for the need for surgery among benign looking masses; 2) estimation of discriminatory ability of LR1 for malignancy at the initial visit using the c-index and ROC curves and for complications during long-term follow-up using the hazard ratio and c-index within the context of survival analysis; 3) survival analysis to investigate predictors of the need for surgery during long-term follow-up of non-operated masses; 4) the development of longitudinal curves of the changes seen in the characteristics of non-operated masses using longitudinal analysis techniques such as mixed models and functional linear discriminant analysis (FLDA).

METHODS

Study design

International multicenter prospective observational cohort study

Eligible for inclusion

- Any woman at least 18 years old with an adnexal mass.
- Any mass with benign ultrasound morphology may be suitable for conservative management.
- Pregnant patients can be included, but their data will be analysed separately.

Exclusion Criteria

- Cysts that are deemed to be clearly physiological and less than 3 cm in maximum diameter are not eligible for inclusion.
- Any cyst with features of malignancy is excluded from the conservative management
- The denial or withdrawal of oral informed consent

Official approval by the Ethics Committee

The multicentre project IOTA phase 5 will be submitted to the Ethics Committee of the University Hospitals Leuven as main investigating centre as well as in each participating centre.
The study will be performed in accordance with generally accepted standards of Good Clinical Practice and the investigators will adhere to all applicable laws and regulations governing the conduct of clinical trials, including but not limited to the ICH Harmonized Tripartite Guidelines for Good Clinical Practice and the Declaration of Helsinki (2008).

Insurance policy

This multicentre international study is initiated by the University Hospitals Leuven, Belgium. Each participating centre outside Belgium is fully responsible for patient care within its own hospital in agreement with local laws. Each centre is also responsible for all legal aspects of patient care and for its own insurance for all matters related to this study.

Financial Support

The IOTA phase 3 project is supported by an Applied Biomedical Research grant (Toegepast Biomedisch Onderzoek, TBM) from the Flanders Institute for Scientific and Technological Research: IWT Flanders, Belgium (IWT-TBM 070706). This grant covers costs of central data collection, proteomic analysis, analysis of new tumour markers and statistical analyses. For IOTA phase 5 we received a research grant for a doctoral researcher by the Flemish Fund for Scientific Research (FWO Vlaanderen 06260, IOTA5).
There is no financial compensation for principal investigators nor patients.

Definition of benign ultrasound morphology

This is defined on the basis of subjective assessment of ultrasound findings by an experienced ultrasound examiner. Only lesions where the ultrasound examiner is certain or almost certain that the lesion is benign can be managed conservatively. The management of benign masses will be decided according to local protocols.

Number of study patients and recruitment period

This is an observational study and therefore a sample size cannot be calculated. We aim to collect at least 3000 women with an adnexal mass and at least 1000 women with an adnexal mass managed conservatively. We plan an initial recruitment period of eighteen months. Patients will be followed up for at least 5 years, unless surgical intervention is necessary.

Follow-up

Ultrasound (and clinical) follow up will be organised by the ultrasound examiner who entered the patient into the study. Follow-up will be after 3 months (maximal range 1-4 months), 6 months (maximal range 4-8 months) and then every 12 months (maximal range 10-14 months).

Although measurement of serum CA 125 levels is encouraged, it is not mandatory for inclusion in the study. If CA125 is measured it should be recorded in the study screen and preferably measured on each visit.

Duration of follow-up

A yearly analysis will be carried out in order to evaluate acute complications. The duration of follow up will be for at least 5 years and is not limited as long as the patient is compliant with the study and the study is ongoing.

Departments (e.g. radiology departments) that are not involved with clinical decision making about plans for follow up or surgery cannot participate in the full IOTA 5 study. In these centres data can be prospectively collected as an observational study. Only patients with appropriate outcome measures (i.e. follow up ultrasonography after one or more years or patients with complete details of clinical history or surgical procedures) will be included in any statistical analysis.

Collection of clinical data

Family history: Number of first degree relatives with ovarian cancer (0-...)

Medical history: Personal history of ovarian cancer and breast cancer

Age (years)
Previous hysterectomy (yes/no)
Previous oophorectomy (yes/no)
Contraception (drop down list) (None/oral combined contraceptive pill/progestogen only pill/ patch/vaginal ring/Mirena coil/copper IUD)
Hormonal therapy (yes, no).
Is the patient currently wishing to conceive? (yes/no)
Menopausal status (pre- or postmenopausal)
History of subfertility? Yes/no
History of ovarian stimulation for subfertility? Yes/no

<u>For ALL patients before menopause two extra questions pop up:</u>
Patient is currently pregnant? (No/Yes)
Patient became pregnant during the last year? (No/Yes)

- If Yes: Outcome of pregnancy (it should be possible to enter more than one date, should there be more than one pregnancy during follow-up):
  - Ongoing pregnancy
  - Miscarriage; date: …
  - Ectopic; date: …
  - Termination; date: …
  - Delivery; date: …
  - Complications from the lesion during pregnancy? No/Yes (pop up list):
    - Acute pain
    - Chronic pain
    - Suspected torsion

        - Infection
        - Haemorrhage related to the cyst
        - Cyst rupture
        - Required surgery
        - Other, please specify: ….
    - Complications from the lesion during delivery? No/Yes (pop up list):
        - Acute pain
        - Suspected torsion
        - Haemorrhage
        - Cyst rupture
        - Obstructed labour
        - Malpresentation (e.g. breech or unstable lie)
        - Other, please specify: ….….

Ultrasound examination

A standardized ultrasound examination following the IOTA protocol is carried out.
All ultrasound variables are included in the dedicated software. In the database 0 always means NO and 1 always means YES.
The adnexal lesion is that part of an ovary or of an adnexal mass that is judged by ultrasonography to be not consistent with normal physiology. This can be a persistent unilocular cyst, surrounded by normal looking ovarian stroma with some follicles. In this case the whole ovary containing the cyst is the ‘ovary’, whereas the unilocular cyst is the ‘lesion’. Both are measured and the cyst is described as being ‘unilocular’ and not ‘unilocular-solid’. In other cases the lesion is separate from the ovary (e.g. hydrosalpinx). Again, both ovary and lesion are measured separately. In other cases no normal ovarian stroma is seen. In these cases the lesion and the ovary are undistinguishable and the measurement of lesion and ovary will be the same.

Measurements (in mm): The ovary in two perpendicular planes
The lesion in two perpendicular planes
The volume of the tumor is calculated from the three diameters in two perpendicular planes

- The presence of ascites (*i.e.fluid outside* the pouch of Douglas) is noted (yes/no).
- Fluid in the pouch of Douglas is measured in the sagittal plane (the largest anteroposterior diameter is given).
  (see Figure)

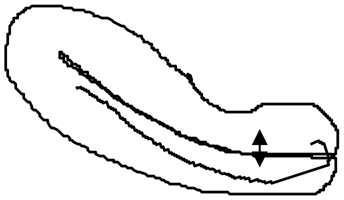

- An incomplete septum (as seen in hydrosalpinges) is defined as a thin strand of tissue running across the cyst cavity from one internal surface to the contralateral side, but is not complete in some scanning planes. If a cyst only has incomplete septa, it is unilocular, despite the fact that in certain sections the cyst appears to be multilocular.
- Solid means echogenicity suggesting the presence of tissue (e.g. the myometrium, the ovarian stroma, myomas, fibromas). Blood clots and the presence of solid tissue can be distinguished by looking for internal movement when gently pushing the structure with the transducer. The presence of blood flow (with the appropriate color Doppler settings) is diagnostic for solid tissue. The absence of flow is not definitive . In cases of doubt the lesion should be classified as solid.

- Solid papillary projections are defined as any solid projections into the cyst cavity from the cyst wall greater than or equal to 3 mm in height

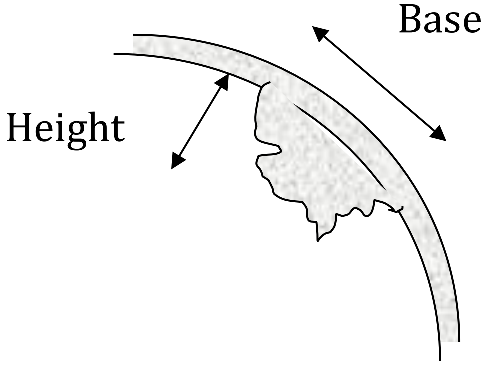

If it is unsure whether solid papillary projections or an incomplete septum are present, the ‘worse case scenario’ is used. E.g. ‘cogwheel excrescences’ and ‘beads-on-a-string’ (as seen in hydrosalpinges) should be classified as papillary excrescences if their height is greater than or equal to 3 mm. The ‘white ball’ in a dermoid (i.e. Rokitansky node), should not be classified as a solid papillary projection.
The ‘sludge’ on the internal walls of endometriotic cysts is not regarded as a papillary projection. In these cases the internal walls are usually ‘irregular’.

- The number of separate papillary projections is noted (1/2/3/more).
- The presence of flow within some of these projections is noted (yes/no).
- Solid papillary projections are described as being ‘smooth’ or ‘irregular’ (e.g. cauliflower-like).

In some cases it is difficult to judge whether it is a papillary projection and from which point to measure the projection. In these cases it may be helpful to use an imaginary line as shown in the following schematic drawing:

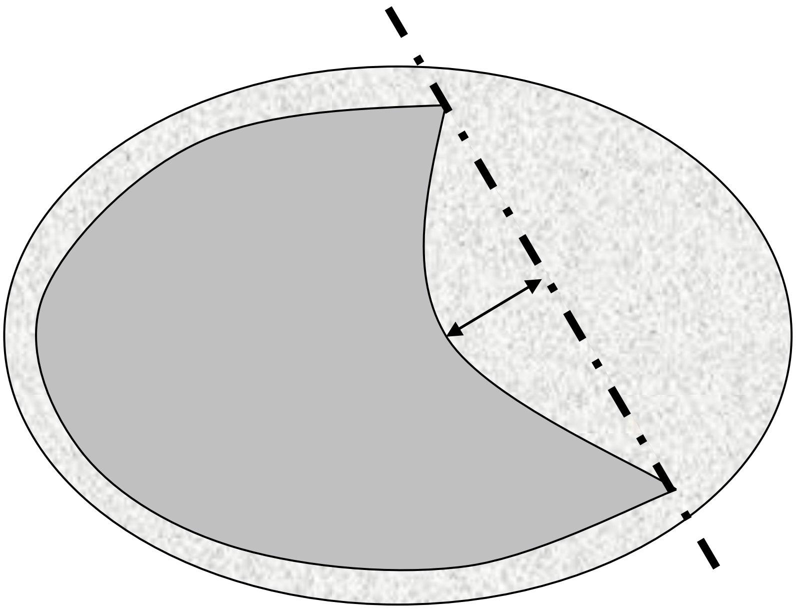

All lesions are qualitatively classified into one of 5 categories:

1. <u>unilocular (a unilocular cyst without septa and without solid parts or papillary structures). Normal ovarian stroma is not regarded</u> as ‘solid’ (e.g. a peritoneal cyst, containing a normal ovary, is unilocular and not unilocular-solid).

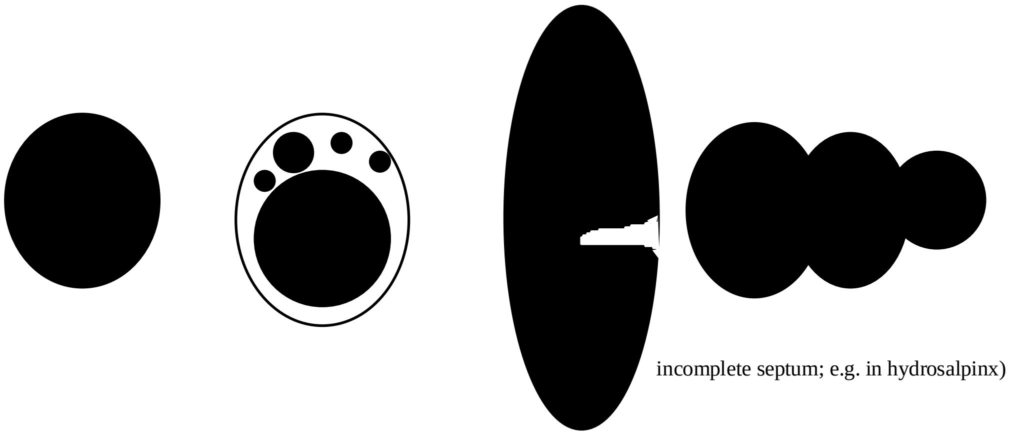

2. <u>unilocular cyst with solid component</u> (a unilocular cyst with a measurable solid component or at least one papillary structure). This category may include pyo- or hydrosalpinges with the so-called ‘beads-on-a-string’ or ‘cogwheel’ appearance if $\geq$ 3 mm. If the solid part contains very small cysts the mass might be unilocular-solid (see below).

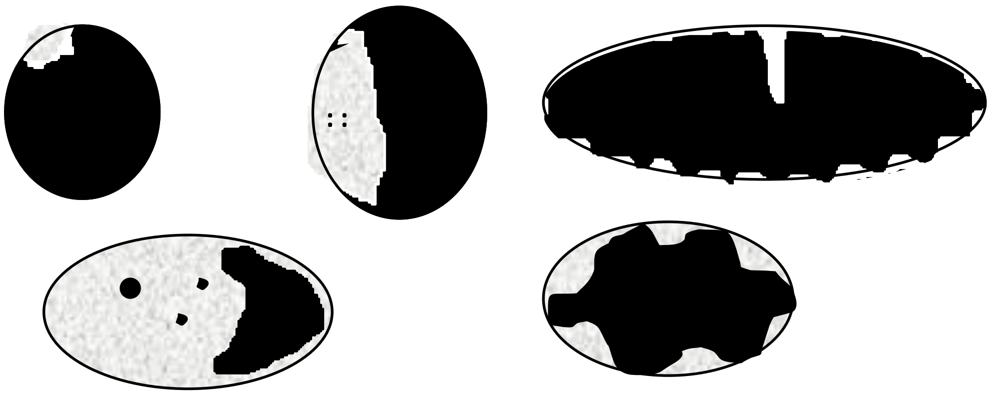

3. <u>multilocular</u> (a cyst with at least one septum but no measurable solid components or papillary projections). The ‘lesion’ is measured as indicated by the arrows.

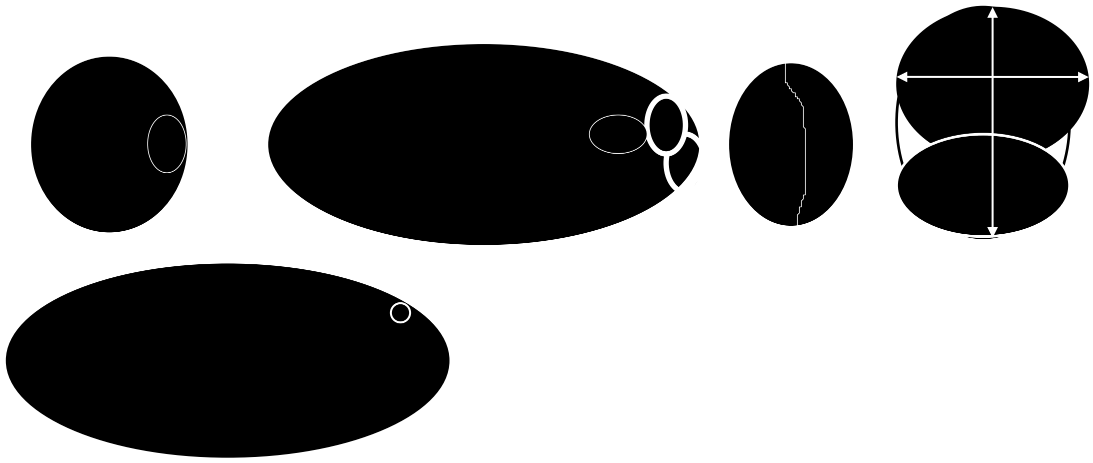

4. <u>multilocular with solid component</u> (a multilocular cyst with a measurable solid component or at least one papillary structure)

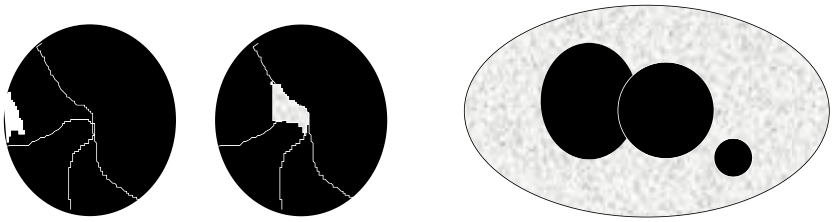

5. <u>solid</u> (a tumour where the solid components comprise 80% or more of the tumour when assessed in a two-dimensional section).

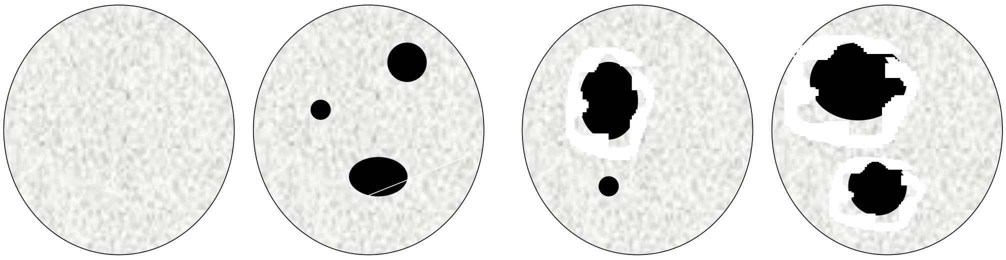

(solid tumour with an irregular cyst wall)

A solid tumour may contain papillary projections protruding into the small cysts.

Quantitative assessment of morphology

- In cystic-solid tumours the <u>largest solid component</u> is measured separately (in three perpendicular planes). The solid component is noted as being smooth or irregular (e.g. cauliflower-like). In some cases a solid papillary projection is the largest solid component and thus the papillary projection is recorded <u>both</u> as papillary projection and as solid component.
- The internal wall is also noted as being <u>smooth or irregular</u>.

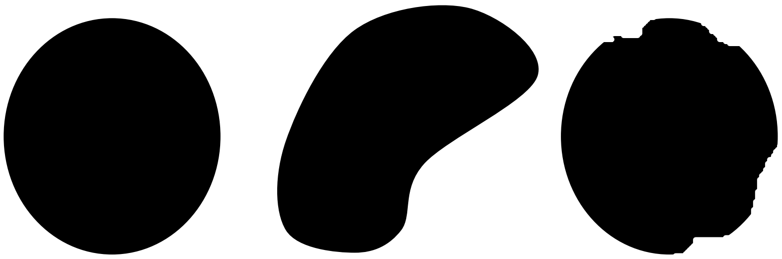

Smooth Smooth Irregular

If there is a solid papillary projection, then the wall is irregular by definition.

- The external wall of tumors are not examined unless they are solid.
- In cases of solid tumours the description of the internal wall being smooth or irregular is usually not applicable but the outline of the tumour is described as smooth or irregular.
- If there is any irregularity in either the inner wall of any cyst or in the outer wall of a solid tumour or on the surface or echogenicity of a solid component, the lesion is described as ‘irregular’.

- The dominant feature of the cystic contents is described as anechoic (black), low-level echogenic (homogeneous low level echogenic as seen in mucinous tumours), ‘ground glass’ appearance (homogeneously dispersed echogenic cystic contents, as often seen in endometriotic cysts), hemorrhagic (with internal thread-like structures, representing fibrin strands; it is possible to describe the echogenicity as star-shaped, cobweb-like or jelly-like) or mixed echogenic (as often seen in teratomas) (see images attached).

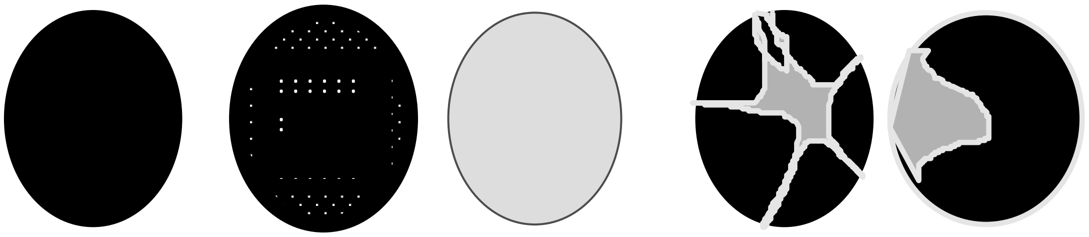

Anechoic Low level Ground glass Hemorrhagic Hemorrhagic

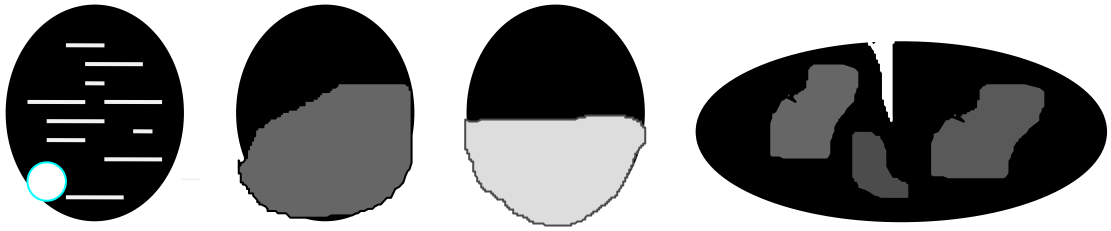

Mixed Mixed (old blood-fluid level or fat-fluid level) Mixed (e.g. abscess)

- The presence of acoustic shadows, defined as loss of acoustic echo behind a sound-absorbing structure, is noted as well. Solid tumours are identified by the appearance of the internal texture, by the absence of internal movement when moving the transducer or by colour Doppler imaging (presence of central flow).
- In solid tumours the dominant feature of any cystic contents is described only if it can be assessed.
- ‘Ovarian crescent sign’, defined as the presence of normal ovarian tissue adjacent to an adnexal tumour. (“absent” or “present”, mandatory new variable for phase 3 and 5)
- Ultrasound evidence of metastases (e.g. “omental cake” or peritoneal tumoural implants). (“absent” or “present”, mandatory new variable for phase 3 and 5)

Colour Doppler imaging and blood flow indices

Subsequently, the entire tumor is surveyed by CDI. The power, gain and pulse repetition frequency are initially adjusted for maximum sensitivity of low blood flow states. The lowest velocity signals are filtered out by gradually increasing the pulse repetition frequency and flow analysis is concentrated on the highest velocity signals. A subjective semiquantitative assessment of the amount of blood flow (area and colour scale) within the septa, cyst walls, or solid tumor areas is made: a score of 1 is given when no blood flow can be found in the lesion; a score of 2 is given when only a small amount of flow can be detected; 3 is given when moderate flow is present and 4 is given when the adnexal mass appears highly vascular with marked blood flow using colour Doppler (abundant flow). This colour score refers only to the colour Doppler image and not to Doppler shift spectrum. It is given for the tumour as a whole (not for a solid part or a septum only, but for the whole tumour). Multiple photographic prints are made of relevant structures and Doppler signals.

Quality control

Several informative images or volumes of all adnexal masses should be made. Preferably, these are stored digitally. Photographs or video are acceptable as well.

Subjective assessment

After ultrasonographic examination of the mass the investigator gives his subjective assessment of the mass:
A: Malignant or benign or borderline?
B: Probability of malignancy: 1 = benign
(=level of certainty) 2 = probably benign
3 = uncertain
4 = probably malignant
5 = malignant
C: Self impression: presumed histological diagnosis (e.g. dermoid, serous cystadenoma, endometrioma, abscess…)

Surgical intervention

Surgery is performed according to local protocols. The reason for surgery, e.g. symptoms (pain, discomfort or pressure symptoms), raised serum CA125 levels or changes in the morphology or volume of the mass is recorded in the study screen.

Study screen

An astraia study screen will be used which will permit the entry of **multiple scans** per patient.

All centres will receive the **IOTA 5 study screen**.
At initial set up centres can choose between the two options:

1. Full IOTA 5 study (with planning of appropriate follow up and conservative management whenever feasible).
2. Observational study only (e.g. in radiology departments that are not involved in management decisions)

Recorded variables (entered in the **astraia study screen**).

Patient data
(click one option from list below)

- New patient with diagnosis of adnexal mass
- New patient who was already in follow up in your centre for adnexal mass before she was enrolled to the IOTA 5 study. How many months in follow up? …
- Follow up scan of patient that is already enrolled to the IOTA 5 study before

Ultrasound

- Spontaneous resolution of the adnexal mass (no further details are entered)
- Adnexal mass present (fill in all variables below)

- 12 variables described in LR 1:
  - Age
  - personal history of ovarian cancer
  - personal history of breast cancer
  - Max diameter of lesion
  - Max diameter of solid component
  - Presence of ascites
  - Presence of blood flow within papillary projection
  - Irregular internal cyst walls
  - Presence of a purely solid tumour
  - Colour score (1/2/3/4)
  - Presence of acoustic shadows

- Current hormonal therapy
- Presence of pain during the examination

- as well as simple rules, RMI and other variables:
  - Type of tumour (unilocular/unilocular-solid/multilocular /multiloc-solid/solid)
  - Ovarian crescent sign
  - Cyst content
  - Incomplete septum
  - Mobility: mobile/reduced mobility/completely fixed
  - Number of locules
  - Number of papillations (0/1/2/3/more)
  - Size of ovary
  - Bilateral tumour
  - Evidence of metastases
  - Menopausal status: premenopausal/postmenopausal
  - Serum CA 125 result (not mandatory)

- Symptoms during the last year before ultrasound scan (multiple options are possible)
  - Pelvic pain
  - Postmenopausal bleeding
  - increased abdominal size
  - persistent abdominal distention (bloating)
  - appetite loss
  - constipation
  - diarrhoea
  - urinary urgency
  - urinary frequency
  - weight changes
  - dyspareunia
  - Other: please specify: ….

- *For centres participating at the full IOTA 5 study only:* Suggested management recorded by examiner: "What type of management do you propose for this patient based on ultrasound and clinical data?"
  - Conservative management without follow up
  - Conservative management with follow up as specified in the protocol
  - Surgery by a gynaecologist or general surgeon
  - Surgery by an oncological surgeon

Current status of the patient (this new tab should come before the tab "Histology"). A fixed query could be made to automatically ask the investigator about the status of all patients that were not rescanned 55 weeks after their previous scan as soon as the investigator opens the IOTA 5 study screen.

- Lost to follow up (no other pop-up)
- Patient stopped participating to the study (please specify why : …..) (no other pop-up)
- Patient withdrew her consent (data cannot be used for statistical analysis and no reason is asked) (no other pop-up)
- Surgery performed (pop-up of fields about operation below)

  If Surgery

  - Date of operation:
  - Type of operation: cystectomy or oophorectomy or staging etc
    - Laparotomy with vertical incision
    - Laparotomy with horizontal incision

- Operative laparoscopy
    - Diagnostic laparoscopy
    - Primary chemotherapy
- Indication for operation (more than one possibility may be thicked)
    - Suspicion of malignancy based on ultrasound
    - Suspicion of malignancy based on other information if so what?
    - Malignancy cannot be excluded
    - Acute pain
    - Chronic pain
    - Suspected torsion
    - Fertility concerns
    - Patient request
    - Increase in size of the tumour
    - Change in morphology of the tumour
    - Increase in CA 125 level
    - Indicated by other imaging technique (CT, MRI…)
    - Other doctor recommended operation. Please specify the reason: …
    - “en passent” removal of the mass when patient was operated for another indication
    - Other: Specify: ….
- Findings at operation (more than one option may be ticked)
    - No complications of the tumour
    - Torsion
    - Rupture
    - Inflammation/Infection
    - Adhesions
    - Bleeding from tumour
    - Metastatic cancer
    - Other complications of tumour: specify: ….
    - Other non-gynaecological pathology (e.g. appendicitis): specify: ….
- Complications during operation (within one week of surgery). (more than one option may be ticked) :
    - Conversion from laparoscopy to laparotomy
    - Bowel perforation
    - Bleeding requiring transfusion
    - Embolism, deep venous thrombosis
    - Wound Infection
    - Peritonitis
    - Other: Specify: ….
- Histological diagnosis (pop up list as before) with open text area (“Details”)

<u>If follow up examination:</u>

- Complications of adnexal mass: no /yes
- If yes : date of first complication: ….
- And pop up list (you can select more than one):
    - Acute pain
    - Chronic pain
    - Suspected torsion
    - Infection
    - Haemorrhage
    - Rupture
    - Other, please specify: ….
- Death: date

- Death directly or indirectly relatd to adnexal mass?
- cause of death (
- Autopsy findings: specify: ….

Not needed for IOTA phase 5: second stage tests

Consent / information leaflet

Information leaflets are at the discretion of the participating centres.
Approval of the local Ethical Committee for clinical studies is necessary.

Serum tumour markers

Serum CA 125 measurements or other tumour markers are performed locally, using a CA 125 II immunoradiometric assay

Tissue collection

Preferably the whole tumour should be removed. However, representative biopsies may be sufficient (e.g. in advanced ovarian cancer or endometrioma).

Tumour classification

Tumours are classified according to the criteria recommended by the International Federation of Gynecology and Obstetrics (FIGO). In malignant tumours the degree of differentiation is included.

Statistical analysis

For the first objective, a descriptive analysis of complications on all data and stratified for participating center (anonymized) will be performed, as well as an overall Kaplan-Meier curve of 'complication free survival'. Complication free survival is defined as the time to the need for surgery during long-term follow-up. Patients that did not need surgery at the end of the follow-up period are right censored at the time of the last examination.
For the second objective, the discriminatory ability of LR1 and the polytomous model to detect malignancy at the initial visit will be assessed. To this end, the logit of the risk of malignancy given by LR1, logit(LR1), is used to predict malignancy using logistic regression, and the performance assessed with the odds ratio, the c-index and a ROC curve. Linearity of the effect of logit(LR1) will be assessed using spline functions. This analysis is planned one year after the end of the inclusion phase of the trial. Non-operated masses will be classified as benign at the initial visit if there is an absence of a clinical diagnosis of cancer after 1 year of follow up. In addition, the ability of LR1 to predict complications during long-term follow-up will be assessed. This will be done using Cox proportional hazards regression with the logit(LR1) as predictor of complication free survival. Performance will be assessed using the hazard ratio and the c-index within the context of survival analysis. Linearity of the effect of logit(LR1) will be investigated using Schoenfeld residuals.
For the third objective, a multivariable survival analysis will be undertaken using Cox proportional hazards regression or more complex alternatives based on support vector machines (30). The models will be penalized to prevent overfitting, given that not enough events (i.e., patients that need surgery during long term follow-up) are expected for the number of available predictor variables. Internal validation will be assessed through bootstrapping rather than a split of the data in training and test sets (31).
For the fourth objective, longitudinal normative curves will be derived of the changes seen in the characteristics of non-operated masses. Longitudinal analysis techniques such as mixed models, longitudinal support vector machines, and functional linear discriminant analysis (FLDA) (32-34).

Study supervision

Central supervision: the Steering Committee is responsible for the protocol, quality control, interim analyses of the data and final analysis and reporting of the study.

Local supervision: the Principal Investigators are responsible for the data collection in their centres.
Dirk Timmerman is responsible for the co-ordination of the overall IOTA project and the contact between the centres.
Sabine Van Huffel and Bart De Moor are responsible for the data management and the development of new algorithms, in collaboration with Ben Van Calster, Lieveke Ameye and Kirsten Van Hoorde.

Publication policy

The steering committee is responsible for publication of the data in scientific journals. As such the members are co-authors in all resulting clinically relevant papers, to which they made significant contributions. By the time of the final analysis the principal investigators have to have contributed at least 50 cases to the study. They are co-authors, according to the number of patients they contributed to the study (depending on the journal's restriction of the number of co-authors) on condition that they contribute to writing the papers and read and approve the final version.
Purely mathematical papers without clinical relevance related to the study data are published by S. Van Huffel, B. De Moor and co-workers at ESAT with reference to the IOTA group and the inclusion of as many as possible of the clinical contributors.
The Katholieke Universiteit Leuven represented by its department K.U.LEUVEN RESEARCH & DEVELOPMENT, having its office in 3000 Leuven, Minderbroedersstraat 8A – box 5105, Belgium, VAT number BE 419.052.173 holds intellectual property rights that might result from the IOTA project.

REFERENCES (FOR THE PROTOCOL ONLY)

1. Timmerman D, Testa AC, Bourne T, et al. Logistic regression model to distinguish between the benign and malignant adnexal mass before surgery: a multicenter study by the International Ovarian Tumor Analysis Group. J Clin Oncol 2005; 23:8794-8801.
2. Van Holsbeke C, Van Calster B, Testa AC, et al. Prospective internal validation of mathematical models to predict malignancy in adnexal masses: results from the Internation Ovarian Tumor Analysis study. Clin Cancer Res 2009; 15:684-691.
3. Timmerman D, Van Calster B, Testa AC, et al. Ovarian cancer prediction in adnexal masses using ultrasound based logistic regression models: a temporal and external validation study by the IOTA group. Ultrasound Obstet Gynecol 2010; 36(2):226-34.
4. Menon U, Gentry-Maharaj A, Hallett R, et al. Sensitivity and specificity of multimodal and ultrasound screening for ovarian cancer, and stage distribution of detected cancers: results of the prevalence screen of the UK Collaborative Trial of Ovarian Cancer Screening(UKCTOCS). Lancet Oncol. 2009 Apr;10(4):327-40.
5. Hoo WL, Yazbek J, Holland T, Mavrelos D, Tong EN, Jurkovic D. Expectant management of ultrasonically diagnosed ovarian dermoid cysts: is it possible to predict outcome? Ultrasound Obstet Gynecol. 2010 Aug;36(2):235-40.
6. Caspi B, Appelman Z, Rabinerson D, Zalel Y, Tulandi T, Shoham Z. The growth pattern of ovarian dermoid cysts: a prospective study in premenopausal and postmenopausal women. Fertil Steril. 1997 Sep;68(3):501-5. PubMed PMID: 9314922.
7. Caspi B, Levi R, Appelman Z, Rabinerson D, Goldman G, Hagay Z. Conservative management of ovarian cystic teratoma during pregnancy and labor. Am J Obstet Gynecol. 2000 Mar;182(3):503-5. PubMed PMID: 10739498.
8. Valentin L, Akrawi D. The natural history of adnexal cysts incidentally detected at transvaginal ultrasound examination in postmenopausal women. Ultrasound Obstet Gynecol. 2002 Aug;20(2):174-80.
9. Valentin L, Skoog L, Epstein E. Frequency and type of adnexal lesions in autopsy material from postmenopausal women: ultrasound study with histological correlation. Ultrasound Obstet Gynecol. 2003 Sep;22(3):284-9.
10. Jemal A, Siegel R, Ward E, Hao Y, Xu J, Thun MJ. Cancer statistics 2009. CA Cancer J Clin 2009; 59:225-249.
11. Cancer Research UK a. UK ovarian cancer incidence statistics. http://info.cancerresearchuk. org/cancerstats/types/ovary/incidence/ (accessed Oct 7, 2009).
12. Horner MJ, Ries LAG, Krapcho M, et al (eds.) SEER Cancer statistics review 1975-2006, NCI, Bethesda, MD.
13. Cancer Research UK b. UK Ovarian cancer survival statistics. http://info.cancerresearchuk. org/cancerstats/types/ovary/survival/ (accessed Oct 7, 2009).
14. Engel J, Eckel R, Schubert-Fritschle G, et al. Moderate progress for ovarian cancer in the last 20 years: prolongation of survival, but no improvement in cure rate. Eur J Cancer 2002; 38:2435-45.
15. Vergote I, Amant F, Ameye L, Timmerman D. Screening for ovarian carcinoma: not quite there yet (editorial). Lancet Oncology 2009; 10:308-309.
16. Crayford TJB, Campbell S, Bourne TH, Rawson HJ, Collings WP. Benign ovarian cysts and ovarian cancer: a cohort study with implications for screening. Lancet 2000; 355:1060-1063.
17. Timmerman D. The use of mathematical models to evaluate pelvic masses: can they beat an expert operator? Best Pract Res Clin Obstet Gynaecol 2004; 18:91-104.
18. Van Holsbeke C, Van Calster B, Valentin L, et al. External validation of mathematical models to distinguish between benign and malignant adnexal tumors: a multicenter study by the International Ovarian Tumor Analysis Group, Clin Cancer Res 2007; 13:4440-4447.
19. Geomini P, Kruitwagen R, Bremer GL, Cnossen J, Mol BW. The accuracy of risk scores in prediting ovarian malignancy: a systematic review. Obstet Gynecol 2009; 113:384-394.

20. Valentin L, Jurkovic D, Van Calster B, et al. Adding a single CA 125 measurement to ultrasound imaging performed by an experienced examiner does not improve preoperative discrimination between benign and malignant adnexal masses. Ultrasound Obstet Gynecol. 2009 Sep;34(3):345-54. PubMed PMID: 19585547.
21. Van Calster B, Timmerman D, Bourne T, et al. Discrimination between benign and malignant adnexal masses by specialist ultrasound examination versus serum CA-125. J Natl Cancer Inst. 2007 Nov 21;99(22):1706-14. Epub 2007 Nov 13. PubMed PMID: 18000221.
22. Valentin L. Pattern recognition of pelvic masses by gray-scale ultrasound imaging: the contribution of Doppler ultrasound. Ultrasound Obstet Gynecol. 1999 Nov;14(5):338-47.
23. Sokalska A, Timmerman D, Testa AC, et al. Diagnostic accuracy of transvaginal ultrasound examination for assigning a specific diagnosis to adnexal masses. Ultrasound Obstet Gynecol. 2009 Oct;34(4):462-70.
24. Timmerman D, Valentin L, Bourne TH, Collins WP, Verrelst H, Vergote I. Terms, definitions and measurements to describe the sonographic features of adnexal tumors: a consensus opinion from the International Ovarian Tumor Analysis (IOTA) group. Ultrasound Obstet Gynecol 2000; 16:500-505.
25. Van Calster B, Timmerman D, Lu C, et al. Preoperative diagnosis of ovarian tumors using Bayesian kernel-based methods. Ultrasound Obstet Gynecol 2007; 29:496-504.
26. Van Calster B, Timmerman D, Nabney IT, et al. Using Bayesian neural networks with ARD input selection to detect malignant ovarian masses prior to surgery. Neural Comput Applic 2008; 17:489-500.
27. Ameye L, Valentin L, Testa AC, et al A scoring system to differentiate malignant from benign masses in specific ultrasound-based subgroups of adnexal tumors. Ultrasound Obstet Gynecol 2009; 33:92-101.
28. Van Holsbeke C, Van Calster B, Bourne T, et al. External validation of diagnostic models to predict the risk of malignancy in adnexal masses. Submitted.
29. Leone FP, Crepaldi A, Marciante C, Cetin I. Sonographic follow-up of unilocular >5 cm and multilocular ovarian cysts <7 cm in post-menopausal women: preliminary results. OC21.05. Ultrasound Obstet Gynecol 2011; 38 (Suppl. 1): 40.
30. Van Belle V, Pelckmans K, Suykens JAK, Van Huffel S. Additive survival least squares support vector machines. Stat Med 2010**;** 29:296-308.
31. Steyerberg EW, Harrell FE Jr, Borsboom GJ, Eijkemans MJ, Vergouwe Y, Habbema JDF. J Clin Epidemiol 2001; 54:774-781.
32. Verbeke G, Molenberghs G. Linear mixed models for longitudinal data. Springer: New York, 2000.
33. Luts J, Molenberghs G, Verbeke G, Van Huffel S, Suykens JAK. A mixed effects least squares support vector machine model for classification of longitudinal data. Internal Report 10-249, ESAT-SISTA, K.U.Leuven (Leuven, Belgium), 2010.
34. James GM, Hastie TJ. Functional linear discriminant analysis for irregularly sampled curves. J R Stat Soc B 2001;63:533–550.

Appendix 2 – Telephone survey

SECTION A- Telephone Survey for IOTA 5 patients lost to follow-up

(Please use it ONLY if a complete follow-up in your ultrasound clinic is absolutely not possible)

IOTA 5 patient name and surname______________________(IOTA ID……………………………)

Good morning/afternoon, I am Dr. *(name, surname)*, I am calling you from the Department of Gynaecology and Obstetrics at *(name of the Hospital)* Hospital. I am conducting a telephone research survey on patients enrolled in the IOTA 5 study.
You underwent an ultrasound examination of an ovarian mass in our clinic on the *(DD/MM/YYYY)* and we have already asked you if you agreed to give your consent to take part in our study. As we did not see you again at our ultrasound clinic, I am calling you in order to have some additional information by telephone, regarding possible surgery you underwent because of the ovarian cyst/swelling. Do you have time to talk to me now? *(if the answer is no, ask if you can call another time and which time is suitable)*.

Do you remember about the IOTA 5 study? (Even if the patient remembers the IOTA5 study, please give some explanations)

It is a study on patients with masses in the ovaries or tubes. The aim is to collect detailed information on the ultrasound features of the ovarian mass and what happens to patients with a mass to understand the natural history of these masses: if they disappear spontaneously, if they grow, if they change their appearance, if they start to cause symptoms, etcetera. One aim of the study is to find out if the ultrasound features can predict what will happen in the future to choose the best management.

I would like to ask you if you are willing to answer some questions that are important for the study. All the information I receive from you by phone will be analysed together with the information we already have in our database. It is not possible to identify you in the database. Your participation in this survey is completely voluntary, as well as your participation to the whole study. This means that you do not have to participate unless you do not want to. Whether you choose to participate or not will not affect your current or future medical care in any way.
Do you agree to take part to this telephone survey for the IOTA 5 study?
°yes °no

(If no, thank the patient for her time and end the call and register it in the IOTA5 study screen "withdrawal of consent").
(If yes, proceed)

Did you undergo any gynecological surgery after the last scan (repeat date of the scan)?
°yes °no

If yes:

- In which hospital did you undergo surgery? ________________________________
- When? (DD/MM/YYYY)____________________________
- Do you remember the name of the surgeon/gynaecologist who operated on you or took care of you when you underwent surgery? State name here_________________________________
  (Please ask the patients any other useful information in order to retrieve all her data in another hospital, according to your National Health System).

Do you consent that we contact the hospital where you were operated on to retrieve information on the surgery and the type of ovarian mass that was removed according to the microscopical examination of the mass?
°yes °no
- If the patient DID NOT UNDERGO SURGERY, please ask to her if she still would like to continue to take part to the IOTA 5 study. If she is willing, the next ultrasound exam in your centre should be planned (you can do it during the call, or the patient can do it afterwards contacting the centre).

If the patient is does NOT want to continue follow-up in IOTA 5, please state the reason (fill in IOTA5 study screen "patient withdrew consent" or "patient stopped participating in the study"→ in this case please explain the reason e.g. patient underwent palliative treatment, patient moved to another city/country etc.)

The survey is now completed.
Thank the patient for her time and end the call.

Patient Name: ________________________________
Date of telephone survey: ____________________
Name and signature of person performing telephone survey: ___________________________

(Then, contact the Centre/Surgeon where the patient was treated: follow SECTION B)

PLEASE FILL IN THE RESULTS OF THE TELEPHONE INTERVIEW IN THE IOTA 5 STUDY SCREEN AND SAVE THE CHANGES.

SECTION B- Telephone Survey for Colleagues who have cared for an IOTA 5 patient

IOTA 5 patient name and surname______________________(IOTA ID……………………………….)

Good morning/afternoon, I am Dr. *(name, surname)*, I am calling you from the Department of Gynaecology and Obstetrics at *(name of the Hospital)* Hospital. I am conducting a telephone research survey on patients enrolled in the IOTA 5 study. It appears that you have cared for one of our IOTA 5 patients that we have so far lost to follow-up and that you therefore can provide us with the information that we need. The patient herself has consented that we contact you to retrieve this information. Do you have time to answer some questions now? *(if the answer is no, ask if you can call another time and which time is suitable)*.

The IOTA 5 study is a study on patients with masses in the ovaries or tubes. The aim is to collect detailed information on the ultrasound features of the ovarian mass and what happens to patients with a mass to understand the natural history of these masses: if they disappear spontaneously, if they grow, if they change their appearance, if they start to cause symptoms, etcetera. One aim of the study is to find out if the ultrasound features can predict what will happen in the future to choose the best management.

We saw Ms. (name, surname of the patient) at our ultrasound clinic the_(date in which you saw the patient last time) and we have had already asked her if she agreed to take part to our study. We called the patient the (date of the call) and she told us that she underwent surgery in your Department. I would like to have some additional details about the surgery. Would it be possible for you to send this information by mail/secure email/fax to us? All the information we will receive from you will be analysed together with the information we already have in the patient's database. The patient has consented to this. It is not possible to identify the patient in the database.

Do you agree?
°yes °no

If yes, we will send you a list of questions (SECTION B1) regarding the surgery and the histological diagnosis. In case filling in all this information is too demanding, I would kindly ask you whether it is possible for you to send me the medical record/s of this patient, which will allow us to complete the missing data. (*collect the email/mail address or fax number according to your country's regulation and send the questionnaire*). *Remember to indicate also a mail address or a fax number where your colleagues can send you the completed questionnaire/the patient's medical record/s, according to their preference).*
Thank the colleague for her/his time and end the call.

If no, ask if it is possible to call someone else in order to have this information, or if it is possible to call the colleague at another moment/day.

If no for any other reasons, thank the colleague for her/his time and end the call. Make a note in the IOTA 5 study screen that it was impossible to retrieve information on surgery and histological outcome. Save the changes.

Patient Name: __________________________________
Doctor's name: ____________________________
Date of telephone contact: _____________________
Name and signature of person performing the telephone contact: _____________________________

PLEASE REMEMBER TO FILL IN THE DATA YOU WILL OBTAIN BY THE QUESTIONNAIRE/PATIENT'S MEDICAL RECORD IN THE IOTA 5 STUDY SCREEN AND SAVE THE CHANGES.

SECTION B1- Questionnaire IOTA 5 patients undergone surgery

Patient name and surname:________________________________(IOTA ID………)
Patient birth date:……………………………………………………..
Please add any other patient information useful to retrieve all data in another hospital, according to your National Health System).

Date of surgery (DD/MM/YYYY):
Surgical approach:

- Diagnostic laparoscopy
- Operative laparoscopy
- Laparotomy with horizontal incision
- Laparotomy with vertical incision
- Primary chemotherapy
- Robotic surgery
- Biopsy followed by neo-adjuvant chemotherapy (NACT)
- Other (specify………………………..)

Surgical procedure:

- Cyst drainage
- Cystectomy
- Salpingo-oophorectomy
- Hysterectomy + BSO
- Debulking surgery
- Adhesiolysis
- Biopsy
- Fertility sparing radical surgery
- Other: specify………

Indication for procedure:

- Suspicion of malignancy based on ultrasound (performed where? Specify......................)
- Suspicion of malignancy based on:
    - Increase in size of tumour
    - Change in morphology of tumour
    - Change in vascularity of tumour
    - Raised serum CA125
    - CT scan findings
    - MRI scan findings
    - Raised HE4
    - Other (please specify…………………………)

- Acute pain
    - Suspected torsion
    - Suspected cyst rupture
- Chronic pain
- Fertility concerns
- Patient request
- Opportunistic removal during other surgery
- Patient complaint other than pain
- Other (please specify……………………………………………….)

Decision to perform surgery made by:

- General gynaecologist
- Gynaecological oncologist
- Ultrasound specialist
- Fertility specialist
- Primary care
- General surgeon

Surgical findings:

- No tumour found
- No complication of the tumour
- Torsion of the dominant mass
- Rupture of dominant mass
- Bleeding from dominant mass
- Inflammation or infection
- Adhesions
- Intra-abdominal spread of disease
- Other (please specify………………………………………..)

Final Histology

- **Benign tumour:**
    - Normal adnexa
    - Simple cyst
    - Functional cyst
    - Haemorragic corpus luteum cyst
    - Endometrioma
    - Teratoma (benign)
    - Fibroma
    - Thecoma
    - Serous cystadenoma
    - Mucinous cystadenoma
    - Serous cystadenofibroma
    - Mucinous cystadenofibroma
    - Inclusion cysts
    - Paraovarian/parasalpingeal cyst
    - Peritoneal pseudocyst
    - Other: ………
- **Rare benign tumour:**
    - Struma ovarii
    - Brenner tumour (benign)

- Schwannoma
- Other: ……..

- **Infectious (acute/chronic):**
    - Hydrosalpinx
    - Abscess
    - Salpingitis
    - Other: ……….
- **Uterine lesion:**
    - Fibroid
    - Subserous adenomyoma
    - Other: ………..
- **Borderline tumours:**
    - Serous borderline
    - Mucinous endocervical borderline
    - Mucinous gastrointestinal borderline
    - Other: ……….
        - ➢ FIGO stage: A B C
            - I
            - II
            - III
            - IV
            - Not known
            - Not applicable
            -
- **Primary invasive malignant tumour:**
    - **Epithelial ovarian cancer**
        - Serous

HGSOC (high grade serous ovarian cancer)

LGSOC (low grade serous ovarian cancer)

- Mucinous
- Endometrioid
- Clear cell
- Small cell carcinoma
- Other: ……….
    - ➢ FIGO stage: A B C
        - I
        - II
        - III
        - IV
        - Not known
        - Not applicable

- **Malignant germ cells tumour of the ovary**
    - Immature teratoma
    - Malignant struma ovarii
    - Dysgerminoma
    - Choriocarcinoma
    - Yolk sac/ Endodermial sinus tumour
    - Other: ……..
        - ➢ FIGO stage: A B C

- I
- II
- III
- IV
- Not known
- Not applicable

- **Stromal and sex-cord tumours of the ovary**
  - Granulosa-adult
  - Granulosa-juvenile
  - Sertoli
  - Sertoli-Leydig
  - Leydig
  - Fibrosarcoma
  - Carcinosarcoma
  - Other:………….
    - FIGO stage: A B C
      - I
      - II
      - III
      - IV
      - Not known
      - Not applicable
- **Tubal cancer**
  - Serous
  - Mucinous
  - Endometrioid
  - Clear cell
  - Small cell carcinoma
  - Other: ………
    - FIGO stage: A B C
      - I
      - II
      - III
      - IV
      - Not known
      - Not applicable
- **Metastatic malignant tumour:**
  - Krukenberg
  - Metastasis from breast cancer
  - Metastasis from gastrointestinal tumour
  - Lymphoma
  - Other:……….
- **Very rare malignant tumour: …………**
  - FIGO stage: A B C
    - I
    - II
    - III
    - IV
    - Not known
    - Not applicable

- Specify……………

Did any **intraoperative complications** occur?

- o No complications
- o Need for conversion from laparoscopy to laparotomy
- o Visceral damage (large or small bowel, bladder, ureter)
- o Major hemorrage requiring transfusion
- o Major vascular incidents
- o Respiratory or heart related events (i.e. gas/pulmonary embolism, cardiac arrest,, or arrhythmias
- o Intra-operative mortality
- o Return to operating theatre
- o Other (please specify……………………………….)

Did any **early (within 30 days) post-operative complications** occur?

°yes °no

If yes, please specify……………………………………..

Did any **late (more than 30 days) post-operative complications occur?**

°yes °no

If yes , please specify…………………………………….

Please, attach copies of the operation report and histological report when you return your questionnaire.

Please return the completed survey/patient's medical record by mail/secure email/fax to the IOTA investigator who contacted you.
Thank you for the time you spent to help the IOTA group in this valuable project!

Appendix 3 – Details on the exclusion of centres

Supplementary Table 1 indicates whether centres were included or excluded from the primary analysis (the 'decision' column). Here, we provide detailed information on this decision. Table A3.1 provides an overview of data for included and excluded centres separately.

Seven centres (Lisbon 2, Cremona, Catania, Paris, Aarschot, Maurepas, Vienna) were excluded because less than 50 patients were recruited. The number of recruited patients could be below 50 because the centre joined the study late, or because the centre did not recruit consecutively. The threshold of 50 is partly arbitrary. It requires centres to recruit at least 16 patients per year on average, which we considered reasonable.

Three centres (Udine, Lisbon, Tampa) were excluded because they did not include all patients but focused on either patients that were operated on without follow-up scans or on patients managed conservatively. Udine focused on patients that were immediately operated on, Lisbon and Tampa focused on patients that were managed conservatively. Our study focuses on consecutive patients irrespective of how they are managed. Inclusion of the centres in Udine, Lisbon and Tampa would introduce selection bias.

One centre (Krakow) was excluded because the centre stopped participation and could not complete follow-up information.

Eight centres (Bologna, Lublin, Prague, Bari, Milan 4, Cairo, Tienen, Beijing) were excluded due to insufficient quality of follow-up information. The IOTA steering committee decided that for a centre to be included it should have good follow-up information in at least 70% of patients for which the initial policy was conservative management. No good follow-up information means that there was no final study outcome (i.e. spontaneous resolution, or histology due to surgery at any time during follow-up) and the last follow-up visit was less than 10 months after the inclusion scan. The threshold of 70% is partly arbitrary but was considered reasonable given the fact that loss to follow-up is unavoidable to some extent. See also Froyman et al (2019).[1]

Table A3.1. Characteristics of included and excluded centres.

	All centres	Included centres	Excluded centres
Centres	36	17	19
Oncology centres	20	9	11
Other centres	16	8	8
Patients	8494	5717	2777
New patients (% of all patients)	7329 (86%)	4905 (86%)	2424 (87%)
Outcome (% of new patients)			
Benign	4751 (65%)	3441 (70%)	1310 (54%)
Malignant	1322 (18%)	978 (20%)	344 (14%)
Uncertain	1256 (17%)	486 (10%)	770 (32%)
Actual management (% of new patients)			
Surgery	3618 (49%)	2638 (54%)	980 (40%)
Conservative	2826 (39%)	1958 (40%)	868 (36%)
Unknown	885 (12%)	309 (6%)	576 (24%)

Appendix 4 – Prediction models

1. Risk of Malignancy Index (RMI)

The RMI was published in 1990. It is based on data from 143 patients recruited at one hospital in London.[2] RMI is calculated as

$$\text{RMI} = \text{U} * \text{M} * \text{CA125},$$

U is the ultrasound score, M the menopausal score, and CA125 the CA125 level in international units per millilitre. The ultrasound score is based on five ultrasound features: multilocular tumour, presence of solid areas, presence of bilateral tumours, presence of ascites, and presence of intra-abdominal metastases. U has a value of 0 if none of these features is present, 1 if one feature is present, and 3 if more than one feature is present. M has a value of 1 for premenopausal women, and 3 for postmenopausal women. In the original publication describing the RMI, there are no definitions of the variables in the model.[2] When we calculated the RMI, we used the IOTA definitions of multilocular cyst, solid component, and ascites.[7] Centres were encouraged to measure the level of serum CA125 in all patients, but this was not a requirement for inclusion in the study. Measurement of CA125 was left to clinical judgment and local protocols.

2. Logistic Regression model 2 (LR2)

LR2 is a logistic regression model that was published in 2005.[3] It is based on data from 1066 patients recruited at nine centres in Belgium, Sweden, Italy, France, and the United Kingdom. This set was randomly split into a training set of 754 patients, and a test set of 312 patients. LR2 was developed on the training data. The risk of malignancy is based on six clinical and ultrasound characteristics: age of the patient (in years), the presence of ascites (1 vs 0; 'asc'), the presence of papillary projections with blood flow (1 vs 0; 'pfl'), maximum diameter of the largest solid component (in mm, 'mds'), irregular internal cyst walls (1 vs 0; 'icw'), and the presence of acoustic shadows (1 vs 0; 'sha'). LR2 uses the following formula

$$risk = \frac{exp(\text{z})}{1 + exp(\text{z})}$$

Where

$$z = -5.3718 + 0.0354 * \text{age} + 1.6159 * \text{asc} + 1.1768 * \text{pfl}$$
$$+ 0.0697 * \min(\text{mds}, 50) + 0.9586 * \text{icw} - 2.9486 * \text{sha}.$$

Note that the maximum diameter of the largest solid component is winsorised at 50mm: values larger than 50 are set to 50. The ultrasound measurements are performed in accordance with the IOTA 'terms and definitions' statement.[7]

3. Simple Rules (SR)

The Simple Rules are a classification system that is based on five derived ultrasound features that are indicative of a benign tumour, and five derived ultrasound features that are indicative of a malignant tumour.[4] The five benign features are: unilocular cyst ('B1'), presence of solid areas smaller than 7mm in largest diameter ('B2'), presence of acoustic shadows ('B3'), smooth multilocular tumour with largest diameter less than 100mm ('B4'), no vascularization on colour Doppler ('colour score' 1, 'B5'). The five malignant features are: irregular solid tumour ('M1'), presence of ascites ('M2'), presence of at least four papillary projections ('M3'), irregular multilocular-solid tumour with largest diameter above 100mm ('M4'), and very strong vascularisation on colour Doppler ('colour score' 4; 'M5'). Based on which of these ten features apply, SR classifies tumours as benign, inconclusive, or malignant. Tumours are classified as benign if 1 or more benign features apply in the absence of any malignant features. Tumours are classified as malignant if 1 or more malignant features apply in the absence of any benign features. Tumours are classified as inconclusive if no features apply, or if a mix of benign and malignant features apply. In this study, we added inconclusive tumours to the group of tumours classified as malignant, in order to have a dichotomous classification system.

4. Simple Rules risk model (SRRisk)

SRRisk is a logistic regression model published in 2016.[5] It is based on data from 4848 patients recruited at 22 centres in Belgium, Sweden, Italy, Czech Republic, Poland, Spain, United Kingdom, China, and Canada. The model is based on the ten ultrasound features underlying SR (B1 to B5, and M1 to M5; see above), and on the type of centre at which the patient is examined.[5] The ultrasound measurements are performed in accordance with the IOTA 'terms and definitions' statement.[7] Type of centre is a binary variable indicating whether the patient was examined at an oncology centre or not (1 vs 0; 'oc'). Oncology centre is defined as a tertiary referral centre with a specific gynaecological oncology unit. Oncology centres usually have a higher prevalence of malignant tumours, and this should be acknowledged in the risk estimate.

SRRisk is based on a logistic regression model with a random intercept for centre. The final formula sets the random intercepts to zero, and hence uses only the fixed intercept. The formula is

$$risk = \frac{exp(\mathrm{z})}{1 + exp(\mathrm{z})}$$

Where

$$z = -0.9713 - 3.4059 * \mathrm{B1} - 2.2520 * \mathrm{B2} - 1.6633 * \mathrm{B3} - 2.7469 * \mathrm{B4} - 1.8624 * \mathrm{B5} + 2.1933 * \mathrm{M1} + 2.6540 * \mathrm{M2} + 1.5308 * \mathrm{M3} + 0.9806 * \mathrm{M4} + 1.5476 * \mathrm{M5} + 0.9186 * \mathrm{oc}.$$

Note that we used four decimals for every coefficient, whereas the SRRisk publication mentions only two.[5] This has no impact on the results.

5. The Assessment of Different NEoplasias in the adneXa (ADNEX) model

The ADNEX model is a multinomial logistic regression model published in 2014.[6] It is based on data from 5909 patients recruited at 25 centres in Belgium, Sweden, Italy, Czech Republic, Poland, France, Spain, United Kingdom, China, and Canada. ADNEX estimates the risk of five types of tumour: benign, borderline, stage I primary ovarian malignancy, stage II-IV primary ovarian malignancy, and secondary metastatic malignancy. The model is based on nine clinical and ultrasound features: age of the patient (in years), serum CA125 (U/mL), maximum diameter of the lesion (in mm; ‘mdl’), the proportion of solid tissue calculated as the maximum diameter of the largest solid component (in mm) divided by the maximum diameter of the lesion (value between 0 and 1; ‘pst’), presence of more than 10 cyst locules (1 versus 0; ‘tcl’), the number of papillary projections (0, 1, 2, 3, 4, with 4 indicating more than three; ‘nps’), presence of acoustic shadows (1 versus 0; ‘sha’), the presence of ascites (1 versus 0; ‘asc’), and examination at an oncology centre (1 versus 0; ‘oc’). The ultrasound measurements are performed in accordance with the IOTA ‘terms and definitions’ statement.[7] Centres were encouraged to measure the level of serum CA125 in all patients, but this was not a requirement for inclusion in the study. Measurement of CA125 was left to clinical judgment and local protocols.

ADNEX is based on a multinomial logistic regression model with random intercepts for centre. The final formula sets the random intercepts to zero, and hence uses only the fixed

intercepts. A version of ADNEX without CA125 was also developed, because CA125 is not always measured in clinical practice. The formula of ADNEX with CA125 is

$$risk_{benign} = \frac{1}{1 + exp(z_1) + exp(z_2) + exp(z_3) + exp(z_4)}$$

$$risk_{borderline} = \frac{exp(z_1)}{1 + exp(z_1) + exp(z_2) + exp(z_3) + exp(z_4)}$$

$$risk_{stage\ I\ cancer} = \frac{exp(z_2)}{1 + exp(z_1) + exp(z_2) + exp(z_3) + exp(z_4)}$$

$$risk_{stage\ II-IV\ cancer} = \frac{exp(z_3)}{1 + exp(z_1) + exp(z_2) + exp(z_3) + exp(z_4)}$$

$$risk_{secondary\ metastasis} = \frac{exp(z_4)}{1 + exp(z_1) + exp(z_2) + exp(z_3) + exp(z_4)}$$

Where

$$z_1 = -7.577663 + 0.004506 * age + 0.111642 * log2(ca125) + 0.372046 * log2(mdl) + 6.967853 * pst - 5.65588 * pst^2 + 1.375079 * tcl + 0.604238 * nps - 2.04157 * sha + 0.971061 * asc + 0.953043 * onc$$

$$z_2 = -12.276041 + 0.01726 * age + 0.197249 * log2(ca125) + 0.87353 * log2(mdl) + 9.583053 * pst - 5.83319 * pst^2 + 0.791873 * tcl + 0.400369 * nps - 1.87763 * sha + 0.452731 * asc + 0.452484 * onc$$

$$z_3 = -14.91583 + 0.051239 * age + 0.765456 * log2(ca125) + 0.430477 * log2(mdl) + 10.37696 * pst - 5.70975 * pst^2 + 0.273692 * tcl + 0.389874 * nps - 2.35516 * sha + 1.348408 * asc + 0.459021 * onc$$

$$z_4 = -11.909267 + 0.033601 * age + 0.276166 * log2(ca125) + 0.449025 * log2(mdl) + 6.644939 * pst - 2.3033 * pst^2 + 0.89998 * tcl + 0.215645 * nps - 2.49845 * sha + 1.636407 * asc + 0.808887 * onc.$$

For ADNEX without CA125, use

$$z_1 = -7.412534 + 0.003489 * age + 0.430701 * log2(mdl)$$
$$+ 7.117925 * pst - 5.74135 * pst^2 + 1.343699 * tcl + 0.607211 * nps$$
$$- 2.11885 * sha + 1.167767 * asc + 0.983227 * onc$$
$$z_2 = -12.201607 + 0.017607 * age + 0.98728 * log2(mdl)$$
$$+ 10.07145 * pst - 6.17742 * pst^2 + 0.763081 * tcl + 0.410449 * nps$$
$$- 1.98073 * sha + 0.77054 * asc + 0.543677 * onc$$
$$z_3 = -12.826207 + 0.045172 * age + 0.759002 * log2(mdl)$$
$$+ 11.83296 * pst - 6.64336 * pst^2 + 0.316444 * tcl + 0.390959 * nps$$
$$- 2.94082 * sha + 2.691276 * asc + 0.929483 * onc$$
$$z_4 = -11.424379 + 0.033407 * age + 0.560396 * log2(mdl)$$
$$+ 7.264105 * pst - 2.77392 * pst^2 + 0.983394 * tcl + 0.199164 * nps$$
$$- 2.63702 * sha + 2.185574 * asc + 0.906249 * onc.$$

Appendix 5 – Details on imputation and statistical analysis

1. Sample size determination for the IOTA5 study

In light of the primary aim of the IOTA5 study (to investigate the cumulative incidence of complications in patients with an adnexal mass managed conservatively), we aimed to recruit at least 3000 patients with an adnexal mass and at least 1000 patients with an adnexal mass managed conservatively.

For the current model evaluation study, this sample size would be adequate even if the prevalence of malignancy would be as low as 3.3%. With 3.3% prevalence, 100 of the 3000 patients would have a malignancy. This is currently seen as a minimum for evaluating prediction models.[8] The dataset for the primary analysis included 3441 patients with a benign tumour, 978 with a malignant tumour, and 486 with an uncertain outcome. This is sufficient for model evaluation.

2. Multiple imputation of missing values

There were missing values for the CA125 level and for the outcome (reference standard). Measurement of the CA125 level was not mandatory but was highly encouraged. The availability of CA125 depends on local management protocols, and on the clinical and ultrasound characteristics of the patient. Hence, the 'missing at random' mechanism is highly likely: missing values do not occur randomly, but rather depend on variables that are available in the dataset.

Masses in patients that received conservative follow-up could not always be classified as benign or malignant (see main text). In these cases, the reference standard was labelled uncertain. When masses were labelled as malignant based on the clinical information at recruitment and during the first year of follow-up, histology is not available. In these cases, the multinomial reference standard (i.e. type of malignancy) is missing, which is needed for a thorough evaluation of the ADNEX model.

We used multiple imputation with chained equations (mice) to address missing values of CA125 and the multinomial reference standard (benign, borderline, stage I primary malignancy, stage II-IV primary malignancy and secondary metastatic malignancy).[9] We generated 100 imputations, leading to 100 completed datasets. Imputation of CA125 levels was done using predictive mean matching regression. As the distribution of serum CA125 was heavily skewed, the log–log transformation of CA125 was used (i.e., log(log(CA125 + 1))). Imputation of the multinomial reference standard was done using multinomial logistic regression. Variables used to impute CA125 and the multinomial reference standard included variables that are likely to be related to either the true value (had it been observed), to the unavailability of CA125 or the multinomial reference standard (i.e. a binary indicator indicating whether the variable is missing), or variables used in the prediction models that are being validated. Hence, in the imputation model, the following variables were used: patient age (in years), type of centre (oncological versus non-oncological centre), maximum diameter of the lesion (in mm) (log-transformed), proportion of solid tissue (calculated as the

maximum diameter of the largest solid component in mm, divided by the maximum diameter of the lesion in mm) (with a linear and a quadratic term), number of locules (1, 2-10, >10, other), number of papillations (ordinal variable: 0, 1, 2, 3, >3), presence of acoustic shadows (yes/no), presence of ascites (yes/no), presence of metastases (yes/no), bilaterality (yes/no), pelvic pain during examination (yes/no), personal history of ovarian cancer (yes/no), irregular internal cyst walls (yes/no), papillary height (mm), presence of papillary projections with blood flow (yes/no), colour score of intratumoural flow (ordinal variable with four levels 1-4), echogenicity of cyst fluid (nominal variable with 6 levels: anechoic, homogeneous low-level, ground glass, haemorrhagic, mixed, no cyst fluid), CA125 level (log-log transformed), presumed endometrioma (yes/no, according to subjective assessment of ultrasound images by the examiner), subjective assessment at inclusion (6 ordinal groups: certainly benign, probably benign, benign but uncertain, malignant but uncertain, probably malignant, certainly malignant), and the multinomial reference standard. All variables (except the reference standard) were based on the inclusion scan.

Note that some patients are classified as having a malignant tumour based on clinical and ultrasound information during follow-up. For these patients, we do not have a classification into one of the malignancy subtypes: the binary reference standard is ‘malignant’, but the multinomial reference standard is missing. The multiple imputation procedure therefore assumed the multinomial reference standard to be missing such that it was imputed. The most commonly imputed type of malignancy is used as the multinomial reference standard in the analysis.

Each imputation in mice was obtained after 50 iterations. The convergence plots are shown in Figure A4.1. The density plots for log(log(CA125+1)) are shown in Figure A4.2. The distribution of the multinomial reference standard for the observed values (i.e. values that were not missing) and for the missing values after imputation (pooled over the 100 imputations) are shown in Table A4.1.

Figure A4.1. Convergence plots for CA125 and the multinomial reference standard. The plots on the top row refer to CA125 (after log-log transformation; ll_CA125), the plots on the bottom row refer to the multinomial reference standard (CD_5groups). The plots on the left show the mean value, the plots on the right show the standard deviation (sd). The x-axis refers to the iteration (1 to 50). The coloured lines refer to the 100 imputations. The multinomial reference standard was used as a nominal variable in the imputations, despite it being represented as a numerical variable in these plots.

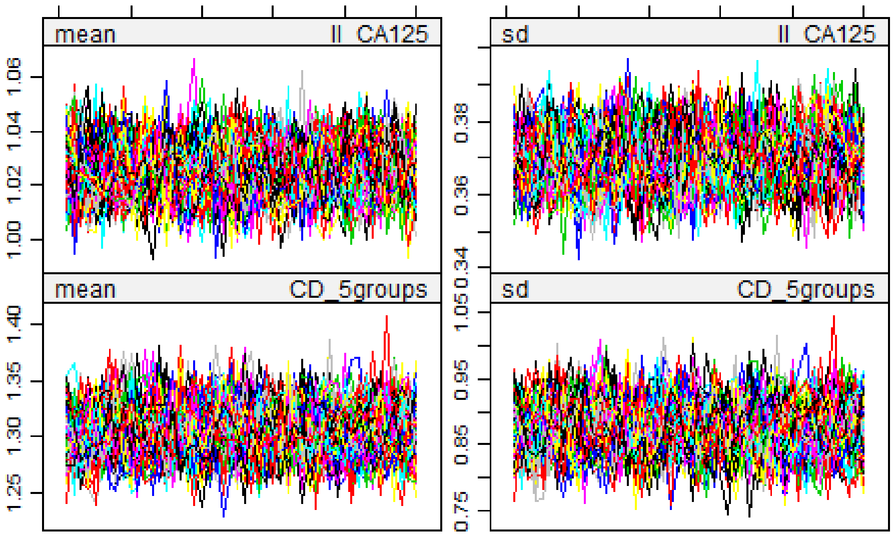
mean
II_CA125
sd
II_CA125
mean
CD_5groups
sd
CD_5groups
1.00
1.02
1.04
1.06
0.34
0.36
0.38
1.25
1.30
1.35
1.40
0.75
0.85
0.95
1.05

Figure A4.2. Density plots of log(log(CA125+1)). The blue curve is the density of the values that were observed (i.e. were not missing), the red curves are the densities for each of the 100 imputations of the values that were missing. The imputed values were less often high than the observed values. ll_CA125, log(log(CA125+1)).

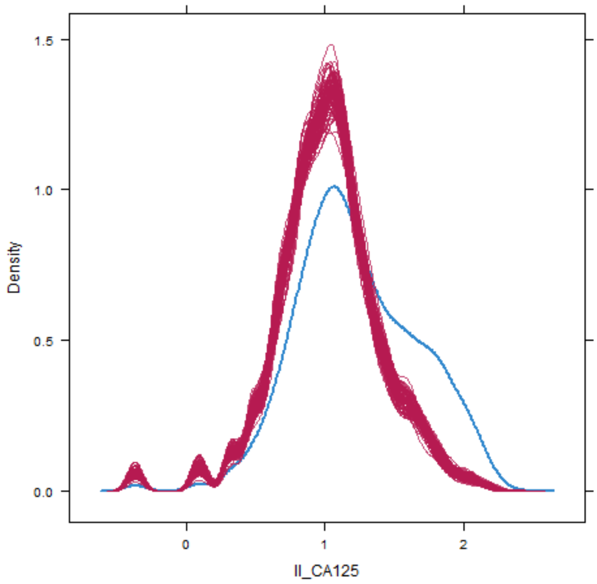

Table A4.1. Distribution of the multinomial reference standard for observed and missing values after imputation. The missing values, after imputation, were more often benign than the observed values.

**Outcome**	**Observed**	**Imputed**
Benign	78%	87%
Borderline	5%	4%
Stage I invasive malignancy	4%	3%
Stage II-IV invasive malignancy	10%	5%
Secondary metastasis	3%	2%

There were three sensitivity analyses: (1) an analysis that excludes masses with uncertain outcome, i.e. U1-4 in Table 1; (2) an analysis in which the definition of an uncertain outcome is expanded to include groups B2, M2-3, and U1-4 in Table 1 (i.e. all groups in which subjective assessment of ultrasound images was used to classify outcomes as benign or malignant); (3) an analysis of patients from all 36 centres that underwent surgery within 120 days after inclusion without any follow-up scan. For the sensitivity analyses, a similar multiple imputation procedure was used. For the first sensitivity analysis, no new imputation procedure was needed. For the second sensitivity analysis, more patients had an uncertain outcome that needed to be imputed. Here, a similar imputation procedure as described above was used to impute missing values for CA125 and the larger amount of missing values for the outcome. For the third sensitivity analysis, the appropriate sample (n=3369) was used to impute missing values for CA125 and for the multinomial reference standard using a similar

procedure as described above (for eight patients, the histology was missing, for 15 patients, the tumour was known to be invasive, but the stage was unknown).

3. Details of the statistical analysis

With respect to the area under the ROC curve (AUC), we obtained the logit(AUC) and its standard error. For this, the auc.nonpara.mw function of the auRoc package was used. In case of multiply imputed data, the logit(AUC) values were combined using Rubin's rules per centre to obtain centre-specific results. 95% confidence intervals for logit(AUC) were calculated, and then the point estimate and the confidence limits were back-transformed to the original scale. An overall AUC was obtained by combining centre-level logit(AUC) and its standard error using random effects meta-analysis. This meta-analysis (including the calculation of the prediction interval) was performed with the rma and predict function in the metafor package.[10] The Sidik-Jonkman estimator was used to estimate the between-study variance.

Based on comments from the reviewers, we additionally calculated 95% confidence intervals for the difference in AUC between any two models. We used the DeLong method for this, through its implementation in the roc.test function of the pROC package.[11,12] To deal with multiply imputed data, Rubin's rules per centre were applied to the estimates and variances obtained from DeLong's test. The resulting centre-specific results were combined with random effect meta-analysis to calculate the 95% confidence interval of the overall difference in AUC between models.

Sensitivity and specificity were analysed as follows. To deal with multiply imputed data, logit-transformed values for sensitivity and specificity were combined using Rubin's rules to obtain centre-specific results. These centre-specific results (logit-transformed) were combined with bivariate random-effects meta-analysis to calculate the overall sensitivity and specificity.[13] The meta-analysis was performed with the rma.mv function in the metafor package.

Regarding calibration, we calculated overall calibration intercept and slope for the risk prediction models, and centre-specific and overall logistic (i.e. non-flexible) calibration curves for the risk prediction models and RMI. We fitted a logistic recalibration model with random intercept and random slope for the J centres:[14]

$logit\left(\frac{P(Y=1)}{1-P(Y=1)}\right) = \alpha + a_j + \beta X + b_j X$, where

$$\begin{bmatrix} a_j \\ b_j \end{bmatrix} \sim N\left(\begin{bmatrix} 0 \\ 0 \end{bmatrix}, \begin{bmatrix} \tau_a^2 & \tau_{ab} \\ \tau_{ab} & \tau_b^2 \end{bmatrix}\right).$$

For LR2, SRRisk, and ADNEX, X is the linear predictor, which equals the logit-transformation of the estimated risk of malignancy. For RMI, X is the natural log of RMI+1. The overall calibration slope equals β. For the calibration intercept, the calibration slopes are set to 1, such that the model reduces to

$logit\left(\frac{P(Y=1)}{1-P(Y=1)}\right) = \alpha' + a_j' + X$, where $a_j' \sim N(0, \tau_{a'}^2)$.

The overall calibration intercept equals α'.

The overall calibration curve is based on the fitted model for the calibration slope where a_j and b_j are set to 0. The centre-specific calibration curve for centre j uses the same fitted model, but with the estimates for a_j and b_j. In case of multiple imputation, the fixed effects (α, β and α') were combined using Rubin's rules, and the centre-specific effects (a_j, b_j and a_j') were averaged.

Regarding clinical utility using decision curve analysis, we calculated Net Benefit (NB) for risk thresholds between 5% and 50% to decide which patients to refer for specialized oncological care.[15,16] For each centre and threshold, we made an average 2x2 cross-tabulation over the 100 imputed datasets. The cross-tabulation contrasts outcome (benign vs malignant) vs classification (risk<threshold vs risk≥threshold). This was used to calculate NB. Using Bayesian trivariate random-effects meta-analysis, the centre-specific NBs at a given threshold were combined into an overall estimate.[17] We used weak realistic priors for separate elements of the between-setting variance-covariance matrix: weak half-normal priors for variances (bounded by zero), weak Fisher priors for correlations and vague normal prior distributions for the remaining parameters. This analysis was performed using WinBugs (https://www.mrc-bsu.cam.ac.uk/software/bugs/the-bugs-project-winbugs/).

For the multinomial evaluation of ADNEX, we did not use meta-analysis methods but a pooled analysis. AUCs between each pair of outcome categories were calculated using the conditional method.[18] Parametric multinomial calibration curves were obtained.[19] These curves were derived per imputed dataset, and averaged. This was done in R using the multiCalibration function in the multiCalibration package (https://repos.openanalytics.eu/html/multiCalibration.html).

For subgroup analyses, we calculated overall AUCs, difference in AUC and calibration curves per model. If sample size allowed, we used the same meta-analysis methods as described above. Else, we used simple pooled estimates, in which case prediction intervals could not be calculated.

For the sensitivity analyses, we calculated centre-specific and overall AUCs, and overall calibration curves per model. We used the meta-analysis techniques described above.

References for the supplementary material

1. Froyman W, Landolfo C, De Cock B, et al. Risk of complications in patients with conservatively managed ovarian tumours (IOTA5): a 2-year interim analysis of a multicentre, prospective, cohort study. *Lancet Oncol* 2019;20:448-58.
2. Jacobs I, Oram D, Fairbanks J, Turner J, Frost C, Grudzinskas JG. A risk of malignancy index incorporating CA125, ultrasound and menopausal status for the accurate preoperative diagnosis of ovarian cancer. *Br J Obstet Gynaecol* 1990;97:922-9.
3. Timmerman D, Testa AC, Bourne T, et al. Logistic regression model to distinguish between the benign and malignant adnexal mass before surgery: a multicenter study by the International Ovarian Tumor Analysis Group. *J Clin Oncol* 2005;23:8794-801.
4. Timmerman D, Testa AC, Bourne T, et al. Simple ultrasound-based rules for the diagnosis of ovarian cancer. *Ultrasound Obstet Gynecol* 2008;31:681-90.
5. Timmerman D, Van Calster B, Testa A, et al. Predicting the risk of malignancy in adnexal masses based on the Simple Rules from the International Ovarian Tumor Analysis group. *Am J Obstet Gynecol* 2016;214:424-37.
6. Van Calster B, Van Hoorde K, Valentin L, et al. Evaluating the risk of ovarian cancer before surgery using the ADNEX model to differentiate between benign, borderline, early and advanced stage invasive, and secondary metastatic tumours: prospective multicentre diagnostic study. *BMJ* 2014;349:g5920.
7. Timmerman D, Valentin L, Bourne T, Collins WP, Verrelst H, Vergote I. Terms, definitions and measurements to describe the sonographic features of adnexal tumors: a consensus opinion from the International Ovarian Tumor Analysis (IOTA) group. *Ultrasound Obstet Gynecol* 2000;16:500-5.
8. Moons KGM, Altman DG, Reitsma JB, et al. Transparent Reporting of a multivariable prediction model for Individual Prognosis or Diagnosis (TRIPOD): explanation and elaboration. *Ann Intern Med* 2015;162:W1-73.
9. van Buuren S, Groothuis-Oudshoorn K. “mice: Multivariate Imputation by Chained Equations in R. *J Stat Softw* 2011;45(3):1-67.
10. Viechtbauer W. Conducting Meta-Analyses in R with the metafor Package. *J Stat Softw* 2010;36(3):1-48.
11. Demler OV, Pencina MJ, D’Agostino RB Sr. Misuse of DeLong test to compare AUCs for nested models. *Stat Med* 2012;31:2577-87.
12. Robin X, Turck N, Hainard A, et al. pROC: an open-source package for R and S+ to analyze and compare ROC curves. BMC *Bioinformatics* 2011;12:77.
13. Reitsma JB, Glas AS, Rutjes AW, Scholten RJ, Bossuyt PM, Zwinderman AH. Bivariate analysis of sensitivity and specificity produces informative summary measures in diagnostic reviews. *J Clin Epidemiol* 2005;58:982-90.
14. Wynants L, Vergouwe Y, Van Huffel S, Timmerman D, Van Calster B. Does ignoring clustering in multicenter data influence the performance of prediction models? A simulation study. *Stat Methods Med Res* 2018;27:1723-36.
15. Wynants L, Timmerman D, Verbakel JY, et al. Clinical utility of risk models to refer patients with adnexal masses to specialized oncology care: multicenter external validation using decision curve analysis. *Clin Cancer Res* 2017;23:5082-90.
16. Van Calster B, Wynants L, Verbeek JFM, et al. Reporting and Interpreting Decision Curve Analysis: A Guide for Investigators. *Eur Urol* 2018;74:796-804.
17. Wynants L, Riley R, Timmerman D, Van Calster B. Random-effects meta-analysis of the clinical utility of tests and prediction models. *Stat Med* 2018;37:2034-52.

18. Van Calster B, Vergouwe Y, Van Belle V, Looman CWN, Timmerman D, Steyerberg EW. Assessing the discriminative ability of risk models for more than two outcome categories: a perspective. *Eur J Epidemiol* 2012;27:761-70.
19. Van Hoorde K, Vergouwe Y, Timmerman D, Van Huffel S, Steyerberg EW, Van Calster B. Assessing calibration of multinomial risk prediction models. *Stat Med* 2014;33:2585-96.
